# Phylogenomic and Pangenomic Assessment of a Mediterranean Strain of *Raphidiopsis raciborskii* Extends Knowledge of the Global Distribution and Characteristics of a Potentially Toxigenic Cyanobacterium

**DOI:** 10.1111/1758-2229.70098

**Published:** 2025-05-19

**Authors:** Nico Salmaso, Leonardo Cerasino, Margherita Di Brizio, Massimo Pindo, Adriano Boscaini

**Affiliations:** ^1^ Research and Innovation Centre Fondazione Edmund Mach San Michele all'Adige Italy; ^2^ NBFC National Biodiversity Future Center Palermo Italy; ^3^ UOLM Sito Perugia ARPA Umbria Perugia Italy

**Keywords:** cyanotoxins, *Cylindrospermopsis*, genome mining, metabolic reconstruction, pangenomics, phylogenomics, *Raphidiopsis*

## Abstract

Among potentially toxigenic cyanobacteria, *Raphidiopsis raciborskii* has attracted considerable attention due to its ability to produce massive blooms and its recent spread to temperate regions. In this work, we reported for the first time a taxonomic and functional assessment of a *R. raciborskii* strain isolated from the Mediterranean region, contributing to filling a gap in the global distribution and characteristics of this species. The strain LT_0923 was isolated from Lake Trasimeno, a large and shallow lake in central Italy. The phylogenomic analyses based on selected marker genes and the core genome obtained from a pangenomic analysis based on a selection of available high‐quality genomes showed a strong correspondence of the Lake Trasimeno strain with the North American and, at a lower average nucleotide identity, with the South American genomes. The LT_0923 genome did not show the presence of gene clusters encoding legacy cyanotoxins or emerging toxigenic compounds. The open pangenome and the large fraction of distinct gene families identified in the cloud and partly shell genome, enriched with genes specialised in environmental‐specific functions and defence mechanisms, are consistent with the development of *Raphidiopsis* in geographically distinct regions.

## Graphical abstract

The potentially toxigenic cyanobacterium *Raphidiopsis raciborskii* has spread to temperate regions and produces massive blooms. This study reports the first taxonomic and functional metagenomic assessment of a strain from the Mediterranean, thus filling a significant gap in global distribution. The Lake Trasimeno strain shows similarities to North and South American genomes.

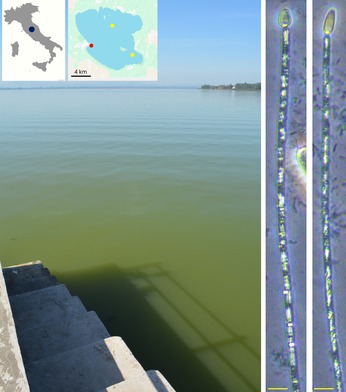

## 1 Introduction

Among the potentially toxigenic cyanobacteria, *Raphidiopsis raciborskii* (Woloszynska), Aguilera et al. has received rapid and increasing attention due to its recent spread to temperate regions and ability to develop massive blooms and toxic strains (Padisák 1997; Antunes et al. 2015).

This cyanobacterium was originally included in the genus *Cylindrospermopsis* and was distinguished from *Raphidiopsis* by its ability to produce heterocytes. Nevertheless, as this species is characterised by great morphological diversity and phenotypic plasticity, in the absence of heterocytes it did not show any other clear diacritical features to distinguish it from *Raphidiopsis* (Komárek and Mareš 2012). In addition, several genetic and phylogenetic studies showed that species of *Raphidiopsis* and *Cylindrospermopsis* formed a polyphyletic group, suggesting the evolution of populations that have lost the ability to produce heterocytes (Moustaka‐Gouni et al. 2009). Based on these and new evidence, Aguilera et al. (2018) proposed the unification of these two genera under the name *Raphidiopsis*, respecting the principle of priority (Komárek and Mareš 2012).

Considered to be of tropical origin, the rapid spread of *R. raciborskii* to temperate regions has been documented in several countries (Padisák 1997). The first record of *R. raciborskii* in Europe was documented in Lake Kastoria, Greece (Skuja 1937) and then in Hungary. Since then, the cosmopolitan presence of this species has been increasingly reported in several countries of temperate regions (Antunes et al. 2015; Kokociński et al. 2017). In Italy, *R. raciborskii* was reported for the first time in 1995 in Lake Trasimeno (Manti et al. 2005), and then in several other lakes throughout Italy (Del Pasqua et al. 2024; Austoni et al. 2025).

Several phylogenetic studies based on individual marker genes highlighted the existence of different genetic populations among *R. raciborskii* from various geographical regions (Haande et al. 2008). Among the most recent investigations, Moreira et al. (2015) showed a congruence between strains from Europe, Asia and Australia, separated from the American and North African clusters, suggesting that the strains from Europe originated from Asia and Australia. After adding to the analyses other strains from southern Europe, Cirés et al. (2014) showed a strong link between the populations of Central and North America and Tunisia with strains isolated from Spain, all more distantly related to other strains from Portugal and Northern Europe, as well as other continents. These results, which demonstrated a genetic inhomogeneity of *R. raciborskii* populations in Europe, were further confirmed by Panou et al. (2018), who demonstrated that several Greek strains of this species formed a compact cluster with strains from Spain, Tunisia and America.

The success of *R. raciborskii* is attributed to its phenotypic plasticity and to the development of ecotypes adapted to local environmental constraints, which gave rise to the existence of populations with defined biogeographic patterns (Vico et al. 2021). One of the most striking differences between populations is the ability to evolve diverse strains capable of producing different types of cyanotoxins on different continents. Cylindrospermopsin (CYN) producing strains have been isolated from Australia (Saker and Griffiths 2000), New Zealand (Wood and Stirling 2003) and Asia (Li et al. 2001; Zarenezhad et al. 2012; Rzymski and Poniedziałek 2014; Nguyen et al. 2017), while saxitoxins (STXs) producing strains have been isolated from South America (Lagos 2003; da Costa et al. 2013; Mesquita et al. 2019). Conversely, only very rare STX or CYN‐producing strains were identified in Europe (Sha et al. 2025).

The full range of results reported above was based on target analyses performed on selected gene markers and analytical determinations on numerous isolated strains from different continents. As a downside, target approaches are necessarily restricted to a limited number of marker genes, with functional analyses limited to only a few selected metabolites.

Owing to a much more limited available number of strains, the relationships between *R. raciborskii* populations analysed using high‐throughput sequencing and genomic approaches were restricted to selected regions, particularly Australia, Asia and South America (Stucken et al. 2010; Sinha et al. 2014; Abreu et al. 2018; Willis et al. 2018; Willis and Woodhouse 2020). Information from other American regions and Europe was limited to a few genomes, respectively from North America and Central Europe (Vico et al. 2020; Laux et al. 2023; Tokodi et al. 2025), while no genomes are available for the Mediterranean regions. Given the strong relationships between *R. raciborskii* strains from North America and some Mediterranean regions based on the use of single marker genes, this still represents an important knowledge gap in the results obtained from phylogenomic and pangenomic analyses.

In this work, we report a taxonomic and functional assessment of a *R. raciborskii* strain isolated from Lake Trasimeno, a large and shallow lake in central Italy. The characteristics of this Mediterranean strain will be compared with an extended set of high‐quality genomes including recently published strains from Central Europe and North America. Specific objectives of this work include: (i) the taxonomic and functional genomic analysis of the Lake Trasimeno strain; (ii) the phylogenomic characterisation of this Mediterranean strain in relation to representative high‐quality genomes isolated worldwide; (iii) through a pangenomic analysis to evaluate the main differences in the entire set of encoded proteins among *R. raciborskii* strains; (iv) further attention will be given to the potential ability to synthesise cyanotoxins and other secondary metabolites.

## 2 Materials and Methods

### 2.1 Study Site, Sampling Stations and Field and Laboratory Measurements

Lake Trasimeno is a large (≈ 124 km^2^ and 586 × 10^6^ m^3^) and shallow (*z*_max_ ≈ 6 m) lake located at 258 m a.s.l. in central Italy. The lake level can vary considerably in response to atmospheric precipitation, which influences the hydrodynamic conditions and the size of the lake. Lake Trasimeno is endorheic with no natural outlet, and an artificial canal flowing into the Tiber River. Trophic status is between mesotrophy and eutrophy (Ludovisi et al. 2021). Based on satellite data, since the 1980s the lake has shown a significant warming at a rate of around 0.017°C yr^−1^ (Pareeth et al. 2017).

Sampling was carried out on September 12, 2023 at the boat pier located at Castiglione del Lago (CL) (43°07′23.6″ N, 12°03′26.5″ E; Figure S1A). Samples were collected at the surface using a sterilised bucket and nets (mesh size 80 μm). Subsamples were stored in refrigerated conditions until the isolation of *Raphidiopsis*. A subsample (250 mL) for phytoplankton analyses was fixed soon after collection with Lugol's solution and successively stored in the laboratory at 4°C. At the sampling point, water temperatures were measured using a handheld thermometer and transparency using a Secchi disk. More complete physical and chemical analyses, representative of the general conditions of the lake, were performed the day before (September 11, 2023) in two pelagic stations by the laboratories of the ARPA Umbria. The analyses and methods are publicly available at https://www.arpa.umbria.it (for details, see Figure S1A).

### 2.2 Cyanotoxins Analyses

After filtration on GF/C filters of 0.4 L from water samples and 0.3 L from net samples, cyanotoxins were quantified as described by Cerasino and Salmaso (2020) and Salmaso et al. (2024). Analytical determinations did include several microcystin (MCs) variants, anatoxins (ATXs; ATX‐a and homoATX‐a), cylindrospermopsin (CYN) and saxitoxins (STXs; STX, dc‐STX, NeoSTX, GTX1, GTX4, GTX5, C1 and C2).

### 2.3 Microscopical Observations

Field and culture samples were observed under an invertoscope Zeiss Axiovert 135 and an upright microscope Leica DM 2500. The counting and measurement of the *Raphidiopsis* cells from the field sample were performed using the Utermöhl method (Lund et al. 1958), as described in detail in Rott et al. (2007).

### 2.4 Isolation, DNA Extraction and Sequencing

Single filaments of *Raphidiopsis* were isolated using micropipettes under stereomicroscopes within 1 day after the sampling, and isolates were incubated in Z8 medium (Kotai 1972) at 20°C under a 12 h:12 h light–dark photoperiod (70 μmol m^−2^ s^−1^) in climatic simulation chambers (ProClimatic, Italy); complete general isolation procedures are described in Salmaso et al. (2016).

The non‐axenic culture of the *R. raciborskii* strain isolated from Lake Trasimeno was filtered through 8 μm cellulose nitrate membranes (Cytiva) and DNA was extracted using the PowerWater Kit (Qiagen). The library was prepared using the Kapa HyperPlus Kit (Roche) and processed by 150‐bp paired‐end sequencing on an Illumina NovaSeq 6000 (Illumina Inc., San Diego, CA, USA) following the procedures described in Salmaso et al. (2024).

An aliquot of the extracted DNA from the isolate was used to amplify the 16S rRNA gene using the primers pA and B23S, successively sequenced using the internal primers 16S544R, 16S1092R and 16S979F, as described in Rajaniemi‐Wacklin et al. (2005) and Salmaso et al. (2015) (GenBank accession number, PV054108).

### 2.5 Draft Bacterial Genomes: Binning and Assembly

Assembling and binning followed the procedures described in detail in Salmaso et al. (2024), using the most up‐to‐date package versions. In short, raw reads were checked with FastQC 0.12.1 (github.com/s‐andrews/FastQC). Removal of residual adapters and trimming were performed using bbduk 39.05 (https://jgi.doe.gov), keeping only reads with quality scores greater than 20 (trimq = 20 and minavgquality = 20). After removal of human reads contaminants (mapping of reads on the human reference genome GRCh38.p14 using Bowtie 2.5.2 and filtering with SAMtools 1.19) (Langmead and Salzberg 2012; Danecek et al. 2021), the successive analyses were performed on 10% of the quality checked and processed paired‐end reads. Before assembling, a preliminary assessment of the taxonomic composition and relative abundance of the microbial community was carried out using MetaPhlAn 4.0.6 with the ‐‐unclassified_estimation parameter (Blanco‐Míguez et al. 2023).

Paired reads were corrected with metaSPAdes 4.0.0 (‐‐only‐error‐correction) (Nurk et al. 2017) and assembled with megahit 1.2.9 (‐‐presets meta‐sensitive) (Li et al. 2015). Before binning, contigs shorter than 1000 bp were discarded and the names of the remaining contigs were simplified with anvi'o 8 (Eren et al. 2021). The resulting contigs were binned using CONCOCT 1.1.0 (Alneberg et al. 2014), MetaBAT 2.17–21 (Kang et al. 2019) and SemiBin2 2.1.0 (Pan et al. 2022). The resulting bins were then combined using DAS Tool 1.1.6 (Sieber et al. 2018), using default options, and assessed for the presence of chimerism using GUNC 1.0.6 (Orakov et al. 2021) and other sources of contamination using MDMcleaner 0.8.7 (Vollmers et al. 2022). The final *R. raciborskii* bin was checked and confirmed with anvi'o 8. Completeness and redundancy of the assemblies were estimated using CheckM2 1.0.2 (Chklovski et al. 2023) and coverages using CoverM 0.6.1 (github.com/wwood/CoverM).

The Genome Shotgun project obtained from the isolate has been deposited at DDBJ/ENA/GenBank under the project number PRJNA1215010.

### 2.6 Phylogenomic, Functional and Pangenomic Analyses

Genomes to be compared with the *R. raciborskii* strain isolated from Lake Trasimeno were selected to cover all the *Raphidiopsis* species available in the Genome Taxonomy Database (GTDB; release 09‐RS220) (Parks et al. 2022), integrated with assemblies from NCBI (Schoch et al. 2020). From this initial set, a subset of genomes with completeness greater than 94% and/or contamination less than 4% (as determined by CheckM2) and a number of contigs less than 1000 were retained for subsequent analyses (Table S1). Other redundant/duplicated genomes (GCA_000175835.1), genomes with length too small (less than 3 Mbp) and genomes with ambiguity codes were excluded from the analysis.

The *R. raciborskii* genome from Lake Trasimeno was compared with the selected *Raphidiopsis* spp. genomes using the average nucleotide identity (ANI) computed using average_nucleotide_identity.py and the ANI_b_ option from the package pyani 0.3.0‐alpha (Pritchard et al. 2016); the reciprocal ANI_b_ comparisons between a couple of genomes were then averaged to obtain one unique value. ANI values between 0.95 and 0.96 have been proposed as a boundary to identify genomes belonging to the same species (Rosselló‐Móra and Whitman 2019), while genomes of different species generally have ANI < 0.90 and ANI values in the range 0.90–0.95 are comparatively rare (Rodriguez‐R et al. 2024). Phylogenomic analyses were carried out using GTDB‐Tk 2.4.0 (identify, align, infer commands) (Chaumeil et al. 2022), based on 120 markers (Hidden Markov Models, HMMs) specifically designed for the analysis of bacteria, alignment using hmmalign (Eddy 2011) and tree building using FastTree (Price et al. 2010). The tree topology was confirmed using GToTree 1.8.8 based on pre‐packaged HMM single‐copy genes set specific for Cyanobacteria (251 genes) (Lee 2019), alignment using muscle 5.0 (Edgar 2022), and tree building using IQTree (‐m MFP ‐B 1000) (Nguyen et al. 2015). In both cases, the trees were rooted using the python package ETE3 (Huerta‐Cepas et al. 2016) and *Sphaerospermopsis kisseleviana* CS‐549 (GCA_028329225.1) as an outgroup. The final trees were built and annotated using the R package ggtree 3.10.1 (Yu 2020).

Functional annotation of the *R. raciborskii* draft genome was performed using the NCBI stand‐alone software package Prokaryotic Genome Annotation Pipeline (PGAP) version 2024‐07‐18.build7555 (Li et al. 2020) and finally confirmed by annotation using the PGAP service in NCBI (https://www.ncbi.nlm.nih.gov/). Annotations were complemented using bakta 1.9.4 (Schwengers et al. 2021). Target sequences for the corresponding intervals defined in the PGAP/bakta feature tables were extracted from the *R. raciborskii* genome using bedtools 2.31.1 (Quinlan and Hall 2010). Identification of ribosomal rRNA genes was further evaluated using Barrnap 0.9 (github.com/tseemann/barrnap). ARGs in all the *Raphidiopsis* genomes were predicted using AMRFinderPlus 4.0.3 with database version 2024‐12‐18.1 (Feldgarden et al. 2021) and ABRicate (version 1.0.1), incorporating the NCBI AMRFinder, ARG‐ANNOT, ResFinder and Card databases (github.com/tseemann/abricate) and using minimum DNA identity and coverage values of 80% and 50%, respectively.

Metabolism and phenotypic features of the *Raphidiopsis* genomes were identified using the metabolic reconstruction set of programs in anvi'o 8 (Eren et al. 2021; Watson et al. 2023), with the options ‐E 1e‐15 and ‐H 0.95 in anvi‐run‐kegg‐kofams. Functional annotations were based on the Kyoto Encyclopedia of Genes and Genomes (KEGG) (Kanehisa et al. 2014). K numbers were assigned to sequence data by HMMER/HMMSEARCH against Kofam, a customised HMM database of KEGG Orthologs (KOs) (Aramaki et al. 2020). KOs functional annotations were then matched to metabolic pathways (modules) (Kanehisa and Sato 2020) and their completeness was estimated using the program anvi‐estimate‐metabolism. Functional enrichment was finally computed to check if specific modules were significantly enriched in contrasting groups of genomes (Shaiber et al. 2020).

Pangenomic analysis was performed including all the *Raphidiopsis* genomes following the anvi'o 8 pangenomic workflow (Delmont and Eren 2018; Eren et al. 2021). After associating genes in the respective anvi'o contigs database with COGs (COG20) functions (Tatusov et al. 1997), a pangenome was generated using anvi‐pan‐genome with the option ‐‐mcl‐inflation 7 (van Dongen and Abreu‐Goodger 2012) to identify gene clusters. Evolutionary associations in the pangenome were assessed by computing a phylogenomic analysis using 1526 single‐copy core genes (SCGs) across all genomes. After removing gap characters in more than 50% of the sequences using trimAl v1.4.rev15, a tree was built using IQTree (‐m MFP) and midpoint rooted using the ETE3 package. The phylogenomic tree based on the SCGs was compared with the corresponding GTDB‐Tk tree by means of a co‐phylogenetic tree using Phytools 2.4.4 (Revell 2024). A parallel pangenomic analysis was further performed using panaroo 1.5.1 (Tonkin‐Hill et al. 2020). A heatmap showing the distribution of the main COGs categories was performed using the R package heatmaply 1.5.0. To estimate if the *Raphidiopsis* pangenome was open or closed, the Heaps law was applied to the results obtained with anvi'o and panaroo; in the Heaps model, the relationship between new genes discovered with the addition of new genome sequences is modelled by a power‐law function, *n* = kN^γ^, where *n* = pan‐genome size, *N* = number of genomes and k and γ are fitting parameters (Tettelin et al. 2008). The pangenome is closed and approaches a constant size as more genomes are used when *γ* < 0, whereas when *γ* > 0, the pangenome is open and increases in size as more genomes are included. Heaps parameters were estimated with 1000 genome random samples using the specaccum function in the R package vegan 2.6.10 (Oksanen et al. 2020), and a least‐squares fit of the power law to the medians calculated for each distribution, following the approach of Tettelin et al. (2008).

The presence of secondary metabolite biosynthetic gene clusters (BGCs) in all the *Raphidiopsis* genomes was estimated using antiSMASH 7.1.0 (default mode) (Blin et al. 2023). The presence of selected genes encoding MC, ATX‐a, CYN and STX, as well as odorous compounds (geosmin) was tested using ISeqDb 3.0 (github.com/hts‐tools/iseqdb).

## 3 Results

### 3.1 Environmental Data

In the littoral sampling site, surface water temperatures and transparency were 24.6°C and 0.3 m, respectively. The general condition of the lake during sampling is exemplified by the data collected by ARPA Umbria in the two pelagic stations, which showed equivalent temperature and transparency values to those measured on the littoral shore (Table 1; Figure S1). The high trophic status of the lake is confirmed by the high concentrations of SRP and TP (17–18 and 70–80 μg P L^−1^, respectively), chlorophyll a (44–50 μg L^−1^), pH (9) and dissolved oxygen (110%–126%). Conversely, nitrogen compounds always showed concentrations < 0.1 mg L^−1^ (NO_3_‐N and NH_4_‐N) and < 2 mg L^−1^ (total nitrogen).

**TABLE 1:** Physical and chemical characteristics of surface samples collected on September 11, 2023 in the two pelagic stations of Lake Trasimeno (Figure S1).

Sampling station	Passignano sul Trasimeno	Magione
Latitude	43°09′17.1″ N	43°06′18.0″ N
Longitude	12°06′46.6″ E	12°09′34.0″ E
Temperature (°C)	25.0	25.0
Conductivity (μS cm^−1^) 20°C	1826	1841
pH	9.0	9.0
Oxygen (mg L^−1^)	10.1	8.8
Nitrate nitrogen, NO_3_‐N (mg L^−1^)	< 0.10	< 0.10
Ammonium nitrogen, NH_4_‐N (mg L^−1^)	< 0.04	< 0.04
Total nitrogen, TN (mg L^−1^)	1.3	1.7
Soluble reactive phosphorus, SRP (μg L^−1^)	17	18
Total phosphorus, TP (μg L^−1^)	80	70
Chlorophyll a (μg L^−1^)	43.9	50.0
Secchi disk depth (m)	0.4	0.3

### 3.2 Cyanotoxins

The LC–MS analyses did not show any quantifiable presence of cyanotoxins in all the water and net samples analysed.

### 3.3 Light Microscopy

The filaments observed in the environmental samples collected in the littoral station and the isolated strain (Figure S1B) showed the diacritical characters typical of *R. raciborskii* (Manti et al. 2005; Mugnai et al. 2008; Karadžić et al. 2013; Komárek 2013). In the lake littoral sample, the length of the *Raphidiopsis* filaments was generally between 40 and 200 μm. The density was high (164,000 cells mL^−1^), with akinetes present in around 10% of the filaments and with length 8–15 μm and width 3–4 μm. Heterocytes were always terminal and present in around 7% of the filaments.

### 3.4 Lake Trasimeno *Raphidiopsis* Genome

Novaseq sequencing of the genomic DNA extracted from the *Raphidiopsis* culture generated 89,169,269 paired‐end reads with 97.3% and 92.4% of bases with average quality scores greater than 20 and 30, respectively. Raw data quality processing removed 5% of the raw reads. The preliminary assessment of the community represented in the non‐axenic culture by metaphlan showed a large majority of quality processed reads belonging to *Cylindrospermopsis* (*Raphidiopsis*) *raciborskii* (91%), followed by unclassified sequences (8%). The other few reads were mostly classified in the genera *Brevundimonas* (0.7%) and *Flavobacterium* and *Sandarakinorhabdus* (0.01%–0.02%).

After resampling, the successive analyses were performed on 8,434,616 quality‐checked and processed paired‐end reads, of which 92.3% was attributed by metaphlan to *Cylindrospermopsis*. Following correction with metaSPAdes, assembly with Megahit yielded 1250 contigs larger than 1000 bp, with a total size of 5.83 Mbp and N50 of 29,680 bp. The Lake Trasimeno *Raphidiopsis* strain was 3.46 Mbp assembled into 87 contigs, with N50 139,068 bp, GC content 39.94% and coverage 655×; based on CheckM2, completeness and contamination estimates were 99.8% and 0.04%, respectively (Table 2).

**TABLE 2:** Summary of statistics from the Lake Trasimeno *Raphidiopsis raciborskii* genome assembly.

Variable	
Total length (bp)	3,455,381
No. of contigs	87
GC content (%)	39.94
Mean coverage (×)	655
Size of longest contig (bp)	350,162
N50 (bp)	139,068
No. of protein‐coding genes	3095
No. of tRNA	38
Complete/partial 5S rRNA	1/1
Complete/partial 16S rRNA	0/1
Complete/partial 23S rRNA	1/0
Completeness (CheckM) (%)	99.93
Contamination (CheckM) (%)	0.44
Strain heterogeneity (CheckM) (%)	0.0
Completeness (CheckM 2) (%)	99.81
Contamination (CheckM 2) (%)	0.04

Following the PGAP analysis, besides 3095 protein‐coding genes, the Lake Trasimeno strain included 38 tRNAs, complete sequences of 5S rRNA and 23S rRNA and incomplete sequences of 5S rRNA and 16S rRNA (Table 2). After blastn analyses, all these sequences showed a high percentage identity to different *Cylindrospermopsis* and *Raphidiopsis* sequences belonging to strains isolated globally (Table S2). In addition to the short 5S rRNA (114–118 bp) and the partial 16S rRNA (120 bp) sequences, the results obtained by querying the core_nt database were strongly biased by both the incompleteness of some sequences and the incomplete geographical coverage, especially in the markers that were not the object of phylogenetic studies. The results were much more consistent and comparable when querying the Whole Genome shotgun contigs (wgs) database (Table S2); excluding the shortest 5S rRNA and the incomplete16S rRNA sequences, all the markers showed perfect or high correspondence with strains from several North American water bodies.

The long 16S rRNA sequence (1485 bp) identified in the unbinned contigs (Table S2C) was 100% identical to the 16S rRNA sequence determined by Sanger sequencing of both the strain 1LT32S01 isolated from Lake Trasimeno in 2002 by Mugnai et al. (2008) and the strain isolated from Lake Trasimeno in this work (LT_0923; accession number PV054108).

Based on the GTDB taxonomy and GTDB‐Tk results, the closest genome reference of the Lake Trasimeno strain was *R. brookii* D9 (ANI screen, 0.952). Considering the ANI_b_ computations performed on the whole set of genomes analysed in this work, the closest genomes (ANI_b_ > 0.99) to the Lake Trasimeno isolate were the two genomes from North America, that is, *Cylindrospermopsis raciborskii* KL1 and Lake Mendota metagenome assembled genome (MAG) *R. brookii* (GCA_044352285.1). All the other ANI_b_ to the other genomes were between 0.922 and 0.960.

### 3.5 Metabolomic Functions of the Lake Trasimeno *Raphidiopsis* Strain

The complete and incomplete pathway modules reconstructed from the KO numbers identified in the Lake Trasimeno LT_0923 strain did include several categories represented by the metabolism of cofactors and vitamins (42), amino acid metabolism (41), energy metabolism (33) and carbohydrate metabolism (31), whereas the remaining pathways included from 2 to 12 modules (Table S3). Reactions required for the central metabolisms of photosynthetic cyanobacteria were represented by oxygenic photosynthesis (photosystems II and I; modules M00161 and M00163), including phycobilisomes (allophycocyanin and phycocyanin/phycoerythrocyanin), beta‐carotene biosynthesis (M00097), the reductive pentose phosphate cycle (Calvin cycle) (M00165), the tricarboxylic acid (Krebs) cycle (M00009) and glycolysis (M00001, M00002).

Nitrogen metabolism was sustained by assimilatory nitrate reduction (M00531) and nitrogen fixation (M00175). Specifically, besides the *nifH* gene, which is commonly used as a marker of taxonomic relevance, the ability to fix nitrogen was confirmed by the presence of 11 different *nif* genes identified by PGAP and part of the core and auxiliary set of genes involved in the fixation of atmospheric nitrogen. Nitrogen assimilation was further supported by potential ammonium uptake (K03320; *amt* gene) and by genes encoding nitrate/nitrite transporter (nrtABD), part of the ATP‐binding cassette (ABC) membrane transporters. No other reactions associated with dissimilatory N‐reduction, denitrification, nitrification and anammox were identified.

ABC transporters were also involved in the transport of a broad range of different microelements, nutrients and organic molecules; most of them were classified as complete by KEGG Mapper. Within the C‐metabolism, the bicarbonate transporter CmpABCD was involved in the cell carbon concentration (CCM). The P‐metabolism was supported by several genes involved in the active transport of phosphate (PstSCAB) and organophosphorus compounds (phosphonate) (PhnDEC), completed by Pho regulon components (PhoHURB; K06217, K02039, K07636 and K07657) involved in the regulation of P‐uptake. Besides the ABC transporter for sulfate/thiosulfate (Cys/Sbp), a gene encoding a non‐ABC lower‐affinity transport sulfate permease was also detected (K03321, SulP family). The availability of molybdate, the main bioavailable form of molybdenum, required for many enzymatic reactions (such as N‐ and S‐metabolism), was sustained by the ModABC high‐affinity transport system. Another set of genes was involved in the selective transport of organic molecules, including oligosaccharides (chitobiose, ChiEFG), phospholipids (mlaDEF), amino acids (General L‐Aminoacid, Branched‐chain amino acid, Neutral amino acid/Histidine), the rich‐N urea (UrtABCDE) and lipopolysaccharides (LptFGB). The availability of several microelements was potentially supported by selected ABC transporters (complete or incomplete) for zinc (ZnuAB), cobalt (CbiNMQO) and nickel (CbiN/LMQO). The potential uptake of osmoprotectants was indicated by the presence of the OpuBCBBBA ABC transport system.

A primary role in the maintenance of cellular functionality was potentially played by several modules in the biosynthesis of cofactors and vitamins involved in various biochemical reactions, such as several vitamins with complete or almost complete modules (≥ 0.75) (B12, cobalamin; K1, phylloquinone; and E, Tocopherol/tocotorienol).

The removal of reactive oxygen species (ROS) superoxide (O_2_^−^) and hydrogen peroxide (H_2_O_2_) produced as byproducts by photosynthesis was directly or indirectly supported by a set of enzymes encoding superoxide dismutase, catalase, catalase‐peroxidase, glutathione peroxidase, peroxiredoxins and other enzymes involved in the regulation of oxidative stress response (K04564, K24157, K24158, K00799, K01920, K00383, K09825).

The ability to control vertical migration was confirmed by the presence of genes encoding proteins forming gas vesicles (PGAP analysis: GvpA, C, F, G, J, L, N).

The analysis of the presence of gene clusters encoding secondary metabolites confirmed the absence of operons encoding legacy cyanotoxins. Furthermore, the Lake Trasimeno *Raphidiopsis* genome did not show the presence of any *mcyBDE*, *anaCF*, *cyrABJ* and *sxtAI* residual sequence. In addition to heterocyst glycolipids, which are associated with heterocyst functionality and N‐fixation, and a very low similarity (8%) with arylpolyene, the only genomic region showing similarity to a known cluster was associated with the production of hassallidin, while other regions were connected to unknown lanthipeptides and terpenes and one NRPS.

No ARGs were identified in the genome of the *R. raciborskii* isolated from Lake Trasimeno.

### 3.6 Phylogenomic Analyses and Metabolomic Potential of *Raphidiopsis* Strains

**FIGURE 1:**
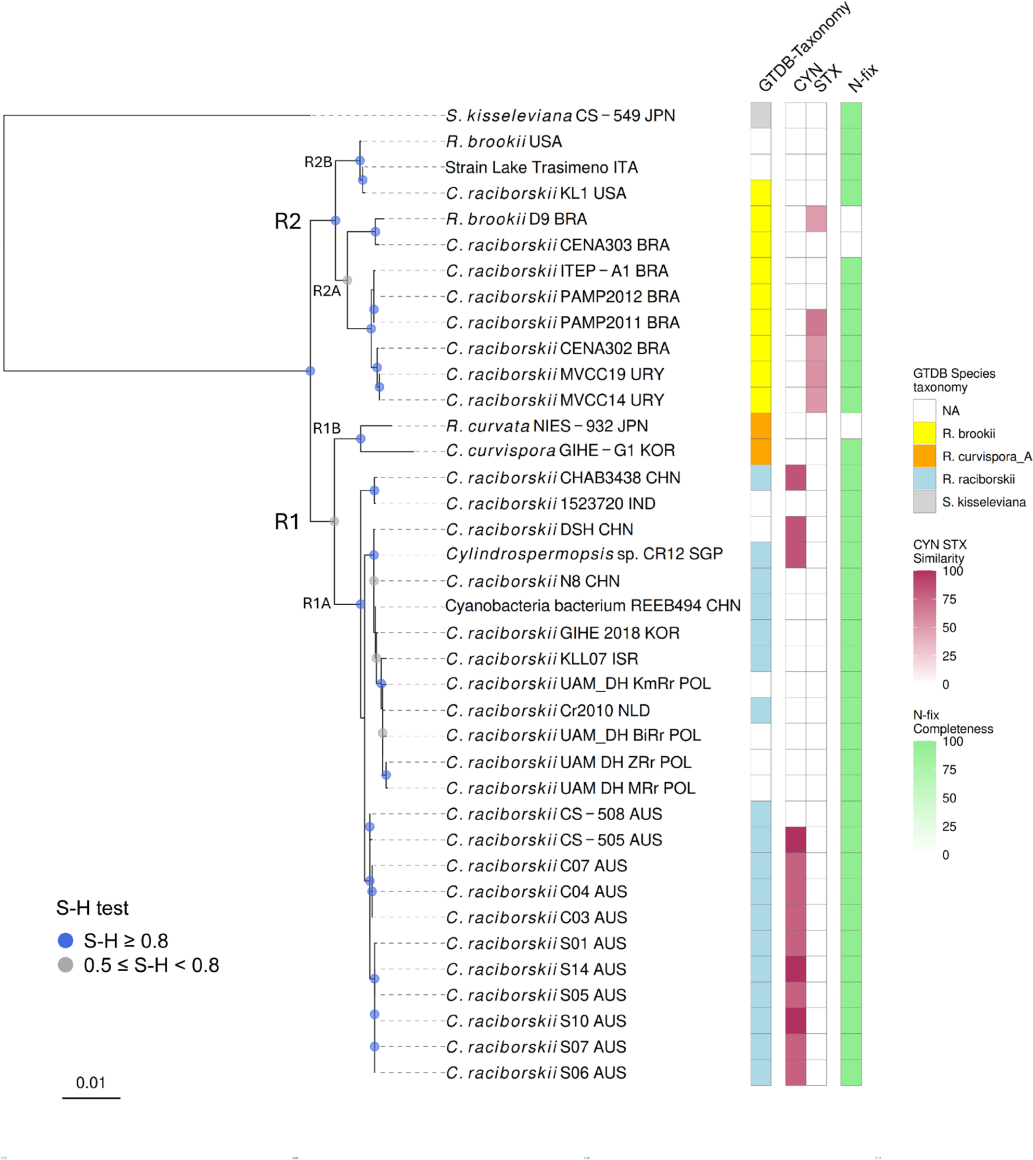
Phylogenomic tree of *Raphidiopsis*, including the strain isolated from Lake Trasimeno and a selection of genomes from the Genome Taxonomy Database (GTDB) and NCBI GenBank. Species names and strain identifiers in the tree tips are from the NCBI taxonomy; the last three letters in the taxa names indicate the geographic origin of the genomes (Table S1). The main clades in the tree are indicated with R1A‐B and R2A‐B. The GTDB taxonomy classification is given in the GTDB‐ Taxonomy colour code column. The colour code columns CYN and STX indicate the presence in the genomes of biosynthetic gene clusters (BGCs) encoding cylindrospermopsins and saxitoxins, respectively; colour gradients refer to the similarity of the identified BGCs to known clusters of genes encoding the two toxins estimated by antismash. The column N‐fix reports the presence of N‐fixing genes identified with the KEGG module analysis. The tree was rooted with *Sphaerospermopsis kisseleviana* CS‐549 as outgroup. S–H, Shimodaira–Hasegawa test computed by FastTree; high SH‐like local support values (e.g., ≥ 0.8) indicate strong statistical support for the branch. The scale bar indicates the number of substitutions per site.

The phylogenomic tree obtained from the GTDB‐Tk analysis clearly showed a separation of the two blocks of species identified as *R. raciborskii* and *R. brookii* in the GTDB taxonomy (R1 and R2 clades; Figure 1). The computations of the ANI_b_ between the genomes included in the clades R1 and R2 provided values between 0.916 and 0.936 (average 0.928). In the R1 clade, *R. curvata* and *R. curvispora* (‘*R. curvispora*_A’ in the GTDB taxonomy) formed a separate cluster (R1B; ANI_b_ = 0.959) from the main group of *R. raciborskii* species isolated from Australia, Asia and Central Europe (R1A; ANI_b_ between 0.976 and 0.999); the ANI_b_ values between the genomes included in the R1A and the R1B clades were between 0.946 and 0.959. The Lake Trasimeno strain formed a compact cluster with the two genomes identified in the USA (R2B; ANI_b_ between 0.995 and 0.997). At lower ANI_b_ values (R2A vs. R2B, 0.953–0.961), this group was part of the larger group of the GTDB *R. brookii* species isolated from South America (R2A; ANI_b_ between 0.964 and 0.999). The topology of the phylogenomic tree obtained with GTDB‐Tk was confirmed by that obtained using GtoTree (figure not shown).

The main separation in the *Raphidiopsis* tree (R1 and R2) showed correspondence in the presence of two different gene clusters encoding CYN and STX in a selected number of species in the R1A and R2A groups, respectively (Figure 1). No operons encoding legacy cyanotoxins were identified in the *R. curvispora/R. curvata* group and (besides the Lake Trasimeno strain), the two genomes from North America. In the R1A group, the presence of gene clusters encoding CYN was found only in the Australian and Asian isolates and not in the European and Israel strains. Nevertheless, the presence of short fragments (355–481 bp) identical (100%) to *cyrJ* and *cyrA* sequences was identified in the UAM/DH‐ZRr (POL) strains, while short fragments of sequences equivalent to the *cyrJ* gene (> 98%) were identified in the other three Polish genomes, and in the strains from Israel, Netherlands and in *C. curvispora* GIHE‐G1 (KOR). In the South America group (R2A), among the strains lacking the STX encoding operon, the strain ITEP‐A1 showed the presence of *sxtA* and *sxtI* genes. Among secondary metabolites, a gene cluster encoding hassallidin (antifungal lipopeptide) was identified in almost all the species, with exclusion of a few strains in China ( *C. raciborskii* CHAB3438_CHN) and South America (5 strains). Furthermore, an operon encoding rhizomide A/B/C (lipopeptides with antimicrobial and siderophore‐like activity) was identified in R1A strains (6 Australian and 1 Chinese) and in one R2A South American strain ( *C. raciborskii* CENA303). Save for a few other rare gene clusters present in single species, the arylpolyene (antioxidants) operon was identified (with a very low similarity, 8%–12%) in several strains in all the phylogenetic clades. Excluding an operon encoding puwainaphycin F/minutissamide A/B/C/D (30% similarity) in *R. raciborskii* KL1, the three R2B genomes shared a comparable secondary metabolite profile.

With the exclusion of *R. brookii* D9 and *C. raciborskii* CENA303 (R2A), and *R. curvata* NIES‐932 (R1B), the KEGG analysis allowed identification of the *nif* genes (KEGG module M00175) in all the analysed strains (Figure 1). With a very few, minor differences, the other pathway modules identified by the KEGG annotation of the Lake Trasimeno genome were the same as those obtained with the annotation of the other two R2B genomes, that is, *C. raciborskii* KL1 and *R. brookii* (Table S3). Overall, differences among all the strains were mostly limited to a few modules, generally with low completeness, such as Lipopolysaccharide metabolism, Biosynthesis of terpenoids and polyketides and Biosynthesis of other secondary metabolites (Table S3). Nevertheless, functional enrichment analyses contrasting the R1 and R2, and the R1A and R2A groups did not show any significant geographical pattern in the presence of the functional modules (adjusted *q* value > 0.4).

Similarly to the Lake Trasimeno strain, no ARGs were identified in any of the other *Raphidiopsis* strains considered in this work, with the sole exception of *R. brookii* D9, which showed the presence of a type A‐1 chloramphenicol O‐acetyltransferase.

### 3.7 Pangenomic Analysis

**FIGURE 2:**
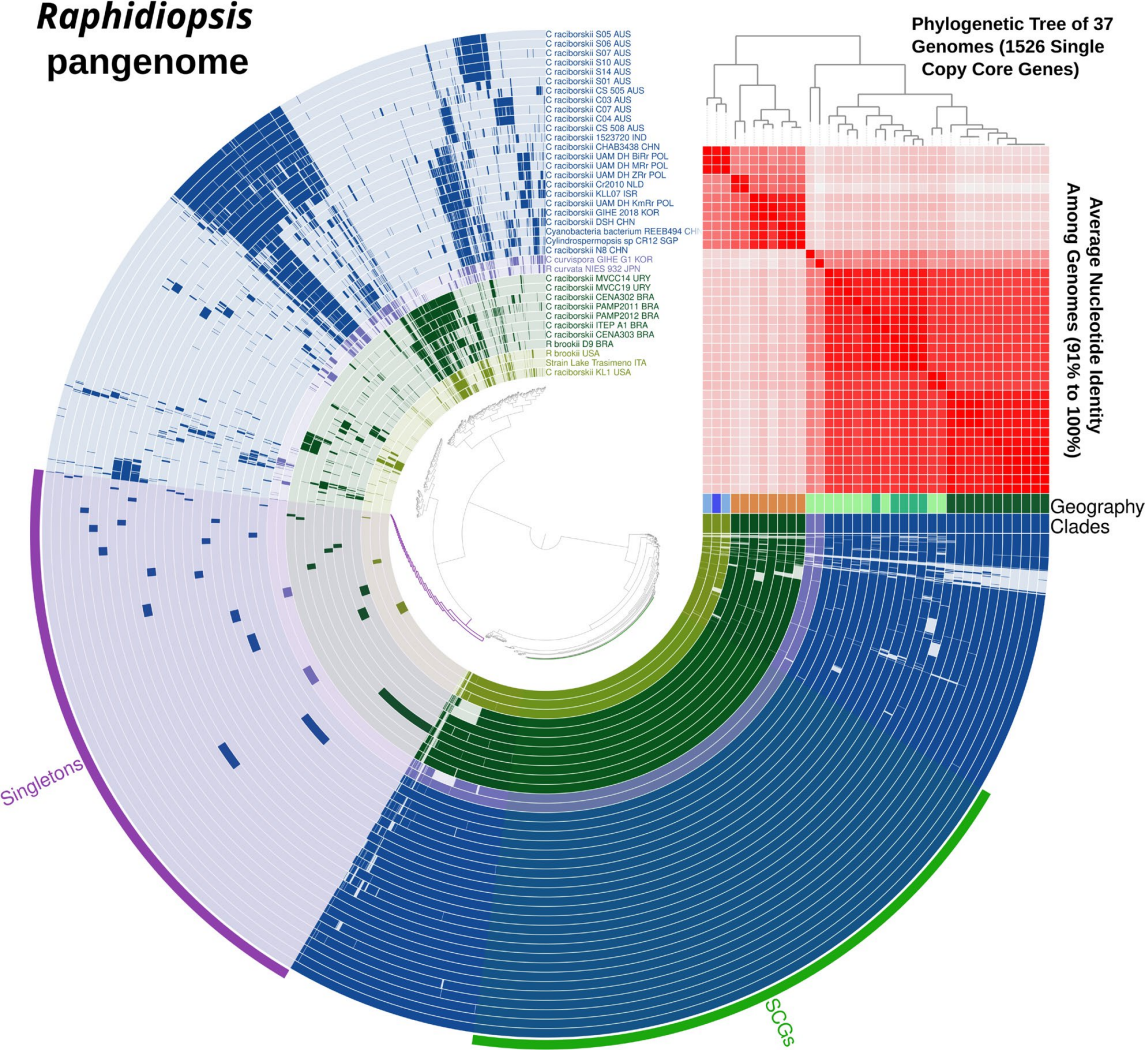
The pangenome of *Raphidiopsis*. Each one of the 6066 gene clusters is represented by a radius that contains one or more amino acid sequences that originate from one or more genomes, which are represented by the 37 circles. Gene clusters are organised according to their distribution across the genomes, so that gene clusters that occur together in the same group of genomes are closer together. Genomes are ordered consistently with the phylogenomic tree on the top right side of the figure, based on the single copy core genes (SCGs), as well as the symmetrical matrix reporting the ANI_b_ values computed between all 37 genomes. The outer circle highlights the two regions of the pangenome including the 1526 SCGs and the 1500 singletons. On the right‐hand side of the figure, Clades and Geography show the four main clades identified in the phylogenomic tree (Figure 1) and the indication of the geographical distribution of the genomes (as reported in detail by the last three code letters in the names of the taxa) respectively.

**FIGURE 3:**
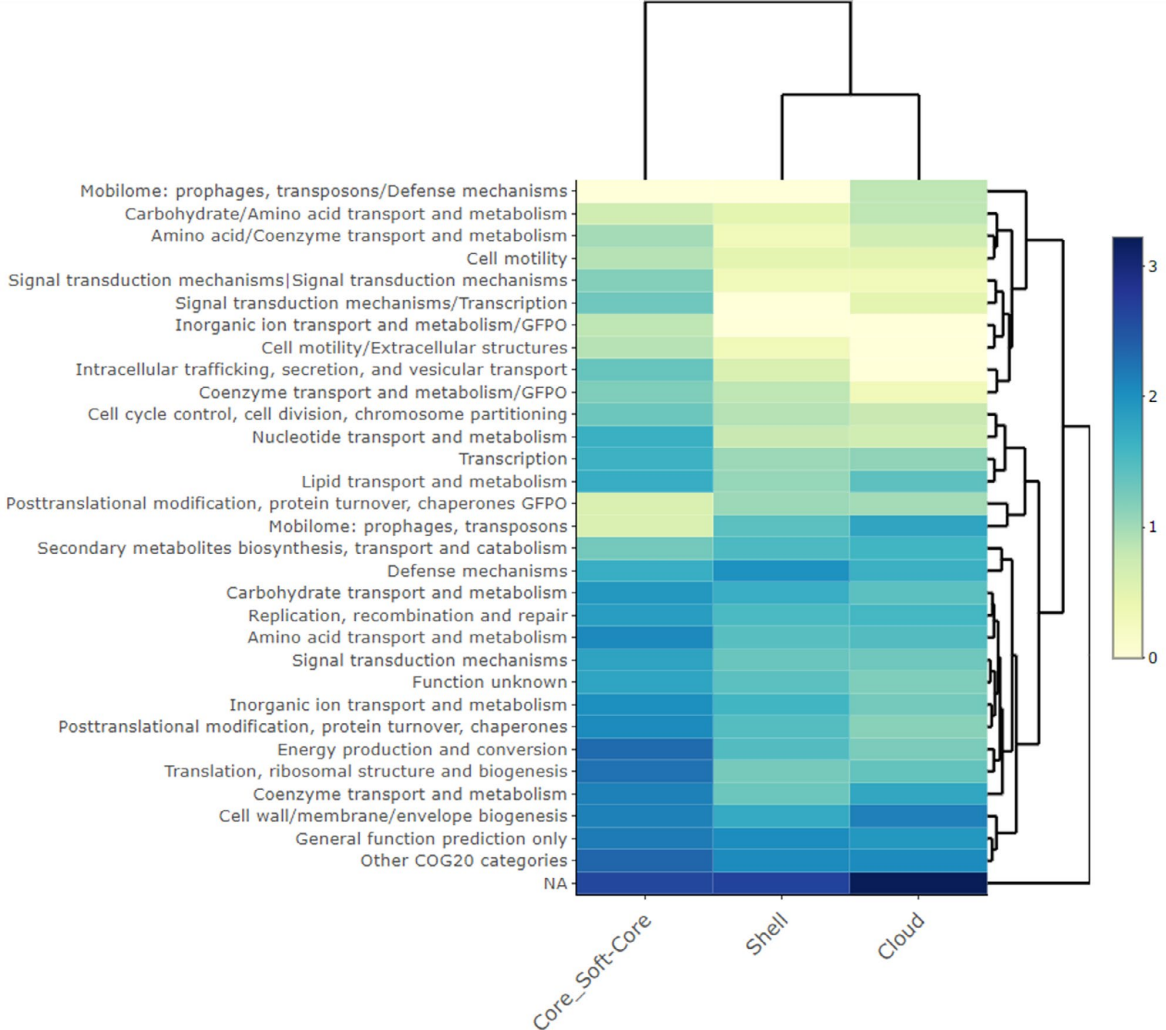
Heatmap showing the most abundant COG20 categories in the main pangenome groups, namely Core_Soft‐Core (grouping the Core and Soft‐Core), Shell and Cloud genomes. Less represented categories in genomes (< 6) were grouped under ‘Other COG20 categories’. GFPO, General Function Prediction Only. Before plotting, the data were transformed by log_10_(*x* + 1). Unclassified gene families were grouped under ‘NA’.

The pangenomic analysis of the 37 *Raphidiopsis* genomes identified a total of 126,318 genes grouped in 6066 gene clusters. The single‐copy core gene clusters (SCGs) and the singletons (genes present in a single genome) represented 25.2% and 24.7% of the total gene clusters, respectively (Figure 2). Overall, 60% of all clusters contained genes that were annotated with COG functions (COG20_FUNCTION). The functional annotation rate was 84% for the SCGs and 27% for the singletons. The analysis by panaroo identified 6800 ‘total genes’, with a distribution in the corresponding classes defined by panaroo (Core, Soft, Shell and Cloud) comparable with the results obtained by Anvi'o (Figure S2). The distribution of the gene families classified using the COG20 categories showed a prevalence of mobilome (prophages transposons), defence mechanisms, secondary metabolites biosynthesis, carbohydrate and amino acid transport metabolisms in the cloud and, partly, in the shell pangenome (Figure 3).

The phylogenomic analysis based on the 1526 SCGs (Figure 2, top right) grouped the 37 genomes consistently with the ANI_b_ values and the topology of the phylogenomic analysis by GTDB‐Tk based on a selection of 120 gene markers, as well as with the geographic distribution of *Raphidiopsis* strains (Figure 2; Figure S3). Furthermore, the grouping was also consistent with the differential pattern (genomic islands *sensu* Coleman et al. 2006) of shell and cloud gene clusters in the upper left of Figure 2.

Enrichment analysis allowed the identification of functions unique to the two main geographical groups R1 (R1A and R1B) and R2 (R2A and R2B), corresponding to genomes from Australia, Asia and Central Europe, and South/North America and central Italy, respectively (Table S4). Given that the test is not reliable when some groups have very few genomes (Shaiber et al. 2020), other comparisons (e.g., contrasting R2B, including the Lake Trasimeno genome) were not possible. Enrichment analysis revealed several differences, some within common categories with different specific functions in the two groups (defence mechanisms; translation, ribosomal structure and biogenesis), but the majority within different categories. Specifically, major differences between the two groups included functions in carbohydrate metabolism, metal resistance, lipid metabolism and secondary metabolite pathways (see Discussion).

The Heaps model applied to the whole pangenome gave gamma values higher than zero considering the gene clusters from both Anvi'o (*γ* = 0.20) and panaroo (*γ* = 0.21). Similar results were obtained considering the fitting of the model to the Anvi'o and panaroo outputs separately for the R1 (*γ* = 0.14 and *γ* = 0.16) and R2 clades (*γ* = 0.18 and *γ* = 0.14), respectively.

## 4 Discussion

The genomic analysis of a strain of *R. raciborskii* isolated from a surface sample collected in Lake Trasimeno allowed us to clarify for the first time the taxonomic position and functional characteristics of a species collected in a southern European region.

### 4.1 Main Features of the Lake Trasimeno Strain

After 30 years since the identification of *R. raciborskii* in Lake Trasimeno in 1995, the presence of this species has been progressively documented in many other Italian water bodies, representing an important constitutive species in meso‐eutrophic lakes (Austoni and Stefanelli 2024; Del Pasqua et al. 2024; Austoni et al. 2025).

From a water management perspective, the absence of legacy cyanotoxins in the environmental samples and the lack of operons encoding cyanotoxins further confirm the general absence of toxigenic strains in the European continent. The detection of CYN in central Italy (Manti et al. 2005; Messineo et al. 2010) or STX in Lake Comabbio (northern Italy) (Austoni and Stefanelli 2024) during the development of *R. raciborskii* was based on the analysis of environmental samples, which cannot exclude the presence of other CYN/STX‐producing species, highlighting the need to examine isolated strains or genome/MAGs for a consistent evaluation of (potential) toxin producers. However, these observations call for a more comprehensive survey focused on studying the cyanobacterial community in different seasons. This is important also considering the huge abundance that the populations of *R. raciborskii* can develop in Lake Trasimeno, that is well over 100,000 cell mL^−1^ (this work, and https://apps.arpa.umbria.it/). It is worth highlighting that the absence of legacy cyanotoxins does not necessarily imply an absence of toxicity (Fastner et al. 2003).

Genome annotation by KEGG identified the main modules associated with the physiology of photosynthetic cyanobacteria, and several other modules encoding specialised functions relevant to bloom‐forming nitrogen‐fixing heterocytous Nostocales (Cao et al. 2020). Besides N‐fixation, the LT_0923 genome included several modules involved in the scavenging and transport of macro‐ and micronutrients. In Lake Trasimeno, P, N and C can reach low critical concentrations during the bloom periods. This was apparent in the low concentrations of N and the high values of pH detected during the sampling. Besides potential N‐fixation (Natwora and Sheik 2021; Ehrenfels et al. 2023), the presence of several ABC cassette transporters for several organic compounds suggested an active role in the transport of N and other nutrients from the environment (Shvarev and Maldener 2018). At pH around 9, CO_2_ is almost absent (Stumm and Morgan 1996), and cells require specific transporters for carbon concentration (Maeda et al. 2000; Koropatkin et al. 2007).

The regulation of oxidative stress response from the production of ROS as a byproduct of photosynthesis was associated with a specific set of genes. Nevertheless, compared to cyanobacteria forming surface blooms and scums (Salmaso et al. 2024; Buzzi et al. 2025), this specific stress may be mitigated in species developing massively in a turbid water column.

### 4.2 Taxonomic Assessment

The ANI_b_ values (> 0.99) and the phylogenomic analysis based on the 120 GTDB‐Tk markers associated the Lake Trasimeno LT_0923 strain with the two published North American genomes. In turn, at lower values (ANI_b_ between 0.95 and 0.96), these three assemblies were associated with all the other genomes isolated from South America. Within the South American group, the strong association between *R. brookii* D9 and *R. raciborskii* CENA303 was also confirmed by the common inability to fix atmospheric nitrogen. In algal cultures, these two species did not produce heterocytes (Hill 1972; Abreu et al. 2018), showing attenuated (CENA303) and continuously attenuated towards ends (D9) trichomes. The presence of very attenuated apices and the absence of heterocytes were considered two essential diacritical features characterising the original description of the *R. brookii* species by Hill (1972); see also Komárek (2013). However, the presence of heterocytous strains (including Lake Trasimeno strain) within the R2A/R2 clade challenges the validity of these two original diacritical characters in supporting the definition of different species.

Besides the American and the unique Southern Europe genome (LT_0923), all the other genomes in the R1 group formed a separate cluster including strains from Australia, Asia and Central Europe. Besides the separation of the *R. curvata* /*R. curvispora* isolates (R1B), the other genomes in R1A showed a compact cluster (ANI_b_ > 0.976), but with a further secondary separation of the Australian clade from the Asian and Central European strains.

Overall, the ability to fix atmospheric nitrogen and the presence of heterocytes can be considered an ancestral and not distinguishing character of *Raphidiopsis*. As outlined by Aguilera et al. (2018), the lack of heterocytes would be a consequence of independent events of secondary loss occurring in different geographical areas. Conversely, the production of CYN and STX was circumscribed to the R1A and R2A clades, respectively, showing a strong geographical pattern. The presence of vestiges of *cyr* genes in non‐toxigenic producing European strains is consistent with the observation that CYN production is an ancestral trait (Hoff‐Risseti et al. 2013; Piccini et al. 2013).

The distinction of different *Raphidiopsis* populations and their correspondence with specific geographical locations is an intriguing observation, also considering that most of the culture isolates showed morphological diacritical features which were considered to be essentially consistent with the description of the species *R. raciborskii* as classified and/or described, for example, for the Australian (Fuentes‐Valdés et al. 2018; Willis et al. 2018), Asian (Chen et al. 2022), European (Tokodi et al. 2025), South American (Abreu et al. 2018) and North American (Martin et al. 2021) strains. Excluding the R. curvispora_A clade (R1B), while the main clusters R1A and R2 showed ANI_b_ values each congruent or partially congruent with the attribution of genomes to one unique species (ANI_b_ > 0.98 and ANI_b_ > 0.95, respectively), the ANI_b_ computed between the genomes in the two main clades R1 and R2 showed values between 0.92 and 0.94, that is, in the limbo of values between 0.90 and 0.95 defined by Rodriguez‐R et al. (2024) as representing a discontinuity/gap between the thresholds separating species.

A comparison of the main KEGG pathway modules allowed us to identify, with very few minor exceptions, a close correspondence of functions in the two North American strains and the Lake Trasimeno strain (R2B). On the other hand, an enrichment analysis contrasting the two main clades R1 and R2 did not show any significant difference in the modules identified in the two groups. This result is not surprising considering that the KEGG modules mostly include important conserved and general pathways, often representing core metabolic functions that are only differentiated in separate evolutionary groups. Considering the pangenomic analysis (next section), less than 10% and 33% of the gene families were annotated with the KEGG modules and Kofam codes, respectively.

### 4.3 Pangenomic Assessment

The number of gene families identified by the pangenomic analyses must be treated with caution, as they depend not only on the number and origin of the genomes analysed but also on the software and on the thresholds and coverage parameters for similarity sequences used to predict gene families (Tettelin et al. 2008; Vernikos et al. 2015; Costa et al. 2020). For example, by analysing a different set of 31 *Raphidiopsis* genomes, Laux et al. (2023) obtained a pangenome including 7943 protein clusters, with indications about the presence of an open pangenome.

The geographic and genomic groups defined by the phylogenomic analyses showed a close correspondence with the presence of groups of genes belonging to subgroups of genomes in the shell and cloud gene clusters, which further helped to distinguish genomes within the major groups. While the core genome generally includes genes necessary for the basic metabolic activities, the shell and cloud pangenomes include genes involved in the adaptation to specific environments, providing insights into the mechanisms and causes driving differentiation.

The open pangenome in *Raphidiopsis* is indicative of the existence of populations developing in diverse regions. Compared to closed pangenomes, open pangenomes are larger, have a smaller proportion of core genes, and have higher rates of gene acquisition by horizontal gene transfer (HGT) (Brockhurst et al. 2019). These characteristics align with populations developing in distant geographical regions, acquiring genes from other species or developing adaptations necessary for survival in different habitats. Conversely, closed pangenomes are typical of species growing in homogeneous environments or extreme habitats, which require highly specialised adaptations and conserved genetic characteristics such as in pathogenic and symbiotic species (Park et al. 2019).

The differential presence of COGs in the two main R1 and R2 clades may be indicative of different functional adaptations to local environmental pressures. The list of differential COGs and their discussion is reported in Table S4. Among others, development in different geographic regions can be reflected by the presence of distinctive defence mechanisms, exclusive enrichment of the mobilome associated with prophages and transposons, carbohydrate transport (linked to utilisation of external organic compounds), exclusive presence of metal efflux pumps linked to specific resistance to toxic metal ions, differential regulation of gene expression and maintenance of cell wall functionality.

As expected, differences may also manifest in genomes isolated from the same region. For example, the differences apparent in Figure 2 between the ‘straight’ S01–S14 and ‘coiled’ C03–C07 strains isolated from Lake Wivenhoe (Australia) were analysed in detail by Willis et al. (2018), who concluded that genomic variability exists among co‐occurring strains and may be the basis for strain phenotypic differences and population plasticity.

### 4.4 Geographical Distribution of *Raphidiopsis*

Among speciation processes, differentiation between bacterial populations may occur at the subspecies level within ecological niches (ecovars, ecological variants) and due to geographical separation (geovars, geographical variants) (Staley 2006). A well‐known example of ecovars is the different strains of *Prochlorococcus* adapted to different levels of light intensity (Delmont and Eren 2018; Ulloa et al. 2021), while several pieces of evidence have been accumulated on the differentiation between species (geovars) from different regions on Earth (Staley 2006; Choudoir et al. 2016). In all cases, HGT is pivotal in contributing to cyanobacterial differentiation, including transduction (Rai et al. 2021).

In this regard, the presence of genomic islands characterised by different physiological and toxigenic traits is consistent with the gain and loss of genetic traits after the geographical separation of populations. Within the shell and cloud genome, genomic islands are the results of the different histories of adaptation and changes mediated by HGT, contributing to expanding the accessory genome and differentiation of *R. raciborskii* in different geovars. The ANI differences between separated geographical groups in the limbo of values between 0.92 and 0.94 would suggest the existence of an active plastic pangenome and, possibly, a (continuing) separation into different geospecies (geographically distinct species).

The close evolutionary and functional connection of the Lake Trasimeno *R. raciborskii* strain with the North American assemblies is consistent with the results obtained in previous phylogenetic studies based on the analysis of 16S rRNA and housekeeping genes, which found a tight coupling between strains isolated from North America and strains isolated from Southern Europe, that is Spain (Cirés et al. 2014) and Greece (Panou et al. 2018). Considering also the close coupling found between Tunisian, Greek and USA isolates (Moreira et al. 2015; Panou et al. 2018), this work adds new geographic and genomic evidence for a close connection between North America and (part) of southern European populations, calling for new assumptions on the phylogeography and dispersal routes of *R. raciborskii*, and adding new evidence to a connection of the species between the north American continent and the Mediterranean area (Haande et al. 2008; Cirés et al. 2014; Panou et al. 2018), even though the transport mechanisms remain to be clarified (Sha et al. 2025).

### 4.5 Study Methodological Limitations

The results are strongly influenced by the limited number of genomes characterised to date and available in public repositories. The lack of *R. raciborskii* genome coverage in Africa and the few strains available in Europe, North America, and North Asia make it difficult to reach firm conclusions. The open pangenome is expected to grow as more genomes are included, in particular increasing the cloud and (to a lesser extent) reducing the core component (Cai et al. 2023). Greater geographic coverage of genomes will allow for a more robust understanding of taxonomy and fine‐scale changes and adaptations captured in the shell and cloud pangenomes, although reliable interpretations may still depend on the completeness of metabolic databases. Furthermore, potential limitations of full shotgun sequencing technologies (which result in broken assemblies and variable number of contigs, thus also affecting functional mapping; Eisenhofer et al. 2023) can be solved or mitigated by the adoption of new state‐of‐the‐art long‐read technologies that allow annotation of circular genomes (Driscoll et al. 2017). Finally, while functional genomic analyses indicate the presence and potential expression of genes, transcriptomic approaches are required to assess their expression (Choi et al. 2016).

## 5 Conclusion

The genomic analysis of the LT_0923 strain of *R. raciborskii* isolated from Lake Trasimeno allowed us to understand the taxonomic position and functional characteristics of a strain isolated in the Mediterranean region compared with other assemblies distributed worldwide. Phylogenomic analyses based on taxonomic markers indicated a strong connection between the Mediterranean strain and the corresponding strains from North America and South America, as well as a separation from the Australian, Asian, and Central European strains. The results were confirmed by the analysis of gene clusters in the core genome of the whole set of *Raphidiopsis* genomes, indicating conservation of the phylogenetic signal through vertical inheritance, but with a concomitant high gene flow confirmed by the high proportion of cloud genes. The new results confirmed the ancestral character of nitrogen fixation, which was lost only in a few strains. Conversely, the presence of STX and CYN did show a circumscribed distribution in the South American, and the Australian and Asian strains, respectively. Causes of the absence of toxicity in the North American/Mediterranean genomes (STX), and in the European and the majority of Asian strains can be due to several factors, including operon loss/changes (evidenced by cyanotoxins gene vestiges), differential HGT and genetic drift. The open pangenome, and the large fraction and geographically distinct gene families identified in the cloud genome, which is enriched with genes specialised in environmental‐specific functions and defence mechanisms, are consistent with the development of *Raphidiopsis* in geographically distinct regions. Though this work contributed to filling a knowledge gap concerning the taxonomy and functions of a *Raphidiopsis* strain isolated in the Mediterranean region, the overall knowledge of the taxonomy of this genus is far from being considered complete and strongly biased by the small number of genomes so far characterised.

## Author Contributions

**Nico Salmaso:** conceptualization, software, formal analysis, writing – original draft, writing – review and editing, investigation. **Leonardo Cerasino:** methodology, investigation, writing – review and editing. **Margherita Di Brizio:** methodology, investigation, writing – review and editing. **Massimo Pindo:** methodology, investigation, writing – review and editing. **Adriano Boscaini:** methodology, investigation, data curation, writing – review and editing.

## Ethics Statement

All prevailing local, national and international regulations and conventions, and normal scientific ethical practices, have been respected.

## Conflicts of Interest

The authors declare no conflicts of interest.

## Supporting information


Appendix S1.

**Appendix S2.** **Figure S1** (A) Location of Lake Trasimeno in central Italy and sampling sites at Castiglione del Lago (red circle) and Passignano sul Trasimeno (TRS30) and Magione (TRS35) (yellow circles) (Table 1). The *Raphidiopsis* Lake Trasimeno strain was collected at the Castiglione del Lago sampling site shown in the photo (September 12, 2023); the Polvese Island can be seen in the background. Complete physical and chemical analyses were performed on samples collected at Passignano sul Trasimeno and Magione by the Environmental Agency of the Umbria Region (September 11, 2023) (https://apps.arpa.umbria.it/acqua/qualita‐acque‐superficiali). (B) Two single filaments of *Raphidiopsis raciborskii* developed in culture conditions from the isolation of an individual from the sample collected at Castiglione del Lago; heterocytes are visible at the end of the filaments; yellow scale bars, 5 μm.
**Figure S2.** Presence of each gene family in the 37 genomes analysed in this work based on the anvio and panaroo analyses. The classification scheme used is that proposed by panaroo. Core (99% ≤ strains ≤ 100%), Soft core (95% ≤ strains < 99%), Shell (15% ≤ strains < 95%) and Cloud (0% ≤ strains < 15%).
**Figure S3.** (A) Phylogenomic tree of *Raphidiopsis* based on the 1526 single‐copy core gene clusters (SCGs) identified in the pangenomic analysis; the tree was rooted using midpoint rooting; UFBoot, Ultrafast bootstrap values. Other details as in Figure 1. (B) Tanglegram comparing the topology of the two phylogenomic trees obtained by the gtdbtk analysis of Figure 1 (left) and the analysis in the panel (A) based on the 1526 SCGs (right); where relevant, branches were rotated around the nodes to maximise the correspondence between the tips.

## Data Availability

This Whole Genome Shotgun project has been deposited at DDBJ/ENA/GenBank under the accession JBMNDW000000000. The version described in this paper is version JBMNDW010000000. The sequences are publicly available under BioProject accession number PRJNA1215010, BioSample accession number SAMN46392051, and SRA accession number SRR32208149. The accession number for the 16S rRNA gene obtained from Sanger sequencing is PV054108.
