# Supplementary material for: Phylogenomic and Pangenomic Assessment of a Mediterranean Strain of Raphidiopsis raciborskii Extends Knowledge of the Global Distribution and Characteristics of a Potentially Toxigenic Cyanobacterium
Source: Environ Microbiol Rep. 2025 May 19;17(3):e70098. doi: 10.1111/1758-2229.70098 (PMC12089652; doi:10.1111/1758-2229.70098)
Supplement: Supplementary file 1 — Appendix S1. [file EMI4-17-e70098-s001.pdf]

Phylogenomic and pangenomic assessment of a Mediterranean strain of *Raphidiopsis raciborskii* extends knowledge of the global distribution and characteristics of a potentially toxigenic cyanobacterium

Nico Salmaso, Leonardo Cerasino, Margherita Di Brizio, Massimo Pindo, Adriano Boscaini

Supplementary Tables

Table S1

Species belonging to the *Raphidiopsis* species analysed in this work. NCBI assembly codes: GenBank assembly accession codes. NCBI organism name: the taxon name and strain code in the NCBI GenBank taxonomy. GTDB organism name: the classification adopted in the GTDB (Genome Taxonomy Database) taxonomy. Country code: alpha-3 country codes indicating where the organism has been collected.

**NCBI assembly code**	**NCBI Organism Name**	**GTDB Organism name**	**Country code**
GCA_002893155.1	*Cylindrospermopsis raciborskii* C03	Raphidiopsis raciborskii	AUS
GCA_002893145.1	*Cylindrospermopsis raciborskii* C04	Raphidiopsis raciborskii	AUS
GCA_002893125.1	*Cylindrospermopsis raciborskii* C07	Raphidiopsis raciborskii	AUS
GCA_001676585.1	*Cylindrospermopsis raciborskii* CS-505	Raphidiopsis raciborskii	AUS
GCA_001858115.1	*Cylindrospermopsis raciborskii* CS-508	Raphidiopsis raciborskii	AUS
GCA_002893285.1	*Cylindrospermopsis raciborskii* S01	Raphidiopsis raciborskii	AUS
GCA_002893265.1	*Cylindrospermopsis raciborskii* S05	Raphidiopsis raciborskii	AUS
GCA_002893245.1	*Cylindrospermopsis raciborskii* S06	Raphidiopsis raciborskii	AUS
GCA_002893205.1	*Cylindrospermopsis raciborskii* S07	Raphidiopsis raciborskii	AUS
GCA_002893215.1	*Cylindrospermopsis raciborskii* S10	Raphidiopsis raciborskii	AUS
GCA_002893185.1	*Cylindrospermopsis raciborskii* S14	Raphidiopsis raciborskii	AUS
GCA_002027345.1	*Cylindrospermopsis raciborskii* CENA302	Raphidiopsis brookii	BRA
GCA_002114155.1	*Cylindrospermopsis raciborskii* CENA303	Raphidiopsis brookii	BRA
GCA_001586755.1	*Cylindrospermopsis raciborskii* ITEP-A1	Raphidiopsis brookii	BRA
GCA_026929835.1	*Cylindrospermopsis raciborskii* PAMP2011	Raphidiopsis brookii	BRA
GCA_026929755.1	*Cylindrospermopsis raciborskii* PAMP2012	Raphidiopsis brookii	BRA
GCA_000175855.1	*Raphidiopsis brookii* D9	Raphidiopsis brookii	BRA
GCA_018969285.1	Cyanobacteria bacterium REEB494	Raphidiopsis raciborskii	CHN
GCA_022496815.1	*Cylindrospermopsis raciborskii* CHAB3438	Raphidiopsis raciborskii	CHN
GCA_036409495.1	*Cylindrospermopsis raciborskii* DSH	NA	CHN
GCA_018139025.1	*Cylindrospermopsis raciborskii* N8	Raphidiopsis raciborskii	CHN
GCA_035282025.1	*Cylindrospermopsis raciborskii* 1523720	NA	IND
GCA_021650815.1	*Cylindrospermopsis raciborskii* KLL07	Raphidiopsis raciborskii	ISR
GCA_002368135.1	*Raphidiopsis curvata* NIES-932	Raphidiopsis curvispora_A	JPN
GCA_014489415.1	*Cylindrospermopsis curvispora* GIHE-G1	Raphidiopsis curvispora_A	KOR
GCA_006523545.1	*Cylindrospermopsis raciborskii* GIHE 2018	Raphidiopsis raciborskii	KOR
GCA_003367075.2	*Cylindrospermopsis raciborskii* Cr2010	Raphidiopsis raciborskii	NLD
GCA_045377515.1	*Cylindrospermopsis raciborskii* UAM/DH-BiRr	NA	POL
GCA_045377475.1	*Cylindrospermopsis raciborskii* UAM/DH-KmRr	NA	POL
GCA_045377415.1	*Cylindrospermopsis raciborskii* UAM/DH-MRr	NA	POL
GCA_045377345.1	*Cylindrospermopsis raciborskii* UAM/DH-ZRr	NA	POL
GCA_001432185.1	*Cylindrospermopsis* sp. CR12	Raphidiopsis raciborskii	SGP
GCA_001858125.1	*Cylindrospermopsis raciborskii* MVCC14	Raphidiopsis brookii	URY
GCA_012583295.1	*Cylindrospermopsis raciborskii* MVCC19	Raphidiopsis brookii	URY
GCA_015708265.1	*Cylindrospermopsis raciborskii* KL1	Raphidiopsis brookii	USA
GCA_044352285.1	*Raphidiopsis brookii*	NA	USA

Table S2

(A) Blast analysis of rRNA genes and selected housekeeping genes of taxonomic relevance extracted from (B) the Lake Trasimeno genome annotated by PGAP and bakta, and (C) from the assembled unbinned contigs (Megablast; Max target sequences=500). Q. Len., query length; QC, query cover; pident, percentage identity; Acc. Len., accession length ("genome" if the subject sequence was included in a genome). The rRNA and a few of the selected housekeeping genes (core_nt, in bold) have been used in several phylogenetic analyses based on target genes (see the section Introduction).

(A)

Database: Core nucleotide database (core_nt)

Gene	Q. Len. (bp)	QC (%)	pident (%)	Acc. Len. (bp)	Blast top pident accession	NCBI Taxonomy	Water body
5S rRNA	118	100	100	genomes	several	*C. raciborskii/curvispora*	global
5S rRNA	114	100	100	genomes	several	*C. raciborskii/curvispora*	global
**16S rRNA**	120	100	100	genomes	several	*C. raciborskii/curvispora*	global
23S rRNA	2821	99	99.4	2818	LC455654.1	*C. raciborskii* NIES-1260	Gonoike Pond, JPN
rpoB	3342	100	96.92	genomes	CP073250.1	*C. raciborskii* N8	Zhenhai Reservoir CHN
rbcX	402	81	97.26	582	several	*C. raciborskii/R. mediterranea/R. curvata*	several, Asia
nifH	888	33-38	100	297-345	several	*C. raciborskii*/Uncultured bacterium	ESP, GRC, USA, MEX
rpoC1 (1)	1878	20	99.85	380	HG942518.1/19.1	*C. raciborskii* MM67 and MM69	Lake Whangape, NZL
cpcA	489	62	100	710	GU434457.1	*Cylindrospermopsis* WI04094B	Lake Wingra, USA
cpcB	522	43	100	611	JN903642.1	*C. raciborskii* UAM520	Vega del Jabalon ESP
**16S rRNA (unbinned)**	1485	98	100	1461	FM177492.1	*C. raciborskii* 1LT32S01	Lake Trasimeno ITA

(1) QC 35% (accession length=660), pident=99.85%, *C. raciborskii* AZ60, Fish Pond, Arizona, USA.

Database: Whole Genome shotgun contigs (wgs)

Gene	Q. Len. (bp)	QC (%)	pident (%)	Acc. Len. (bp)	Blast top pident accession	NCBI Taxonomy	Water body
5S rRNA	118	100	100	several	several	*C. raciborskii/curvispora*	global
5S rRNA	114	100	100	several	several	*C. raciborskii/curvispora*	global
16S rRNA	120	100	100	several	several	*C. raciborskii/curvispora*	global
23S rRNA	2821	100	99.75	4749	VOIM01000076.1	*C. raciborskii* LB2897	Lemon Lake, USA
rpoB	3342	100	99.97	24858	JBILEC010000040.1	*R. brookii* ME2016-07-27	Lake Mendota, USA
rbcX (1)	402	100	100	several	several	*C. raciborskii / R. brookii*	Four water bodies, USA
nifH (2)	888	100	100	82151	JBILEC010000040.1	*R. brookii* ME2016-07-27	Lake Mendota, USA
				259219	JADQCS010000002.1	*C. raciborskii* KL1	Kissena Lake, USA
rpoC1	1878	100	99.79	24858	JBILEC010000040.1	*R. brookii* ME2016-07-27	Lake Mendota, USA
				268160	JADQCS010000001.1	*C. raciborskii* KL1	Kissena Lake, USA
cpcA	489	100	100	61472	JBILEC010000007.1	*R. brookii* ME2016-07-27	Lake Mendota, USA
				2959	JBEAWA010000478.1	Cyan.NASQAN2011_262_B_bin.3	River water, USA
cpcB	522	100	100	64487	VOIM01000172.1	*C. raciborskii* LB2897	Lemon Lake, USA
16S rRNA (unbinned)	1485	100	99.87	4749	VOIM01000076.1	*C. raciborskii* LB2897	Lemon Lake, USA

(B)

Lake Trasimeno genome

5S ribosomal RNA

```
>5S ribosomal RNA TARS_fil_Rr_000000000265:2987-3105
CCTGGTGTCTATGGTGCGGTGGAACCACACTGATCCCTTCCCGAACTCAGAGGTGAAACGCTGTTGCGGCGACGATAGTA
TGGGGGTTGCCCTATGTCAAAATAGCTCGATGCCAGGT
```

```
>5S TARS_fil_Rr_000000002920:28692-28806 (partial=true)
ACCTGGCATCGAGCTATTTTGACATAGGGCAACCCCCATACTATCGTCGCCGCAACAGCGTTTCACCTCTGAGTTCGGGA
AGGGATCAGTGTGGTTCCACCGCACCATAGACAC
```

16S ribosomal RNA

```
>16S ribosomal RNA (partial) TARS_fil_Rr_000000001183:3039-3159 (partial=true)
AAAACGGAGAGTTTGATCCTGGCTCAGGATGAACGCTGGCGGTATGCTTAACACATGCAAGTCGAACGGGATGCTTAGGC
ATCTAGTGGCGGACGGGTGAGTAACGCGTGAGAATCTGGC
```

23S ribosomal RNA

```
>23S ribosomal RNA TARS_fil_Rr_000000000265:88-2909
GGTCAAGCTAATAAGGGCTCATGGTGGATACCTAGGCACACAGAGGCGAAGAAGGACGTGGTTACCTGCGAAAAGTTCCG
```

```
GGGAGTTGGAAGCAAACAGAGAGCCGGAAATATCCGAATGGGGCAACCCTGAACACTGCCTGTTGAATATATAGACAGGA
AAGAGCGAACCTGGCGAATTGAAACATCTTAGTAGCCAGAGGAAAAGAAATCAAAAGAGATTCCCCGAGTAGTGGTGAGC
GAAAGGGGAAAAGCCTAAACCAGGGGGTATACCTTCTGGGGTAGTGGGACAGCAAAATCGAATCTAGAGACTAGATGAAG
CAGCTAAATACTGCACCAGAGGGGGTGAAAGTCCCGTAGTCGAAAGTTGAAGGATAGAAGCTGAATCCCGAGTAGTACGG
GGCACGAGGAATCCCGTATGAATCAGCGAGGACCATCTCGTAAGGCTAAATACTACTGTGTGACCGATAGAGAACAAGTA
CCGCGAGGGAAAGGTGAAAAGAACCCCGGGAGGGGAGTGAAAAAGAACATGAAACCGTGAGCTTACAAGCAGTGGGAGTC
CGATTAAACGGATGACCGCGTGCCTGTTGAAGAATGAGCCGGCGACTTATAGGTACTGGTAGGTTAAAGCGGGAATGCTG
GAGCCAAAGGGAAACCGAGTCTGAAAAGGGCGATAATCAGTATTTATAGACCCGAACCCTGGTGATCTAACCATGGCCAG
GATGAAGCTTGGGTAAAACCAAGTGGAGGTCCGAACCGACCGATGTTGAAAAATCGGCGGATGAGCGGTGGTTAGGGGTG
AAATGCCAATCGAACCAGGAGCTAGCTGGTTCTCCCCGAAATGTGTTGAGGCGCAGCGGTAATGAAAAAATTGGGGGGTA
AAGCACTGTTTCGGTGCGGGCTGGGAGACCGGTACCAAATCGAGGCAAACTAAGAATACCCAAGGAGCACATTGCCAGTG
AGACGGTGGGGGATAAGCTTCATCGTCAAGAGGGAAACAGCCCAGACCACCAGCTAAGGTCCCCAAATCATCACTAAGTG
AAAAAGGAGGTGGGGTTGCAAAGACAACTAGGAGGTTTGCCTAGAAGCAGCCACCCTTGAAAGAGTGCGTAATAGCTCAC
TAGTCAAGCGATCCTGCGCCGAAAATGAAAGGGGCTAAGTGATGTACCGAAGCTGTGGGATTAGAAATAATCGGTAGGGG
AGCGTTCCGTAGTAGTGAGAAGCAGTAGCGGCAAGCAGCTGTGGACGAGACGGAAGTGAGAATGTCGGCTTGAGTAGCGC
AAACATTGGTGAGAATCCAATGCCCCGAAACCCTAAGGGTTCCAGAGGCAGGTTCGTCCACTCTGGGTTAGTCGGGACCT
AAGGCGAGGTCGAAAGGCGTAGTCGATGGAGACCGGGTCAACAATCCCGGACTACAATATGGGAGCAGAACTAGGGACGC
ATGAAAGATAGCCACGCCCTGAATGGATTGGGAAGACCGTTACGACGGTCGAGTGGGGAAGGAAAGTGCCAAGAAAAGCT
AGGGTTGTGATGAACATATAGTACCCGTACCCGAAACCGACACAGGTAGGGAGGTTGAGAATACCAAGGGGCGCGAGATA
ACTCTCTCTAAGGAACTCGGCAAAATGGCCCCGTAACTTAGGAAGAAGGGGTGCCAGCCGAAAGGCTGGTCGCAGTGAAG
AGATCCAGGCGACTGTTTACCAAAAACACAGGTCTCCGCTAACTCGAAAGAGGACGTATGGGGGCTGACGCCTGCCCAGT
GCCGGAAGGTTAAGGAAGTTGGTCAGTGGAAACATAAAGCTGACGACCGAAGCCCCGGTGAACGGCGGCCGTAACTATAA
CGGTCCTAAGGTAGCGAAATTCCTTGTCGGGTAAGTTCCGACCCGCACGAAAGGCGTAACGATCTGGATGGTGTCTCAGA
GAGAGACTCGGCGAAATAGGAATGTCTGTGAAGATACGGACTACCTGCACCTGGACAGAAAGACCCTATGAAGCTTTACT
GTAGCCTGGAATTGTGTTCGGGCTTGGCTTGCGCAGGATAGGTGGGAAGCGAAGAACTTCTCCTTGAGGGGGGAAGGGAG
CTAACGGTGAGATACCACTCTGGCGAAGCTAGAATTCTAACTCGTCACCGTAAGCCGGTGAGAGGAAAGTTTCAGGTGGG
CAGTTTGACTGGGGCGGTCGCCTCCTAAAAGGTAACGGAGGCGCGCAAAGGTTCTCTCAGCACGCTTGGAAACCGTGCGA
CGAGTGTAAAGGCAAGAAGAGAGCTTGACTGCAAGACCAACAAGTCGAGCAGGGACGAAAGTCGGCCTTAGTGATCCGAC
GGCGCAGCATGGAATGGCCGTCGCTCAACGGATAAAAGTTACTCTAGGGATAACAGGCTGATCTCCCCCAAGAGTCCACA
TCGACGGGGAGGTTTGGCACCTCGATGTCGGCTCATCGCAACCTGGGGCGGAAGTACGTCCCAAGGGTTGGGCTGTTCGC
CCATTAAAGCGGTACGTGAGCTGGGTTCAGAACGTCGTGAGACAGTTCGGTCCATATCCGGTGCAGGCGAAAGAACATTG
AGAGGAGTCCTCCTTAGTACGAGAGGACCGGGAGGAACGAACCGCTGGTGTACCAGTTATTCCGCCAGGAGTAGACGCTG
GGTAGCCAAGTTCGGAGAGGATAACCGCTGAAAGCATCTAAGTGGGAAGCCCACCTTAAGATGAGTGTTCTCACTACGAG
AGTAGGTAAGGTCACGGGGAGAAGACCCGTTGATAGGCTTTATGTGGAAGTACAGTAATGTATGTAGCAGAGGAGTCCTA
ACAGACCGAGGGCTTGACCTC
```

rpoB

```
>rpoB TARS_fil_Rr_000000002247:223036-226378
ATGAACAACGAAAATTACACGGAACCATCTTTCCTATTACCAGATCTAATTGAAATTCAGCGCTCTAGCTTTCGCTGGTT
TTTAGAAGAGGGTTTAATAGAAGAGCTGAACTCATTTAGTCCTATTACGGACTATACGGGAAAATTAGAACTCCATTTTT
TAGGACATAACTATAAGCTAAAAGAGCCGAAATATAGCGTAGAAGAGTCAAAAAGAAGAGATAGCACATACGGTGTACAG
ATGTATGTACCCACGAGATTGCTTAATAAAGAAACAGGTGATATTAAAGAACAGGAAGTATTTATAGGTGATCTACCGTT
AATGACGGATAGAGGAACCTTTATTATTAACGGAGCCGAGCGAGTAATAGTCAATCAAATCGTGCGATCGCCCGGAGTAT
ATTACAAATCAGAGATTGATAAAAACGGGAGAAGAACATATTCTGCGAGTCTGATTCCCAACCGGGGGGCATGGCTAAAA
TTTGAGACAGACCGTAATGATTTAGTGTGGGTAAGAATAGATAAGACCCGCAAATTGTCAATTCAGGTACTACTTAAAGC
ATTAGGACTGTCAGATAACGAAATTTTAGATGCTTTAAGGCATCCAGAATACTTTCAAAAGACCATTGAAAAAGAGGGAC
AATTCTCCGAAGAGGAGGCCCTTCTAGAGCTGTATAGAAAGCTGAGGCCCGGGGAACCACCCACGGTGATGGGGGGACAA
CAGCTATTAGAATCGCGCTTTTTCGATCCCAAACGTTACGACCTAGGTAGGGTAGGTAGATATAAACTTAACAAGAAACT
ACGCCTTTCCGTTCCTGACACCACTAGGGTTCTCACCCCAGGGGATATCCTATCCGCTGTGGACTATCTCATCAACCTAG
AATACGACATTGGTAGTATTGATGACATTGATCATTTGGGAAATCGCCGAGTTAGAAGCGTTGGTGAACTGCTTCAGAAC
CAAGTAAGAGTTGGTTTAAATCGTTTAGAGAGGATCATTAGAGAAAGAATGACCGTCTCCGATGCCGAAGTGTTAACACC
AGCATCACTAGTGAACCCCAAACCCCTAGTAGCAGCCATAAAAGAGTTTTTTGGTTCTAGTCAGCTAAGTCAATTCATGG
ATCAGACCAACCCCTTAGCGGAACTAACCCATAAACGACGCTTGAGTGCTTTAGGTCCTGGGGGACTAACCAGAGAGAGA
GCTGGTTTTGCCGTGCGAGACATTCATCCCAGCCACTACGGACGCATTTGCCCTATTGAAACGCCGGAAGGTCCAAATGC
GGGATTAATTGGTTCTCTAGCTACCCATGCTCGTGTTAACCAGTATGGATTTTTAGAAACACCCTTTAGACCCGTAGAAA
ACGGAAGAGTTTGCTACGAAAAACCTGCTGTTTACATGACAGCAGACGAGGAAGATGACCTGCGAGTTGCACCTGGTGAC
ATTCCCGTAGATGACAACGGACAAATACTAGGAATCCAAGTACCCGTTCGTTATCGCCAGGAATTTTCCACCACCACTCC
CGAACAGGTAGACTACGTAGCTGTGTCACCAGTACAAATTGTCTCAGTAGCCACCAGCATGATTCCCTTTTTGGAGCATG
ATGATGCCAACCGAGCCTTAATGGGTTCTAACATGCAAAGGCAAGCAGTACCCTTATTGAAACCCGAGCGTCCCCTAGTG
GGAACTGGACTGGAGGCCCAAGGTGCCAGAGATTCAGGTATGGTAATTGTTTCCCGTACTGATGGAGATGTGGTATATGT
AGATGCGACCGAGATTCGTGTTCGAGTTAAAGAAAAGGCCGCCCTTACCAACCGTGAAAGCGAAACTATTCCCCATAAAC
CCCAGGAAGTTAAATACGTTCTCTCCAAATATCAACGTTCTAACCAGGACACCTGTCTCAATCAAAAACCCCTAGTGCGG
ATTGGGGAAAAGGTCATAGCAGGTCAGGTATTAGCAGATGGTTCATCCACAGAAGGGGGAGAATTAGCATTAGGGCAAAA
TGTTATTGTTGCCTATATGCCCTGGGAAGGGTATAACTACGAAGATGCAATTTTAATTTCCGAGAGACTGGTACAAGAGG
ATGTTTACACATCAATACACATTGAAAAATTTGAAATAGAAGCAAGACAAACCAAACTAGGACCTGAAGAGATTACCAGA
GAAATACCCAACGTTGGGGAAGACGCCCTCAGACAATTAGACGAACAGGGGATAATCAGAGTAGGGGCCTGGGTAGAATC
AGGAGACATTCTAGTGGGGAAAGTGACCCCTAAAGGAGAATCAGATCAACCACCGGAAGAAAAACTACTCAGAGCGATTT
TCGGGGAGAAAGCTCGCGACGTGAGGGATAACTCCCTGCGGGTTCCCAATGGTGAAAAAGGAAGAGTAGTGGATGTGCGA
CTATTTACTAGGGAGCAGGGGGATGAACTTCCACCGGGAGCCAATATGGTGGTGCGGGTGTATGTAGCCCAGAAACGTAA
AATCCAGGTTGGTGACAAAATGGCTGGGAGACATGGAAATAAAGGCATCATTTCCCGAATTTTGCCCTTAGAAGACATGC
CATACCTTCCCGATGGCACCCCAGTAGATATAGTCCTTAACCCCCTAGGTGTACCTAGTCGAATGAACGTAGGACAGGTG
TTTGAATGCTTATTGGGTTGGGCGGGTCACAACCTGGGGGTACGCTTTAAAATCACCCCCTTTGATGAAATGTACGGGGA
AGAATCCTCCCGACGTATAGTTCACGGCAAATTATGGGAAGCTAGGGAAGAAACCAGTAGGGATTGGGTATATAATCCCG
AAAACCCTGGCAAAATCATGGTTTATGATGGGCGCACGGGTGAACCCTTTGATCGTGCAGTCACCGTGGGGATAGCCTAT
ATGCTTAAACTTGTCCATTTAGTTGATGATAAAATTCATGCTCGCTCCACAGGTCCTTATTCCTTAGTCACCCAGCAACC
CTTGGGTGGTAAAGCACAACAGGGAGGTCAAAGATTTGGAGAGATGGAAGTGTGGGCACTAGAGGCGTTTGGTGCTGCTT
ATACATTACAGGAACTGCTAACTGTGAAATCAGATGATATGCAGGGACGTAATGAAGCATTGAACGCCATTGTTAAAGGA
AAAGCTATTCCTCGTCCTGGCACTCCTGAGTCCTTCAAAGTGTTAATGAGAGAATTACAATCTTTAGGATTGGATATTGC
```

GGTACATAAAGTAGAAACCCAAACAGATGGTAGTTCTCTAGATGTAGAAGTTGACTTGATGGCCGATCAAGTATCTCGTC
GCACTCCTCCCCGTCCAACATACGAATCCTTTTCTCGTGATTCCCTGGATGAGGATGAGTAA

rpoC1

>rpoC1 TARS_fil_Rr_000000002247:226492-228370
ATGCGCTCCGTTCAATCTAATCAATTTGATTACGTCAAAATTGGCATTGCTTCACCAGAACGTATCCGCGCTTGGGGGA
ACGTACCTTACCTAATGGTCAGGTGGTTGGTGAGGTGACAAAACCGGAAACCATTAACTACCGCACCCTCAAACCAGAAA
TGGATGGTTTGTTTTGCGAGCGGATCTTTGGCCCTGCTAAAGACTGGGAATGTCACTGTGGTAAATATAAACGGGTTCGT
CATAGAGGTATTGTCTGTGAGCGCTGTGGGGTAGAGGTAACGGAATCACGAGTTCGCCGTCATAGAATGGGTTTCATTAA
GTTAGCAGCACCTGTAGCTCATGTTTGGTACCTCAAAGGCATTCCCAGTTATATTGCTATACTATTAGACATGCCCCTAC
GAGATGTTGAGCAAATCGTCTACTTTAACTCCTATGTAGTTTTAGCTCCTGGTAATGCCGATACACTCGTTTACAAGCAA
TTATTAACAGAAGACCAATGGTTAGAAATTGAGGATAGGATTTATAGTGAAGATTCTCAATTAGTAGGCGTAGAAGTGGG
AATTGGTGCCGAAGCTTTACTACGTTTGTTATCAGATATTAATTTAGAAGAAGAAGCAGAAAAACTGCGGGGGGAAATTG
AAGCAGCAAAGGGACAAAAACGGGCCAAATTGATTAAACGTCTGCGGGTAATTGACAATTTCATTGCCACTGGTTCCCAA
CCGGAATGGATGGTGATGTCAGCCATTCCAGTCATTCCACCCGATCTCCGTCCCATGGTACAACTAGACGGTGGTAGATT
TGCTACTAGCGATTTAAACGACCTCTACCGTCGGGTAATCAATCGCAATAATCGTTTAGCTAGACTACAGGAAATCCTGG
CGCCAGAAATCATTGTTCGCAATGAAAAACGCATGTTACAGGAAGCGGTAGATGCTTTAATTGACAATGGTCGGAGAGGA
CGCACCGTAGTAGGAGCCAATAACCGCCCATTAAAGTCCCTATCAGATATCATAGAAGGTAAACAGGGGCGATTTCGGCA
AAACCTCCTGGGTAAAAGGGTAGACTACTCAGGACGTTCGGTTATTGTAGTTGGTCCCAAATTGCAAATTCACCAGTGTG
GACTCCCCAGGGAGATGGCCATAGAATTGTTTCAGCCATTTGTGATTAATCGTTTAATTAGATCGGGAATAGTTAACAAT
ATTAAAGCAGCCAAAAAACTCATTTCTCGTAATGACCCCAGTGTTTGGGACGTTCTGGAAGAAGTCATAGAAGGACACCC
GGTTATGTTAAACCGGGCTCCTACGCTGCATAGGTTGGGAATACAGGCTTTTGAACCCATATTAGTAGAAGGAAGAGCAA
TACAGTTACATCCTCTTGTTTGTCCGGCATTCAATGCTGACTTTGATGGAGATCAGATGGCTGTTCACGTTCCCTTATCG
TTAGAAAGCCAAGCGGAAGCAAGACTGCTAATGTTAGCATCCAATAATATTCTTTCGCCCGCTACGGGTAAACCCATCGT
CACACCCAGTCAAGACATGGTTTTGGGAGCCTATTACCTCACTGCTGAAAACCCCAATGCTAGCAAGGGAGCAGGCAAAT
ACTTTGCCTCCCTAGATGATGTAATTATGGCATATCAACAAGATCAAGTAGAACTACATGCCTATATTTATGTTCGATTC
GATGGCGATGTGCAAACAGGACAACCGGATAACGAACCCTTGGAAGTTACAGAAAACGACGATGGTAGCCGTACTGTCTT
GTATAAATTTCGTCGAGTACGAGAAGATGCTCAAGGGAATTTAATTTCCCAATATATATATACAACTCCTGGCAGAGTGA
TTTATAACAAAGCAATTCAAGATGCTTTAGCCAGCTAA

rbcX

>rbcX TARS_fil_Rr_000000001118:7103-7505
TTATGGAAAATTCCCCTTCTCACCAGCTGGGTTATCTAAACTGTTATCTGGGTAAGGTTCAAATACGCTCAGTTGGGTTA
TCCGCTCAAGATGTTGACGACGCTGCTCCATGTTGGCTTGCTGTATGTTAGTCCGTACCATCTCTGGCAAAAATTCTGTG
ATTTCCGCCGCAATGTACTCCCGCACTGTCATAATCCGCAAGGCTAAATCCGACTTCTCTTGAAACAGCTCTTCGATGTA
TCTCTCTCCATCTTGAACTTTCCCCGCAGAAAAGTTATGTAGCCAAACCGCCAAGGGTGGATTAGTTTCGCTTAACTGCA
CTAACACTGTCCTCAACGCCTGGTATGTTAGGTAGCTCTGGAGAGTTTTGGCAGTCTCTTTGGCAATTTCCCGCAAATTC
AT

nifH

>nifH TARS_fil_Rr_000000003117:340865-341753
CTACTTGCTTGCAGAAGCAGGCTTGCCAATTACTTCAGCGTTTTTGCTGTCATCATCAAGAATACCATACTCAATCAACA
GAGCTTCTAACTCATCCATTTCCATGGGAGTAGGAATAGTTAGCTTGGTGTTATTAATGATCTTTTTACCCAATGCACGG
TATTCATGAGCCTGGTTACTATCGGGTGCATACTCGTTAACTGTCATACGACGCAGTTCTGCGTGTTGAACAATATTGTC
ACGAGGTACGAAGTGAATCATTTGGGTGTTTAAACGTTCAGCTAAGTTTTCAATCAATTCAGCTTCACGGTCAACCTTAC
GGCTATTACAGATTAAACCACCTAAACGCACACCACCGGAGTGAGCATATTTCAAAATACCACGAGCGATGTTGTTTGCA
GCGTACATCGCCATCATTTCACCGGAGGTAACGATGTAGATTTCTTGTGCTTTACCTTCACGAATGGGCATAGCGAAACC
ACCACATACAACGTCACCCAATACGTCGTAGGAAACGAAGTCTAAATCTTGGTAAGCACCATTTTCTTCCAAAAAGTTAA
TGGCAGTGATAATACCACGACCAGCACAACCTACACCGGGTTCTGGACCACCAGATTCCACGCATTTAACACCACGGAAT
CCGGTCAGCATTACTTCTTCCAATTCCAGGTCTTCTACAGCACCTTTCTCAGCAGCCAAGTGCAATACGGTAGTTTGAGC
TTTAGAGTGCAGCATCAAACGGGTAGAGTCAGCTTTGGGGTCGCAACCTACAATTAGAATGCGTTGGCCCATTTCAGCCA
TAGCTGCTAGAGTGTTTTGAGAGGTGGTAGATTTACCAATACCGCCTTTGCCGTAGAATGCTATCTGTCTAATTCTTTCG
TCGCTCAT

cpcA

>cpcA TARS_fil_Rr_000000002618:45755-46244
CTAGCTTAGAGCGTTGATAGCGTAGTCAATGTAGGTATTAGCTTCATTAGCAGCTTGACCGCCCAATCCGTGGTTAGCTT
TGATATATTTTAAAGCTTCCACATACCAACTGGGAGATAGGTTAAAAGCACCGTTAATTTCAGCCAAACCAGCAATCAGG
AATTCATCCAAGGGACCTGTGCCACCAGCAACTAAGCTATAAGTAACAATGCGGAGGTAGTGACCAACGTCACGAGCACA
CTTGGATTTACCACGAGCATCAGCAGCATACTGAGCGCCGGGGGTGGAAGTGGTGTAGGGAAACTTTTGGTATACAGCGT
TAGTAGCACCATCAATTAATTTTTGAGCATTAGCTGTTAAACCACGGGCGGCTTCCATGCTGGCAGCAGCACGTACAAAA
CGACCGTTAACTGCTTGTAATTCAGTATTGCTCAAAAAGCGTCCTTGGGTATCAGCAGCTGCAATAGCTTCGGTAATGGG
GGTTTTCAT

cpcB

>cpcB TARS_fil_Rr_000000002618:46357-46879
CTATGCAACAGCTGCTGCTGCACGATCAAAGTAGCCAGCCAATTCAGAAACTATTTGACTGCAATCGCCCTTGGTAATAC
CGTTGGGATCGTTAACTATCTTGATTGCAGCTTCTTTCATTTTGCCAACACCAACAGCTACGGATGCACCAGGAGTACCC
AAAGCTATATAGGTTTCGCGTAAGCCATTTAAGCAACGGTCATCGAGAACACTAGCGTCACCAGCTAATGCAGCGTAGGT
AACATAGCGTAAGATGATTTCCATATCGCGCAGACAAGCAGCCATGCGACGGTTGGTGTAAGCATTACCACCAGGAGCAA
TTAGTTGGGGCTGTTCTTCAAACAGTGCACGAGCAGCATCGGTAACGATAGCAGAAGCATTGCTTGTAATACGGTTAACA
GTGTCTAGACGTTTGCTGCCGGAAGCTACAACTGCTGTTAAAGCGTCCAACTGTTCAGTGCTCAAAAATTCGCCTCTGGC
GTCAGCTTGGGAAACAACCTTGGAAAATACATCTAATGTCAT

(C)

Lake Trasimeno, unbinned *R. raciborskii* 16S rRNA

```
>16S ribosomal RNA 16S_rRNA::TARS_fil_Rr_000000002915:24-1509(+)
ACGGAGAGTTTGATCCTGGCTCAGGATGAACGCTGGCGGTATGCTTAACACATGCAAGTCGAACGGGATGCTTAGGCATCTAGTGGCGGACGGGTGAGTA
ACGCGTGAGAATCTGGCTCCAGGTCGGGGATAACAGTTGGAAACGACTGCTAATACCGGATGTGCCGAGAGGTGAAAGATTTATCGCCTGGAGATGAGCT
CGCGTCTGATTAGCTAGTTGGCGGTGTAAGGGACCACCAAGGCGTCGATCAGTAGCTGGTCTGAGAGGATGATCAGCCACACTGGGACTGAGACACGGCC
CAGACTCCTACGGGAGGCAGCAGTGGGGAATTTTCCGCAATGGGCGAAAGCCTGACGGAGCAATACCGCGTGAGGGAGGAAGGCTCTTGGGTCGTAAACC
TCTTTTCTCAAGGAAGAAGAAAGTGACGGTACTTGAGGAATAAGCATCGGCTAACTCCGTGCCAGCAGCCGCGGTAATACGGAGGATGCAAGCGTTATCC
GGAATGATTGGGCGTAAAGGGTCTGCAGGTGGAACTGAAAGTCTGCTGTTAAAGAGTTTGGCTTAACCAAATAAAAGCGGTGGAAACTACAGAACTAGAG
TGCGGTAGGGGCAAAAGGAATTCCTGGTGTAGCGGTGAAATGCGTAGATATCAGGAAGAACACCGGTGGCGAAAGCGTTTTGCTAGACCGTAACTGACAC
TGAGGGACGAAAGCTAGGGGAGCGAATGGGATTAGATACCCCAGTAGTCCTAGCCGTAAACGATGGATACTAGGCGTGGCTTGTATCGACCCGAGCCGTG
CCGGAGCTAACGCGTTAAGTATCCCGCCTGGGGAGTACGCACGCAAGTGTGAAACTCAAAGGAATTGACGGGGGCCCGCACAAGCGGTGGAGTATGTGGT
TTAATTCGATGCAACGCGAAGAACCTTACCAAGGCTTGACATCCTGCGAATCCTGGTGAAAGCTGGGAGTGCCTTAGGGAGCGCAGAGACAGGTGGTGCA
TGGCTGTCGTCAGCTCGTGTCGTGAGATGTTGGGTTAAGTCCCGCAACGAGCGCAACCCTCGTTTTTAGTTGCCAGCATTAAGTTGGGCACTCTAGAGAG
ACTGCCGGTGACAAACCGGAGGAAGGTGAGGATGACGTCAAGTCAGCATGCCCCTTACGTCTTGGGCTACACACGTACTACAATGCTACGGACAGAGGGC
AGCGAGCCAGGGATGGCAAGCGAATCCAGAAACCGTAGCTCAGTTCAGATCGAAGGCTGCAACTCGCCTTCGTGAAGGAGGAATCGCTAGTAATTGCAGG
TCAGCATACTGCAGTGAATTCGTTCCCGGGCCTTGTACACACCGCCCGTCACACCATGGAAGTTGGTCACGCCCGAAGTCATTACCCCAACCGAAAGGAG
GGGGATGCCTAAGGTAGGACTGGTGACTGGGGTGAAGTCGTAACAAGGTAGCCGTACCGGAAGGTGTGGCTGGATCACCTCCTTT
```

Table S3

Module pathwise completeness matrix produced by anvio. For every genome analysed in this work, completeness is estimated by computing the fraction of components KOs identified in the genome. In anvio, a threshold of 0.75 is adopted to assess the completeness of a module. A single M number or a combination of M numbers can be used for characterizing phenotypic features encoded in the genome (Kanehisa and Sato 2020). The genomes are reported in the order given in the phylogenomic tree (Fig. 1). Modules not represented in the strains are highlighted.

module_class = Pathway modules

				R2B			R2A								R1B		R1A																							
				1	2	3	4	5	6	7	8	9	10	11	12	13	14	15	16	17	18	19	20	21	22	23	24	25	26	27	28	29	30	31	32	33	34	35	36	37
**module_category**	**module_subcategory**	**module**	**module_name**	**R_brookii_USA**	**Strain_Lake_Trasimeno_ITA**	**C_raciborskii_KL1_USA**	**R_brookii_D9_BRA**	**C_raciborskii_CENA303_BRA**	**C_raciborskii_ITEP_A1_BRA**	**C_raciborskii_PAMP2012_BRA**	**C_raciborskii_PAMP2011_BRA**	**C_raciborskii_CENA302_BRA**	**C_raciborskii_MVCC19_URY**	**C_raciborskii_MVCC14_URY**	**R_curvata_NIES_932_JPN**	**C_curvispora_GIHE_G1_KOR**	**C_raciborskii_CHAB3438_CHN**	**C_raciborskii_1523720_IND**	**C_raciborskii_DSH_CHN**	**Cylindrospermopsis_sp_CR12_SGP**	**C_raciborskii_N8_CHN**	**Cyanobacteria_bacterium_REEB494_CHN**	**C_raciborskii_GIHE_2018_KOR**	**C_raciborskii_KLL07_ISR**	**C_raciborskii_UAM_DH_KmRr_POL**	**C_raciborskii_Cr2010_NLD**	**C_raciborskii_UAM_DH_BiRr_POL**	**C_raciborskii_UAM_DH_ZRr_POL**	**C_raciborskii_UAM_DH_MRr_POL**	**C_raciborskii_CS_508_AUS**	**C_raciborskii_CS_505_AUS**	**C_raciborskii_C07_AUS**	**C_raciborskii_C04_AUS**	**C_raciborskii_C03_AUS**	**C_raciborskii_S01_AUS**	**C_raciborskii_S14_AUS**	**C_raciborskii_S05_AUS**	**C_raciborskii_S10_AUS**	**C_raciborskii_S07_AUS**	**C_raciborskii_S06_AUS**
Carbohydrate metabolism	Central carbohydrate metabolism	M00001	Glycolysis (Embden-Meyerhof pathway), glucose => pyruvate	1	1	1	1	1	1	1	1	1	1	1	1	1	1	1	1	1	1	1	0.9	1	1	1	1	1	1	1	1	1	1	1	1	1	1	1	1	1
		M00002	Glycolysis, core module involving three-carbon compounds	1	1	1	1	1	1	1	1	1	1	1	1	1	1	1	1	1	1	1	1	1	1	1	1	1	1	1	1	1	1	1	1	1	1	1	1	1
		M00003	Gluconeogenesis, oxaloacetate => fructose-6P	0.875	0.875	0.875	0.875	0.875	0.875	0.875	0.875	0.875	0.875	0.875	0.875	0.875	0.875	0.875	0.875	0.875	0.875	0.875	0.875	0.875	0.875	0.875	0.875	0.875	0.875	0.875	0.875	0.875	0.875	0.875	0.875	0.875	0.875	0.875	0.875	0.875
		M00004	Pentose phosphate pathway (Pentose phosphate cycle)	1	1	1	1	1	1	1	1	1	1	1	1	1	1	1	1	1	1	1	1	1	1	1	1	1	1	1	1	1	1	1	1	1	1	1	1	1
		M00005	PRPP biosynthesis, ribose 5P => PRPP	1	1	1	1	1	1	1	1	1	1	1	1	1	1	1	1	1	1	1	1	1	1	1	1	1	1	1	1	1	1	1	1	1	1	1	1	1
		M00006	Pentose phosphate pathway, oxidative phase, glucose 6P => ribulose 5P	1	1	1	1	1	1	1	1	1	1	1	1	1	1	1	1	1	1	1	1	1	1	1	1	1	1	1	1	1	1	1	1	1	1	1	1	1
		M00007	Pentose phosphate pathway, non-oxidative phase, fructose 6P => ribose 5P	1	1	1	1	1	1	1	1	1	1	1	1	1	1	1	1	1	1	1	1	1	1	1	1	1	1	1	1	1	1	1	1	1	1	1	1	1
		M00008	Entner-Doudoroff pathway, glucose-6P => glyceraldehyde-3P + pyruvate	0.75	0.75	0.75	0.75	0.75	0.75	0.75	0.75	0.75	0.75	0.75	0.75	0.75	0.75	0.75	0.75	0.75	0.75	0.75	0.75	0.75	0.75	0.75	0.75	0.75	0.75	0.75	0.75	0.75	0.75	0.75	0.75	0.75	0.75	0.75	0.75	0.75
		M00009	Citrate cycle (TCA cycle, Krebs cycle)	0.583333	0.583333	0.583333	0.583333	0.583333	0.583333	0.583333	0.583333	0.583333	0.583333	0.583333	0.583333	0.583333	0.583333	0.583333	0.583333	0.583333	0.583333	0.583333	0.583333	0.583333	0.583333	0.583333	0.583333	0.583333	0.583333	0.583333	0.583333	0.583333	0.583333	0.583333	0.583333	0.583333	0.583333	0.583333	0.583333	0.583333
		M00010	Citrate cycle, first carbon oxidation, oxaloacetate => 2-oxoglutarate	1	1	1	1	1	1	1	1	1	1	1	1	1	1	1	1	1	1	1	1	1	1	1	1	1	1	1	1	1	1	1	1	1	1	1	1	1
		M00011	Citrate cycle, second carbon oxidation, 2-oxoglutarate => oxaloacetate	0.333333	0.333333	0.333333	0.333333	0.333333	0.333333	0.333333	0.333333	0.333333	0.333333	0.333333	0.333333	0.333333	0.333333	0.333333	0.333333	0.333333	0.333333	0.333333	0.333333	0.333333	0.333333	0.333333	0.333333	0.333333	0.333333	0.333333	0.333333	0.333333	0.333333	0.333333	0.333333	0.333333	0.333333	0.333333	0.333333	0.333333
		M00307	Pyruvate oxidation, pyruvate => acetyl-CoA	1	1	1	1	1	1	1	1	1	1	1	1	1	1	1	1	1	1	1	1	1	1	1	1	1	1	1	1	1	1	1	1	1	1	1	1	1
		M00308	Semi-phosphorylative Entner-Doudoroff pathway, gluconate => glycerate-3P	0.6	0.6	0.6	0.6	0.6	0.6	0.6	0.6	0.6	0.6	0.6	0.6	0.6	0.6	0.6	0.6	0.6	0.6	0.6	0.6	0.6	0.6	0.6	0.6	0.6	0.6	0.6	0.6	0.6	0.6	0.6	0.6	0.6	0.6	0.6	0.6	0.6
		M00580	Pentose phosphate pathway, archaea, fructose 6P => ribose 5P	0.5	0.5	0.5	0.5	0.5	0.5	0.5	0.5	0.5	0.5	0.5	0.5	0.5	0.5	0.5	0.5	0.5	0.5	0.5	0.5	0.5	0.5	0.5	0.5	0.5	0.5	0.5	0.5	0.5	0.5	0.5	0.5	0.5	0.5	0.5	0.5	0.5
	Other carbohydrate metabolism	M00012	Glyoxylate cycle	0.4	0.4	0.4	0.4	0.4	0.4	0.4	0.4	0.4	0.4	0.4	0.4	0.4	0.4	0.4	0.4	0.4	0.4	0.4	0.4	0.4	0.4	0.4	0.4	0.4	0.4	0.4	0.4	0.4	0.4	0.4	0.4	0.4	0.4	0.4	0.4	0.4
		M00014	Glucuronate pathway (uronate pathway)	0.111111	0.111111	0.111111	0.111111	0.111111	0.111111	0.111111	0.111111	0.111111	0.111111	0.111111	0.111111	0.111111	0.111111	0.111111	0.111111	0.111111	0.111111	0.111111	0.111111	0.111111	0.111111	0.111111	0.111111	0.111111	0.111111	0.111111	0.111111	0.111111	0.111111	0.111111	0.111111	0.111111	0.111111	0.111111	0.111111	0.111111
		M00061	D-Glucuronate degradation, D-glucuronate => pyruvate + D-glyceraldehyde 3P	0.2	0.2	0.2	0.2	0.2	0.2	0.2	0.2	0.2	0.2	0.2	0.2	0.2	0.2	0.2	0.2	0.2	0.2	0.2	0.2	0.2	0.2	0.2	0.2	0.2	0.2	0.2	0.2	0.2	0.2	0.2	0.2	0.2	0.2	0.2	0.2	0.2
		M00129	Ascorbate biosynthesis, animals, glucose-1P => ascorbate	0.142857	0.142857	0.142857	0.142857	0.285714	0.142857	0.142857	0.142857	0.142857	0.142857	0.142857	0.142857	0.142857	0.142857	0.142857	0.142857	0.142857	0.142857	0.142857	0.142857	0.142857	0.142857	0.142857	0.142857	0.142857	0.142857	0.142857	0.142857	0.142857	0.142857	0.142857	0.142857	0.142857	0.142857	0.142857	0.142857	0.142857
		M00131	Inositol phosphate metabolism, Ins(1,3,4,5)P4 => Ins(1,3,4)P3 => myo-inositol	0.25	0.25	0.25	0.25	0.25	0.25	0.25	0.25	0.25	0.25	0.25	0.25	0.25	0.25	0.25	0.25	0.25	0.25	0.25	0.25	0.25	0.25	0.25	0.25	0.25	0.25	0.25	0.25	0.25	0.25	0.25	0.25	0.25	0.25	0.25	0.25	0.25
		M00532	Photorespiration	0.4	0.4	0.4	0.4	0.4	0.4	0.4	0.4	0.4	0.4	0.4	0.4	0.4	0.4	0.4	0.4	0.4	0.4	0.4	0.4	0.4	0.4	0.4	0.4	0.4	0.4	0.4	0.4	0.4	0.4	0.4	0.4	0.4	0.4	0.4	0.4	0.4
		M00549	Nucleotide sugar biosynthesis, glucose => UDP-glucose	0.333333	0.333333	0.333333	0.333333	0.333333	0.333333	0.333333	0.333333	0.333333	0.333333	0.333333	0.333333	0.333333	0.333333	0.333333	0.333333	0.333333	0.333333	0.333333	0.333333	0.333333	0.333333	0.333333	0.333333	0.333333	0.333333	0.333333	0.333333	0.333333	0.333333	0.333333	0.333333	0.333333	0.333333	0.333333	0.333333	0.333333
		M00552	D-galactonate degradation, De Ley-Doudoroff pathway, D-galactonate => glycerate-3P	0.4	0.4	0.4	0.4	0.4	0.4	0.4	0.4	0.4	0.4	0.4	0.4	0.4	0.4	0.4	0.4	0.4	0.4	0.4	0.4	0.4	0.4	0.4	0.4	0.4	0.4	0.4	0.4	0.4	0.4	0.4	0.4	0.4	0.4	0.4	0.4	0.4
		M00565	Trehalose biosynthesis, D-glucose 1P => trehalose	0.5	0.5	0.5	0.5	0.666667	0.666667	0.5	0.666667	0.5	0.5	0.5	0.666667	0.5	0.666667	0.5	0.666667	0.666667	0.666667	0.666667	0.666667	0.5	0.666667	0.5	0.5	0.5	0.5	0.5	0.666667	0.5	0.5	0.5	0.666667	0.666667	0.666667	0.666667	0.666667	0.666667
		M00631	D-Galacturonate degradation (bacteria), D-galacturonate => pyruvate + D-glyceraldehyde 3P	0.2	0.2	0.2	0.2	0.2	0.2	0.2	0.2	0.2	0.2	0.2	0.2	0.2	0.2	0.2	0.2	0.2	0.2	0.2	0.2	0.2	0.2	0.2	0.2	0.2	0.2	0.2	0.2	0.2	0.2	0.2	0.2	0.2	0.2	0.2	0.2	0.2
		M00632	Galactose degradation, Leloir pathway, galactose => alpha-D-glucose-1P	0.5	0.5	0.5	0.5	0.5	0.5	0.5	0.5	0.5	0.5	0.5	0.5	0.5	0.5	0.5	0.5	0.5	0.5	0.5	0.5	0.5	0.5	0.5	0.5	0.5	0.5	0.5	0.5	0.5	0.5	0.5	0.5	0.5	0.5	0.5	0.5	0.5
		M00740	Methylaspartate cycle	0.181818	0.181818	0.181818	0.181818	0.181818	0.181818	0.181818	0.181818	0.181818	0.181818	0.181818	0.272727	0.181818	0.181818	0.181818	0.181818	0.181818	0.181818	0.181818	0.181818	0.181818	0.181818	0.181818	0.181818	0.181818	0.181818	0.181818	0.181818	0.181818	0.181818	0.181818	0.181818	0.181818	0.181818	0.181818	0.181818	0.181818
		M00854	Glycogen biosynthesis, glucose-1P => glycogen/starch	1	1	1	1	1	1	1	1	1	1	1	1	1	1	1	1	1	1	1	1	1	1	1	1	1	1	1	1	1	1	1	1	1	1	1	1	1
		M00855	Glycogen degradation, glycogen => glucose-6P	0.5	0.5	0.5	0.5	0.5	0.5	0.333333	0.5	0.5	0.5	0.5	0.5	0.5	0.5	0.5	0.5	0.5	0.5	0.5	0.5	0.5	0.5	0.5	0.5	0.5	0.5	0.333333	0.5	0.5	0.5	0.5	0.5	0.5	0.5	0.5	0.5	0.5
		M00892	UDP-N-acetyl-D-glucosamine biosynthesis, eukaryotes, glucose => UDP-GlcNAc	0.5	0.5	0.5	0.5	0.5	0.5	0.5	0.5	0.5	0.5	0.5	0.5	0.5	0.5	0.5	0.5	0.5	0.5	0.5	0.5	0.5	0.5	0.5	0.5	0.5	0.5	0.5	0.5	0.5	0.5	0.5	0.5	0.5	0.5	0.5	0.5	0.5
		M00909	UDP-N-acetyl-D-glucosamine biosynthesis, prokaryotes, glucose => UDP-GlcNAc	1	1	1	1	1	1	1	1	1	1	1	1	1	1	1	1	1	1	1	1	1	1	1	1	1	1	1	1	1	1	1	1	1	1	1	1	1
		M00968	Pentose bisphosphate pathway (nucleoside degradation), archaea, nucleoside/NMP => 3-PGA/glycerone phosphate	0.333333	0.333333	0.333333	0.333333	0.333333	0.333333	0.333333	0.333333	0.333333	0.333333	0.333333	0.333333	0.333333	0.333333	0.333333	0.333333	0.333333	0.333333	0.333333	0.333333	0.333333	0.333333	0.333333	0.333333	0.333333	0.333333	0.333333	0.333333	0.333333	0.333333	0.333333	0.333333	0.333333	0.333333	0.333333	0.333333	0.333333
Energy metabolism	Carbon fixation	M00165	Reductive pentose phosphate cycle (Calvin cycle)	0.909091	0.909091	0.909091	0.909091	0.909091	0.909091	0.909091	0.909091	0.909091	0.909091	0.909091	1	0.909091	0.909091	0.909091	0.909091	0.909091	0.909091	0.909091	0.909091	0.909091	0.909091	0.909091	0.909091	0.909091	0.909091	0.909091	0.909091	0.909091	0.909091	0.909091	0.909091	0.909091	0.909091	0.909091	0.909091	0.909091
		M00166	Reductive pentose phosphate cycle, ribulose-5P => glyceraldehyde-3P	1	1	1	1	1	1	1	1	1	1	1	1	1	1	1	1	1	1	1	1	1	1	1	1	1	1	1	1	1	1	1	1	1	1	1	1	1
		M00167	Reductive pentose phosphate cycle, glyceraldehyde-3P => ribulose-5P	0.857143	0.857143	0.857143	0.857143	0.857143	0.857143	0.857143	0.857143	0.857143	0.857143	0.857143	1	0.857143	0.857143	0.857143	0.857143	0.857143	0.857143	0.857143	0.857143	0.857143	0.857143	0.857143	0.857143	0.857143	0.857143	0.857143	0.857143	0.857143	0.857143	0.857143	0.857143	0.857143	0.857143	0.857143	0.857143	0.857143
		M00168	CAM (Crassulacean acid metabolism), dark	0.5	0.5	0.5	0.5	0.5	0.5	0.5	0.5	0.5	0.5	0.5	0.5	0.5	0.5	0.5	0.5	0.5	0.5	0.5	0.5	0.5	0.5	0.5	0.5	0.5	0.5	0.5	0.5	0.5	0.5	0.5	0.5	0.5	0.5	0.5	0.5	0.5
		M00170	C4-dicarboxylic acid cycle, phosphoenolpyruvate carboxykinase type	0.25	0.25	0.25	0.25	0.25	0.25	0.25	0.25	0.25	0.25	0.25	0.25	0.25	0.25	0.25	0.25	0.25	0.25	0.25	0.25	0.25	0.25	0.25	0.25	0.25	0.25	0.25	0.25	0.25	0.25	0.25	0.25	0.25	0.25	0.25	0.25	0.25
		M00171	C4-dicarboxylic acid cycle, NAD - malic enzyme type	0.142857	0.142857	0.142857	0.142857	0.142857	0.142857	0.142857	0.142857	0.142857	0.142857	0.142857	0.142857	0.142857	0.142857	0.142857	0.142857	0.142857	0.142857	0.142857	0.142857	0.142857	0.142857	0.142857	0.142857	0.142857	0.142857	0.142857	0.142857	0.142857	0.142857	0.142857	0.142857	0.142857	0.142857	0.142857	0.142857	0.142857
		M00172	C4-dicarboxylic acid cycle, NADP - malic enzyme type	0.25	0.25	0.25	0.25	0.25	0.25	0.25	0.25	0.25	0.25	0.25	0.25	0.25	0.25	0.25	0.25	0.25	0.25	0.25	0.25	0.25	0.25	0.25	0.25	0.25	0.25	0.25	0.25	0.25	0.25	0.25	0.25	0.25	0.25	0.25	0.25	0.25
		M00173	Reductive citrate cycle (Arnon-Buchanan cycle)	0.590909	0.590909	0.590909	0.590909	0.590909	0.590909	0.590909	0.590909	0.590909	0.590909	0.590909	0.590909	0.590909	0.590909	0.590909	0.590909	0.590909	0.590909	0.590909	0.590909	0.590909	0.590909	0.590909	0.590909	0.590909	0.590909	0.590909	0.590909	0.590909	0.590909	0.590909	0.590909	0.590909	0.590909	0.590909	0.590909	0.590909
		M00374	Dicarboxylate-hydroxybutyrate cycle	0.269231	0.269231	0.269231	0.269231	0.269231	0.269231	0.269231	0.269231	0.269231	0.269231	0.269231	0.269231	0.269231	0.269231	0.269231	0.269231	0.269231	0.269231	0.269231	0.269231	0.269231	0.269231	0.269231	0.269231	0.269231	0.269231	0.269231	0.269231	0.269231	0.269231	0.269231	0.269231	0.269231	0.269231	0.269231	0.269231	0.269231
		M00376	3-Hydroxypropionate bi-cycle	0.128205	0.128205	0.128205	0.108974	0.128205	0.128205	0.128205	0.128205	0.128205	0.128205	0.128205	0.128205	0.128205	0.128205	0.128205	0.128205	0.128205	0.128205	0.128205	0.128205	0.128205	0.128205	0.128205	0.128205	0.128205	0.128205	0.128205	0.128205	0.128205	0.128205	0.128205	0.128205	0.128205	0.128205	0.128205	0.128205	0.128205
		M00377	Reductive acetyl-CoA pathway (Wood-Ljungdahl pathway)	0.285714	0.285714	0.285714	0.285714	0.285714	0.285714	0.285714	0.285714	0.285714	0.285714	0.285714	0.285714	0.285714	0.285714	0.285714	0.285714	0.285714	0.285714	0.285714	0.285714	0.285714	0.285714	0.285714	0.285714	0.285714	0.285714	0.285714	0.285714	0.285714	0.285714	0.285714	0.285714	0.285714	0.285714	0.285714	0.285714	0.285714
		M00579	Phosphate acetyltransferase-acetate kinase pathway, acetyl-CoA => acetate	0.5	0.5	0.5	0	0.5	0.5	0.5	0.5	0	0	0	0	0	0	0	0	0	0	0	0	0	0	0	0	0	0	0	0	0	0	0	0	0	0	0	0	0
		M00620	Incomplete reductive citrate cycle, acetyl-CoA => oxoglutarate	0.071429	0.071429	0.071429	0.071429	0.071429	0.071429	0.071429	0.071429	0.071429	0.071429	0.071429	0.071429	0.071429	0.071429	0.071429	0.071429	0.071429	0.071429	0.071429	0.071429	0.071429	0.071429	0.071429	0.071429	0.071429	0.071429	0.071429	0.071429	0.071429	0.071429	0.071429	0.071429	0.071429	0.071429	0.071429	0.071429	0.071429
	Methane metabolism	M00344	Formaldehyde assimilation, xylulose monophosphate pathway	0.5	0.5	0.5	0.5	0.5	0.5	0.5	0.5	0.5	0.5	0.5	0.5	0.5	0.5	0.5	0.5	0.5	0.5	0.5	0.5	0.5	0.5	0.5	0.5	0.5	0.5	0.5	0.5	0.5	0.5	0.5	0.5	0.5	0.5	0.5	0.5	0.5
		M00345	Formaldehyde assimilation, ribulose monophosphate pathway	0.666667	0.666667	0.666667	0.666667	0.666667	0.666667	0.666667	0.666667	0.666667	0.666667	0.666667	0.666667	0.666667	0.666667	0.666667	0.666667	0.666667	0.666667	0.666667	0.333333	0.666667	0.666667	0.666667	0.666667	0.666667	0.666667	0.666667	0.666667	0.666667	0.666667	0.666667	0.666667	0.666667	0.666667	0.666667	0.666667	0.666667
		M00346	Formaldehyde assimilation, serine pathway	0.333333	0.333333	0.333333	0.333333	0.333333	0.333333	0.333333	0.333333	0.333333	0.333333	0.333333	0.333333	0.333333	0.333333	0.333333	0.333333	0.333333	0.333333	0.333333	0.333333	0.333333	0.333333	0.333333	0.333333	0.333333	0.333333	0.333333	0.333333	0.333333	0.333333	0.333333	0.333333	0.333333	0.333333	0.333333	0.333333	0.333333
		M00357	Methanogenesis, acetate => methane	0.166667	0.2	0.2	0.2	0.2	0.2	0.2	0.2	0.2	0.2	0.2	0.2	0.2	0.2	0.2	0.2	0.2	0.2	0.2	0.2	0.2	0.2	0.2	0.2	0.2	0.2	0.2	0.2	0.2	0.2	0.2	0.2	0.2	0.2	0.2	0.2	0.2
		M00358	Coenzyme M biosynthesis	0.25	0.25	0.25	0.25	0.25	0.25	0.25	0.25	0.25	0.25	0.25	0.25	0.25	0.25	0.25	0.25	0.25	0.25	0.25	0.25	0.25	0.25	0.25	0.25	0.25	0.25	0.25	0.25	0.25	0.25	0.25	0.25	0.25	0.25	0.25	0.25	0.25
		M00378	F420 biosynthesis, archaea	0.25	0.25	0.25	0.25	0.25	0.25	0.25	0.25	0.25	0.25	0.25	0.25	0.25	0.25	0.25	0.25	0.25	0.25	0.25	0.25	0.25	0.25	0.25	0.25	0.25	0.25	0.25	0.25	0.25	0.25	0.25	0.25	0.25	0.25	0.25	0.25	0.25
	Nitrogen metabolism	M00175	Nitrogen fixation, nitrogen => ammonia	1	1	1	0	0	1	1	1	1	1	1	0	1	1	1	1	1	1	1	1	1	1	1	1	1	1	1	1	1	1	1	1	1	1	1	1	1
		M00531	Assimilatory nitrate reduction, nitrate => ammonia	1	1	1	0.5	1	1	1	1	1	1	1	1	1	1	1	1	1	1	1	1	1	1	1	1	1	1	1	1	1	1	1	1	1	1	1	1	1
	Sulfur metabolism	M00176	Assimilatory sulfate reduction, sulfate => H2S	0.833333	0.833333	0.833333	0.833333	0.833333	0.833333	0.833333	0.833333	0.833333	0.833333	0.833333	0.833333	0.833333	0.833333	0.666667	0.833333	0.833333	0.833333	0.833333	0.833333	0.833333	0.833333	0.833333	0.833333	0.833333	0.833333	0.833333	0.833333	0.833333	0.833333	0.833333	0.833333	0.5	0.833333	0.833333	0.833333	0.833333
		M00596	Dissimilatory sulfate reduction, sulfate => H2S	0.333333	0.333333	0.333333	0.5	0.5	0.5	0.333333	0.333333	0.5	0.333333	0.333333	0.333333	0.333333	0.333333	0	0.333333	0.333333	0.333333	0.333333	0.333333	0.333333	0.333333	0.333333	0.333333	0.333333	0.333333	0.333333	0.333333	0.333333	0.333333	0.333333	0.333333	0.333333	0.333333	0.333333	0.333333	0.333333
	Photosynthesis	M00161	Photosystem II	1	0.833333	1	1	0.833333	1	1	1	1	1	0.833333	1	1	1	1	1	1	1	1	1	1	1	1	1	1	1	0.833333	1	1	1	1	1	1	1	1	1	1
		M00163	Photosystem I	0.833333	1	1	1	1	1	1	1	1	1	1	1	1	1	1	1	1	1	1	1	1	1	1	1	1	1	0.833333	1	1	1	1	1	1	1	1	1	1
	ATP synthesis	M00144	NADH:quinone oxidoreductase, prokaryotes	0.75	0.75	0.75	0.75	0.75	0.75	0.75	0.75	0.75	0.75	0.75	0.75	0.75	0.75	0.75	0.75	0.75	0.75	0.75	0.75	0.75	0.75	0.75	0.75	0.75	0.75	0.75	0.75	0.75	0.75	0.75	0.75	0.75	0.75	0.75	0.75	0.75
		M00145	NAD(P)H:quinone oxidoreductase, chloroplasts and cyanobacteria	0.909091	1	1	1	1	1	1	1	1	1	1	1	1	1	1	1	1	1	1	1	1	1	1	1	1	1	1	1	1	1	1	1	1	1	1	1	1
		M00149	Succinate dehydrogenase, prokaryotes	0.5	0.5	0.5	0.5	0.5	0.5	0.5	0.5	0.5	0.5	0.5	0.5	0.5	0.5	0.5	0.5	0.5	0.5	0.5	0.5	0.5	0.5	0.5	0.5	0.5	0.5	0.5	0.5	0.5	0.5	0.5	0.5	0.5	0.5	0.5	0.5	0.5
		M00153	Cytochrome bd ubiquinol oxidase	0.666667	0.666667	0.666667	0.666667	0.666667	0.666667	0.666667	0.666667	0.666667	0	0	0.666667	0	0.666667	0.666667	0.666667	0.666667	0.666667	0.666667	0.666667	0.666667	0.666667	0.666667	0.666667	0.666667	0.666667	0.666667	0.666667	0.666667	0.666667	0.666667	0.666667	0.666667	0.666667	0.666667	0.666667	0.666667
		M00154	Cytochrome c oxidase	0.133333	0.133333	0.133333	0.133333	0.133333	0.133333	0.133333	0.133333	0.133333	0.133333	0.133333	0.133333	0.133333	0.133333	0.133333	0.133333	0.133333	0.133333	0.133333	0.133333	0.133333	0.133333	0.133333	0.133333	0.133333	0.133333	0.133333	0.133333	0.133333	0.133333	0.133333	0.133333	0.133333	0.133333	0.133333	0.133333	0.133333
		M00155	Cytochrome c oxidase, prokaryotes	1	1	1	1	1	1	1	1	1	1	1	1	1	1	1	1	1	1	1	1	1	1	1	1	1	1	1	1	1	1	1	1	1	1	1	1	1
		M00157	F-type ATPase, prokaryotes and chloroplasts	0.25	1	1	1	1	1	1	1	1	1	1	1	1	1	1	1	1	1	1	1	1	1	1	1	1	1	1	1	1	1	1	1	1	1	1	1	1
		M00162	Cytochrome b6f complex	0.75	0.75	0.75	0.75	0.75	0.75	0.75	0.75	0.75	0.75	0.75	0.75	0.75	0.75	0.75	0.75	0.75	0.75	0.75	0.75	0.75	0.75	0.75	0.75	0.75	0.75	0.75	0.75	0.75	0.75	0.75	0.75	0.75	0.75	0.75	0.75	0.75
Lipid metabolism	Fatty acid metabolism	M00082	Fatty acid biosynthesis, initiation	1	1	1	0.916667	1	1	1	1	1	1	1	1	1	1	1	1	1	1	1	1	1	1	1	1	1	1	1	1	1	1	1	1	1	1	1	1	1
		M00083	Fatty acid biosynthesis, elongation	1	1	1	1	1	1	1	1	1	1	1	1	1	1	1	1	1	1	1	1	1	1	1	1	1	1	1	1	1	1	1	1	1	1	1	1	1
		M00086	beta-Oxidation, acyl-CoA synthesis	1	1	1	0	1	1	1	1	1	1	1	1	1	1	1	1	1	1	1	1	1	1	1	1	1	1	1	1	1	1	1	1	1	1	1	1	1
		M00873	Fatty acid biosynthesis in mitochondria, animals	0.25	0.25	0.25	0.25	0.25	0.25	0.25	0.25	0.25	0.25	0.25	0.25	0.25	0.25	0.25	0.25	0.25	0.25	0.25	0.25	0.25	0.25	0.25	0.25	0.25	0.25	0.25	0.25	0.25	0.25	0.25	0.25	0.25	0.25	0.25	0.25	0.25
		M00874	Fatty acid biosynthesis in mitochondria, fungi	0.416667	0.416667	0.416667	0.416667	0.416667	0.416667	0.416667	0.416667	0.416667	0.416667	0.416667	0.416667	0.416667	0.416667	0.416667	0.416667	0.416667	0.416667	0.416667	0.416667	0.416667	0.416667	0.416667	0.416667	0.416667	0.416667	0.416667	0.416667	0.416667	0.416667	0.416667	0.416667	0.416667	0.416667	0.416667	0.416667	0.416667
	Sterol biosynthesis	M00101	Cholesterol biosynthesis, squalene 2,3-epoxide => cholesterol	0.1	0.1	0.1	0.1	0.1	0.1	0.1	0.1	0.1	0.1	0.1	0.1	0.1	0.1	0.1	0.1	0.1	0.1	0.1	0.1	0.1	0.1	0.1	0.1	0.1	0.1	0.1	0.1	0.1	0.1	0.1	0.1	0.1	0.1	0.1	0.1	0.1
		M00102	Ergocalciferol biosynthesis, squalene 2,3-epoxide => ergosterol/ergocalciferol	0.090909	0.090909	0.090909	0.090909	0.090909	0.090909	0.090909	0.090909	0.090909	0.090909	0.090909	0.090909	0.090909	0.090909	0.090909	0.090909	0.090909	0.090909	0.090909	0.090909	0.090909	0.090909	0.090909	0.090909	0.090909	0.090909	0.090909	0.090909	0.090909	0.090909	0.090909	0.090909	0.090909	0.090909	0.090909	0.090909	0.090909
		M00917	Phytosterol biosynthesis, squalene 2,3-epoxide => campesterol/sitosterol	0.071429	0.071429	0.071429	0.071429	0.071429	0.071429	0.071429	0.071429	0.071429	0.071429	0.071429	0.071429	0.071429	0.071429	0.071429	0.071429	0.071429	0.071429	0.071429	0.071429	0.071429	0.071429	0.071429	0.071429	0.071429	0.071429	0.071429	0.071429	0.071429	0.071429	0.071429	0.071429	0.071429	0.071429	0.071429	0.071429	0.071429
	Lipid metabolism	M00066	Lactosylceramide biosynthesis	0.5	0.5	0.5	0	0	0.5	0.5	0.5	0.5	0.5	0.5	0	0.5	0.5	0.5	0.5	0.5	0.5	0.5	0.5	0.5	0.5	0.5	0.5	0.5	0.5	0.5	0.5	0.5	0.5	0.5	0.5	0	0.5	0.5	0.5	0.5
		M00089	Triacylglycerol biosynthesis	0.25	0.25	0.25	0.25	0.25	0.25	0.25	0.25	0.25	0.25	0.25	0.25	0.25	0.25	0.25	0.25	0.25	0.25	0.25	0.25	0.25	0.25	0.25	0.25	0.25	0.25	0.25	0.25	0.25	0.25	0.25	0.25	0.25	0.25	0.25	0.25	0.25
		M00093	Phosphatidylethanolamine (PE) biosynthesis, PA => PS => PE	0.333333	0.333333	0.333333	0.333333	0.333333	0.333333	0.333333	0.333333	0.333333	0.333333	0.333333	0.333333	0.333333	0.333333	0.333333	0.333333	0.333333	0.333333	0.333333	0.333333	0.333333	0.333333	0.333333	0.333333	0.333333	0.333333	0	0.333333	0.333333	0.333333	0.333333	0.333333	0.333333	0.333333	0.333333	0.333333	0.333333
Nucleotide metabolism	Purine metabolism	M00048	De novo purine biosynthesis, PRPP + glutamine => IMP	0.875	1	1	1	0.958333	1	1	1	1	1	0.916667	1	1	1	0.875	1	1	1	1	1	1	1	0.916667	1	1	1	1	1	1	1	1	1	1	1	1	1	1
		M00049	Adenine ribonucleotide biosynthesis, IMP => ADP,ATP	0.75	1	1	1	1	1	1	1	1	1	1	1	1	1	0.75	1	1	1	1	1	1	1	1	1	1	1	1	1	1	1	1	1	1	1	1	1	1
		M00050	Guanine ribonucleotide biosynthesis, IMP => GDP,GTP	0.75	0.75	0.75	0.75	0.75	0.75	0.25	0.75	0.75	0.75	0.75	0.75	0.75	0.75	0.75	0.75	0.75	0.75	0.75	0.75	0.75	0.75	0.75	0.75	0.75	0.75	0.75	0.75	0.75	0.75	0.75	0.75	0.75	0.75	0.75	0.75	0.75
		M00053	Deoxyribonucleotide biosynthesis, ADP/GDP/CDP/UDP => dATP/dGTP/dCTP/dUTP	0.5	0.5	0.5	0.5	0.5	0.5	0.5	0.5	0.5	0.5	0.5	0.5	0.5	0.5	0.5	0.5	0.5	0.5	0.5	0.5	0.5	0.5	0.5	0.5	0.5	0.5	0.5	0.5	0.5	0.5	0.5	0.5	0.5	0.5	0.5	0.5	0.5
		M00958	Adenine ribonucleotide degradation, AMP => Urate	0.75	0.75	0.75	0.75	0.75	0.75	0.75	0.75	0.75	0.75	0.75	0.75	0.75	0.75	0.75	0.75	0.75	0.75	0.75	0.75	0.75	0.75	0.75	0.75	0.75	0.75	0.75	0.75	0.75	0.75	0.75	0.75	0.75	0.75	0.75	0.75	0.75
		M00959	Guanine ribonucleotide degradation, GMP => Urate	0.5	0.5	0.5	0.5	0.5	0.5	0.5	0.5	0.5	0.5	0.5	0.5	0.5	0.5	0.5	0.5	0.5	0.5	0.5	0.5	0.5	0.5	0.5	0.5	0.5	0.5	0.5	0.5	0.5	0.5	0.5	0.5	0.5	0.5	0.5	0.5	0.5
	Pyrimidine metabolism	M00051	De novo pyrimidine biosynthesis, glutamine (+ PRPP) => UMP	0.916667	0.916667	0.916667	0.916667	0.916667	0.916667	0.916667	0.916667	0.916667	0.916667	0.916667	0.916667	0.916667	0.916667	0.833333	0.916667	0.916667	0.916667	0.916667	0.916667	0.916667	0.916667	0.916667	0.916667	0.916667	0.916667	0.916667	0.916667	0.916667	0.916667	0.916667	0.916667	0.916667	0.916667	0.916667	0.916667	0.916667
		M00052	Pyrimidine ribonucleotide biosynthesis, UMP => UDP/UTP,CDP/CTP	1	1	1	1	1	1	1	1	1	1	1	1	1	1	1	1	1	1	1	1	1	1	1	1	1	1	1	1	1	1	1	1	1	1	1	1	1
		M00938	Pyrimidine deoxyribonucleotide biosynthesis, UDP => dTTP	0.6	0.6	0.6	0.6	0.6	0.6	0.6	0.8	0.6	0.6	0.6	0.6	0.6	0.6	0.6	0.6	0.6	0.6	0.6	0.6	0.6	0.6	0.6	0.6	0.6	0.6	0.6	0.6	0.6	0.6	0.6	0.6	0.6	0.6	0.6	0.6	0.6
		M00939	Pyrimidine degradation, uracil => 3-hydroxypropanoate	0.2	0.2	0.2	0.2	0.2	0.2	0.2	0.2	0.2	0.2	0.2	0.2	0.2	0.2	0.2	0.2	0.2	0.2	0.2	0	0.2	0.2	0.2	0.2	0.2	0.2	0	0	0	0	0	0	0	0	0	0	0
Amino acid metabolism	Serine and threonine metabolism	M00018	Threonine biosynthesis, aspartate => homoserine => threonine	1	1	1	1	1	1	1	1	1	1	1	1	1	1	1	1	1	1	1	1	1	1	1	1	1	1	1	1	1	1	1	1	1	1	1	1	1
		M00020	Serine biosynthesis, glycerate-3P => serine	0.666667	0.666667	0.666667	0.666667	0.666667	0.666667	0.666667	0.666667	0.666667	0.666667	0.666667	0.666667	0.666667	0.666667	0.666667	0.666667	0.666667	0.666667	0.666667	0.666667	0.666667	0.666667	0.666667	0.666667	0.666667	0.666667	0.666667	0.666667	0.666667	0.666667	0.666667	0.666667	0.666667	0.666667	0.666667	0.666667	0.666667
		M00033	Ectoine biosynthesis, aspartate => ectoine	0.4	0.4	0.4	0.4	0.4	0.4	0.4	0.4	0.4	0.4	0.4	0.4	0.4	0.4	0.4	0.4	0.4	0.4	0.4	0.4	0.4	0.4	0.4	0.4	0.4	0.4	0.4	0.4	0.4	0.4	0.4	0.4	0.4	0.4	0.4	0.4	0.4
		M00621	Glycine cleavage system	1	1	1	1	1	1	1	1	1	1	1	1	1	1	1	1	1	1	1	1	1	1	1	1	1	1	1	1	1	1	1	1	1	1	1	1	1
	Cysteine and methionine metabolism	M00017	Methionine biosynthesis, aspartate => homoserine => methionine	0.571429	0.571429	0.571429	0.714286	0.714286	0.714286	0.714286	0.714286	0.714286	0.714286	0.714286	0.714286	0.714286	0.571429	0.571429	0.571429	0.571429	0.571429	0.571429	0.571429	0.571429	0.571429	0.714286	0.714286	0.714286	0.714286	0.714286	0.714286	0.714286	0.714286	0.714286	0.714286	0.714286	0.714286	0.714286	0.714286	0.714286
		M00021	Cysteine biosynthesis, serine => cysteine	1	1	1	1	1	1	1	1	1	1	1	1	1	1	1	1	1	1	1	1	1	1	1	1	1	1	1	1	1	1	1	1	1	1	1	1	1
		M00034	Methionine salvage pathway	0.375	0.375	0.375	0.375	0.375	0.375	0.375	0.375	0.375	0.375	0.375	0.375	0.375	0.375	0.375	0.375	0.375	0.375	0.375	0.375	0.375	0.375	0.25	0.375	0.375	0.375	0.375	0.375	0.375	0.375	0.375	0.375	0.375	0.375	0.375	0.375	0.375
		M00035	Methionine degradation	0.75	0.75	0.75	0.75	0.75	0.75	0.75	0.75	0.75	0.75	0.75	0.75	0.75	0.75	0.75	0.75	0.75	0.75	0.75	0.75	0.75	0.75	0.5	0.75	0.75	0.75	0.75	0.75	0.75	0.75	0.75	0.75	0.75	0.75	0.75	0.75	0.75
		M00368	Ethylene biosynthesis, methionine => ethylene	0.333333	0.333333	0.333333	0.333333	0.333333	0.333333	0.333333	0.333333	0.333333	0.333333	0.333333	0.333333	0.333333	0.333333	0.333333	0.333333	0.333333	0.333333	0.333333	0.333333	0.333333	0.333333	0	0.333333	0.333333	0.333333	0.333333	0.333333	0.333333	0.333333	0.333333	0.333333	0.333333	0.333333	0.333333	0.333333	0.333333
		M00609	Cysteine biosynthesis, methionine => cysteine	0.166667	0.166667	0.166667	0.166667	0.166667	0.166667	0.166667	0.166667	0.166667	0.166667	0.166667	0.166667	0.166667	0.166667	0.166667	0.166667	0.166667	0.166667	0.166667	0.166667	0.166667	0.166667	0	0.166667	0.166667	0.166667	0.166667	0.166667	0.166667	0.166667	0.166667	0.166667	0.166667	0.166667	0.166667	0.166667	0.166667
	Branched-chain amino acid metabolism	M00019	Valine/isoleucine biosynthesis, pyruvate => valine / 2-oxobutanoate => isoleucine	0.75	0.75	0.75	0.75	0.75	0.75	0.75	0.75	0.75	0.75	0.75	0.75	0.75	0.75	0.75	0.75	0.75	0.75	0.75	0.75	0.75	0.75	0.75	0.75	0.75	0.75	0.75	0.75	0.75	0.75	0.75	0.75	0.75	0.75	0.75	0.75	0.75
		M00036	Leucine degradation, leucine => acetoacetate + acetyl-CoA	0.055556	0.055556	0.055556	0.055556	0.055556	0.055556	0.055556	0.055556	0.055556	0.055556	0.055556	0.055556	0.055556	0.055556	0.055556	0.055556	0.055556	0.055556	0.055556	0.055556	0.055556	0.055556	0.055556	0.055556	0.055556	0.055556	0.055556	0.055556	0.055556	0.055556	0.055556	0.055556	0.055556	0.055556	0.055556	0.055556	0.055556
		M00432	Leucine biosynthesis, 2-oxoisovalerate => 2-oxoisocaproate	1	1	1	1	1	1	0.666667	1	1	1	1	1	1	1	1	1	1	1	1	1	1	1	1	1	1	1	1	1	1	1	1	1	1	1	1	1	1
		M00535	Isoleucine biosynthesis, pyruvate => 2-oxobutanoate	0.666667	0.666667	0.666667	0.666667	0.666667	0.666667	0.333333	0.666667	0.666667	0.666667	0.666667	0.666667	0.666667	0.666667	0.666667	0.666667	0.666667	0.666667	0.666667	0.666667	0.666667	0.666667	0.666667	0.666667	0.666667	0.666667	0.666667	0.666667	0.666667	0.666667	0.666667	0.666667	0.666667	0.666667	0.666667	0.666667	0.666667
		M00570	Isoleucine biosynthesis, threonine => 2-oxobutanoate => isoleucine	0.8	0.8	0.8	0.8	0.8	0.8	0.6	0.8	0.8	0.8	0.8	0.8	0.8	0.8	0.8	0.8	0.8	0.8	0.8	0.8	0.8	0.8	0.8	0.8	0.8	0.8	0.8	0.8	0.8	0.8	0.8	0.8	0.8	0.8	0.8	0.8	0.8
	Lysine metabolism	M00016	Lysine biosynthesis, succinyl-DAP pathway, aspartate => lysine	0.777778	0.777778	0.777778	0.777778	0.777778	0.777778	0.777778	0.777778	0.777778	0.777778	0.777778	0.777778	0.777778	0.777778	0.777778	0.777778	0.777778	0.777778	0.777778	0.777778	0.777778	0.777778	0.777778	0.777778	0.777778	0.777778	0.777778	0.777778	0.777778	0.777778	0.777778	0.777778	0.777778	0.777778	0.777778	0.777778	0.777778

		M00032	Lysine degradation, lysine => saccharopine => acetoacetyl-CoA	0.055556	0.055556	0.055556	0.055556	0.055556	0.055556	0.055556	0.055556	0.055556	0.055556	0.055556	0.055556	0.055556	0.055556	0.055556	0.055556	0.055556	0.055556	0.055556	0.055556	0.055556	0.055556	0.055556	0.055556	0.055556	0.055556	0.055556	0.055556	0.055556	0.055556	0.055556	0.055556	0.055556	0.055556	0.055556	0.055556	0.055556
		M00525	Lysine biosynthesis, acetyl-DAP pathway, aspartate => lysine	0.666667	0.666667	0.666667	0.777778	0.777778	0.666667	0.666667	0.666667	0.666667	0.666667	0.666667	0.666667	0.666667	0.666667	0.666667	0.666667	0.666667	0.666667	0.666667	0.666667	0.666667	0.666667	0.666667	0.666667	0.666667	0.666667	0.666667	0.666667	0.666667	0.666667	0.666667	0.666667	0.666667	0.666667	0.666667	0.666667	0.666667
		M00526	Lysine biosynthesis, DAP dehydrogenase pathway, aspartate => lysine	0.833333	0.833333	0.833333	0.833333	0.833333	0.833333	0.833333	0.833333	0.833333	0.833333	0.833333	0.833333	0.833333	0.833333	0.833333	0.833333	0.833333	0.833333	0.833333	0.833333	0.833333	0.833333	0.833333	0.833333	0.833333	0.833333	0.833333	0.833333	0.833333	0.833333	0.833333	0.833333	0.833333	0.833333	0.833333	0.833333	0.833333
		M00527	Lysine biosynthesis, DAP aminotransferase pathway, aspartate => lysine	1	1	1	1	1	1	1	1	1	1	1	1	1	1	1	1	1	1	1	1	1	1	1	1	1	1	1	1	1	1	1	1	1	1	1	1	1
		M00956	Lysine degradation, bacteria, L-lysine => succinate	0.285714	0.285714	0.285714	0.142857	0.285714	0.285714	0.285714	0.285714	0.285714	0.285714	0.285714	0.285714	0.285714	0.285714	0.285714	0.285714	0.285714	0.285714	0.285714	0.285714	0.285714	0.285714	0.285714	0.285714	0.285714	0.285714	0.285714	0.285714	0.285714	0.285714	0.285714	0.285714	0.285714	0.285714	0.285714	0.285714	0.285714
		M00957	Lysine degradation, bacteria, L-lysine => glutarate => succinate/acetyl-CoA	0.333333	0.333333	0.333333	0.166667	0.333333	0.333333	0.333333	0.333333	0.333333	0.333333	0.333333	0.333333	0.333333	0.333333	0.333333	0.333333	0.333333	0.333333	0.333333	0.333333	0.333333	0.333333	0.333333	0.333333	0.333333	0.333333	0.333333	0.333333	0.333333	0.333333	0.333333	0.333333	0.333333	0.333333	0.333333	0.333333	0.333333
	Arginine and proline metabolism	M00015	Proline biosynthesis, glutamate => proline	1	1	1	1	1	1	1	1	1	1	1	1	1	1	1	1	1	1	1	1	1	1	1	1	1	1	0.666667	1	1	1	1	1	1	1	1	1	1
		M00028	Ornithine biosynthesis, glutamate => ornithine	1	1	1	1	1	1	1	1	1	1	1	1	1	1	1	1	1	1	1	0.8	1	1	1	1	1	1	1	1	1	1	1	1	1	1	1	1	1
		M00029	Urea cycle	0.6	0.6	0.6	0.6	0.6	0.6	0	0.6	0.6	0.6	0.6	0.6	0.6	0.6	0.6	0.6	0.6	0.6	0.6	0.6	0.6	0.6	0.6	0.6	0.6	0.6	0.6	0.6	0.6	0.6	0.6	0.6	0.6	0.6	0.6	0.6	0.6
		M00047	Creatine pathway	0	0	0.333333	0.333333	0	0.333333	0	0.333333	0.333333	0.333333	0.333333	0	0	0.333333	0	0.333333	0.333333	0	0	0	0	0	0	0	0	0	0.333333	0.333333	0.333333	0.333333	0.333333	0.333333	0.333333	0.333333	0.333333	0.333333	0.333333
		M00844	Arginine biosynthesis, ornithine => arginine	1	1	1	1	1	1	0	1	1	1	1	1	1	1	1	1	1	1	1	1	1	1	1	1	1	1	1	1	1	1	1	1	1	1	1	1	1
		M00845	Arginine biosynthesis, glutamate => acetylcitrulline => arginine	0.571429	0.571429	0.571429	0.571429	0.571429	0.571429	0.285714	0.571429	0.571429	0.571429	0.571429	0.571429	0.571429	0.571429	0.571429	0.571429	0.571429	0.571429	0.571429	0.428571	0.571429	0.571429	0.571429	0.571429	0.571429	0.571429	0.571429	0.571429	0.571429	0.571429	0.571429	0.571429	0.571429	0.571429	0.571429	0.571429	0.571429
		M00970	Proline degradation, proline => glutamate	1	1	1	0.5	1	1	1	1	1	1	1	1	1	1	1	1	1	1	1	1	1	1	1	1	1	1	1	1	1	1	1	1	1	1	1	1	1
		M00972	Proline metabolism	0.666667	0.666667	0.666667	0.666667	0.666667	0.666667	0.666667	0.666667	0.666667	0.666667	0.666667	0.666667	0.666667	0.666667	0.666667	0.666667	0.666667	0.666667	0.666667	0.666667	0.666667	0.666667	0.666667	0.666667	0.666667	0.666667	0.333333	0.666667	0.666667	0.666667	0.666667	0.666667	0.666667	0.666667	0.666667	0.666667	0.666667
	Polyamine biosynthesis	M00133	Polyamine biosynthesis, arginine => agmatine => putrescine => spermidine	0.5	0.5	0.5	0.5	0.5	0.5	0.5	0.5	0.5	0.5	0.5	0.5	0.5	0.5	0.5	0.5	0.5	0.5	0.5	0.5	0.5	0.5	0.5	0.5	0.5	0.5	0.5	0.5	0.5	0.5	0.5	0.5	0.5	0.5	0.5	0.5	0.5
	Histidine metabolism	M00026	Histidine biosynthesis, PRPP => histidine	0.75	0.875	0.875	0.875	0.875	0.875	0.875	0.875	0.875	0.875	0.75	0.875	0.875	0.875	0.875	0.875	0.875	0.875	0.875	0.875	0.875	0.875	0.875	0.875	0.875	0.875	0.875	0.875	0.875	0.875	0.875	0.875	0.875	0.875	0.875	0.875	0.875
	Aromatic amino acid metabolism	M00022	Shikimate pathway, phosphoenolpyruvate + erythrose-4P => chorismate	1	1	1	1	1	1	1	1	1	1	1	1	1	1	1	1	1	1	1	1	1	1	1	1	1	1	1	1	1	1	1	1	1	1	1	1	1
		M00023	Tryptophan biosynthesis, chorismate => tryptophan	1	1	1	1	1	1	1	1	1	1	1	1	1	1	1	1	1	1	1	1	1	0.9	1	0.9	1	1	0.9	1	1	1	1	1	1	1	1	1	1
		M00024	Phenylalanine biosynthesis, chorismate => phenylpyruvate => phenylalanine	0.666667	0.666667	0.666667	0.666667	0.666667	0.666667	0.666667	0.666667	0.666667	0.666667	0.333333	0.666667	0.666667	0.666667	0.666667	0.666667	0.666667	0.666667	0.666667	0.666667	0.666667	0.666667	0.666667	0.666667	0.666667	0.666667	0.666667	0.666667	0.666667	0.666667	0.666667	0.666667	0.666667	0.666667	0.666667	0.666667	0.666667
		M00025	Tyrosine biosynthesis, chorismate => HPP => tyrosine	0.666667	0.666667	0.666667	0.666667	0.666667	0.666667	0.666667	0.666667	0.666667	0.666667	0.666667	0.666667	0.666667	0.666667	0.666667	0.666667	0.666667	0.666667	0.666667	0.666667	0.666667	0.666667	0.666667	0.666667	0.666667	0.666667	0.666667	0.666667	0.666667	0.666667	0.666667	0.666667	0.666667	0.666667	0.666667	0.666667	0.666667
		M00038	Tryptophan metabolism, tryptophan => kynurenine => 2-aminomuconate	0.142857	0	0.142857	0	0	0	0	0	0	0	0	0	0	0	0.142857	0	0	0	0	0	0.142857	0.142857	0	0.142857	0.142857	0.142857	0	0	0	0	0	0	0	0	0	0	0
		M00044	Tyrosine degradation, tyrosine => homogentisate	0.2	0.2	0.2	0.2	0.2	0.2	0.2	0.2	0.2	0.2	0.2	0.2	0.2	0.2	0.2	0.2	0.2	0.2	0.2	0.2	0.2	0.2	0.2	0.2	0.2	0.2	0.2	0.2	0.2	0.2	0.2	0.2	0.2	0.2	0.2	0.2	0.2
		M00936	Melatonin biosynthesis, plants, tryptophan => serotonin => melatonin	0.25	0.25	0.25	0.25	0.25	0.25	0.25	0.25	0.25	0.25	0.25	0.25	0.25	0.25	0.25	0.25	0.25	0.25	0.25	0.25	0.25	0.25	0.25	0.25	0.25	0.25	0.25	0.25	0.25	0.25	0.25	0.25	0.25	0.25	0.25	0.25	0.25
	Other amino acid metabolism	M00027	GABA (gamma-Aminobutyrate) shunt	0.333333	0.333333	0.333333	0	0.333333	0.333333	0.333333	0.333333	0.333333	0.333333	0.333333	0.333333	0.333333	0.333333	0.333333	0.333333	0.333333	0.333333	0.333333	0.333333	0.333333	0.333333	0.333333	0.333333	0.333333	0.333333	0.333333	0.333333	0.333333	0.333333	0.333333	0.333333	0.333333	0.333333	0.333333	0.333333	0.333333
		M00118	Glutathione biosynthesis, glutamate => glutathione	0.5	0.5	0.5	0.5	0.5	0.5	0.5	0.5	0.5	0.5	0.5	0.5	0.5	0.5	0.5	0.5	0.5	0.5	0.5	0.5	0.5	0.5	0.5	0.5	0.5	0.5	0.5	0.5	0.5	0.5	0.5	0.5	0.5	0.5	0.5	0.5	0.5
Glycan metabolism	Glycan biosynthesis	M00872	O-glycan biosynthesis, mannose type (core M3)	0.1	0.1	0.1	0.1	0.1	0.1	0.1	0.1	0.1	0.1	0.1	0.1	0.1	0.1	0.1	0.1	0.1	0.1	0.1	0.1	0.1	0.1	0.1	0.1	0.1	0.1	0.1	0.1	0.1	0.1	0.1	0.1	0.1	0.1	0.1	0.1	0.1
	Glycosaminoglycan metabolism	M00078	Heparan sulfate degradation	0.111111	0.111111	0.111111	0.111111	0.111111	0.111111	0.111111	0.111111	0.111111	0.111111	0.111111	0.111111	0.111111	0.111111	0.111111	0.111111	0.111111	0.111111	0.111111	0.111111	0.111111	0.111111	0.111111	0.111111	0.111111	0.111111	0.111111	0.111111	0.111111	0.111111	0.111111	0.111111	0.111111	0.111111	0.111111	0.111111	0.111111
	Lipopolysaccharide metabolism	M00060	KDO2-lipid A biosynthesis, Raetz pathway, LpxL-LpxM type	0.444444	0.444444	0.444444	0.444444	0.444444	0.444444	0.333333	0.444444	0.444444	0.444444	0.444444	0.444444	0.444444	0.444444	0.444444	0.444444	0.444444	0.444444	0.444444	0.444444	0.444444	0.444444	0.444444	0.444444	0.444444	0.444444	0.444444	0.444444	0.444444	0.444444	0.444444	0.444444	0.444444	0.444444	0.444444	0.444444	0.444444
		M00063	CMP-KDO biosynthesis	0	0	0	0.25	0	0	0	0	0.25	0	0	0	0	0	0	0	0	0.25	0	0	0	1	0	0	0	0	0	0	0	0	0	0	0.25	0.25	0.25	0.25	0.25
		M00064	ADP-L-glycero-D-manno-heptose biosynthesis	0.2	0.4	0.2	0.2	0.2	0	0	0.2	0	0.4	0.4	0.4	0	0.2	0	1	0	1	0	0.2	0.2	0	0.2	0.2	0.2	0.2	0.2	0	0.2	0.2	0.2	0	0	0	0	0	0
		M00866	KDO2-lipid A biosynthesis, Raetz pathway, non-LpxL-LpxM type	0.444444	0.444444	0.444444	0.444444	0.444444	0.444444	0.333333	0.444444	0.444444	0.444444	0.444444	0.444444	0.444444	0.444444	0.444444	0.444444	0.444444	0.444444	0.444444	0.444444	0.444444	0.444444	0.444444	0.444444	0.444444	0.444444	0.444444	0.444444	0.444444	0.444444	0.444444	0.444444	0.444444	0.444444	0.444444	0.444444	0.444444
		M00867	KDO2-lipid A modification pathway	0	0	0	0	0.2	0	0	0	0	0.2	0	0	0	0	0	0	0	0	0	0	0	0	0	0	0	0	0	0	0	0	0	0	0	0	0	0	0
		M00922	CMP-Neu5Ac biosynthesis	0	0	0	0	0	0	0	0	0	0	0	0	0	0.333333	0	0	0	0	0	0	0	0	0	0	0	0	0	0	0	0	0	0	0	0	0	0	0
		M00923	UDP-L-FucNAm biosynthesis	0	0.25	0.25	0	0.25	0	0.25	0.25	0	0	0	0	0	0	0	0	0	0	0	0	0.25	0	0.25	0	0	0	0.25	0.25	0.25	0.25	0.25	0	0	0	0	0	0
Metabolism of cofactors and vitamins	Cofactor and vitamin metabolism	M00112	Tocopherol/tocotorienol biosynthesis, homogentisate + phytyl/geranylgeranyl-PP => tocopherol/tocotorienol	0.75	0.75	0.75	0.75	0.75	0.75	0.75	0.75	0.75	0.75	0.75	0.75	0.75	0.75	0.75	0.75	0.75	0.75	0.75	0.75	0.75	0.75	0.75	0.75	0.75	0.75	0.75	0.75	0.75	0.75	0.75	0.75	0.75	0.75	0.75	0.75	0.75
		M00115	NAD biosynthesis, aspartate => quinolinate => NAD	1	1	1	0.8	1	1	1	1	1	1	0.8	1	1	1	1	1	1	1	1	1	1	1	1	1	1	1	1	1	1	1	1	1	1	1	1	1	1
		M00116	Menaquinone biosynthesis, chorismate (+ polyprenyl-PP) => menaquinol	0.555556	0.666667	0.666667	0.666667	0.666667	0.666667	0.555556	0.666667	0.666667	0.666667	0.666667	0.666667	0.666667	0.666667	0.666667	0.666667	0.666667	0.666667	0.666667	0.666667	0.666667	0.666667	0.666667	0.666667	0.666667	0.666667	0.666667	0.666667	0.666667	0.666667	0.666667	0.555556	0.666667	0.666667	0.666667	0.666667	0.666667
		M00117	Ubiquinone biosynthesis, prokaryotes, chorismate (+ polyprenyl-PP) => ubiquinol	0.444444	0.444444	0.444444	0.444444	0.444444	0.444444	0.333333	0.444444	0.444444	0.444444	0.444444	0.444444	0.444444	0.444444	0.444444	0.444444	0.444444	0.444444	0.444444	0.444444	0.444444	0.444444	0.444444	0.444444	0.444444	0.444444	0.444444	0.444444	0.444444	0.444444	0.444444	0.444444	0.444444	0.444444	0.444444	0.444444	0.444444
		M00119	Pantothenate biosynthesis, valine/L-aspartate => pantothenate	0.6	0.6	0.6	0.6	0.6	0.6	0.6	0.6	0.6	0.6	0.6	0.6	0.6	0.6	0.6	0.6	0.6	0.6	0.6	0.6	0.6	0.6	0.6	0.6	0.6	0.6	0.6	0.6	0.6	0.6	0.6	0.6	0.6	0.6	0.6	0.6	0.6
		M00120	Coenzyme A biosynthesis, pantothenate => CoA	1	1	1	1	1	1	1	1	1	1	0.75	1	1	1	1	1	1	1	1	1	1	1	1	1	1	1	1	1	1	1	1	1	1	1	1	1	1
		M00121	Heme biosynthesis, plants and bacteria, glutamate => heme	1	1	1	1	1	1	1	1	1	1	1	1	1	1	0.9	1	1	1	1	1	1	1	1	1	1	1	1	1	1	1	1	1	1	1	1	1	1
		M00122	Cobalamin biosynthesis, cobyrinate a,c-diamide => cobalamin	0.714286	0.714286	0.714286	0.714286	0.714286	0.714286	0.714286	0.714286	0.714286	0.714286	0.714286	0.714286	0.714286	0.714286	0.714286	0.714286	0.714286	0.714286	0.714286	0.714286	0.714286	0.714286	0.714286	0.714286	0.714286	0.714286	0.714286	0.714286	0.714286	0.714286	0.714286	0.714286	0.714286	0.714286	0.714286	0.714286	0.714286
		M00123	Biotin biosynthesis, pimeloyl-ACP/CoA => biotin	0.75	0.75	0.75	0.5	0.75	0.75	0.75	0.75	0.75	0.75	0.75	0.75	0.75	0.75	0.75	0.75	0.75	0.75	0.75	0.75	0.75	0.75	0.75	0.75	0.75	0.75	0.75	0.75	0.75	0.75	0.75	0.75	0.75	0.75	0.75	0.75	0.75
		M00124	Pyridoxal-P biosynthesis, erythrose-4P => pyridoxal-P	0.333333	0.333333	0.333333	0.333333	0.333333	0.333333	0.333333	0.333333	0.333333	0.333333	0.166667	0.333333	0.333333	0.333333	0.333333	0.333333	0.333333	0.333333	0.333333	0.333333	0.333333	0.333333	0.333333	0.333333	0.333333	0.333333	0.333333	0.333333	0.333333	0.333333	0.333333	0.333333	0.333333	0.333333	0.333333	0.333333	0.333333
		M00125	Riboflavin biosynthesis, plants and bacteria, GTP => riboflavin/FMN/FAD	1	1	1	1	1	1	1	1	1	1	1	1	1	1	1	1	1	1	1	0.714286	1	1	1	1	1	1	1	1	1	1	1	1	1	1	1	1	1
		M00126	Tetrahydrofolate biosynthesis, GTP => THF	1	1	1	0.857143	1	1	1	1	1	1	1	1	1	1	1	1	1	1	1	1	1	1	1	1	0.857143	1	1	1	1	1	1	1	1	1	1	1	1
		M00127	Thiamine biosynthesis, prokaryotes, AIR (+ DXP/tyrosine) => TMP/TPP	0.5625	0.5625	0.5625	0.5625	0.5625	0.5625	0.5625	0.5625	0.5625	0.5625	0.5625	0.5625	0.5625	0.5625	0.5625	0.5625	0.5625	0.5625	0.5625	0.5625	0.5625	0.5625	0.5625	0.5625	0.5625	0.5625	0.4375	0.5625	0.5625	0.5625	0.5625	0.5625	0.5625	0.5625	0.5625	0.5625	0.5625
		M00140	C1-unit interconversion, prokaryotes	0.666667	0.666667	0.666667	0.666667	0.666667	0.666667	0.666667	0.666667	0.666667	0.666667	0.666667	0.666667	0.666667	0.666667	0.666667	0.666667	0.666667	0.666667	0.666667	0.666667	0.666667	0.666667	0.666667	0.666667	0.666667	0.666667	0.666667	0.666667	0.666667	0.666667	0.666667	0.666667	0.666667	0.666667	0.666667	0.666667	0.666667
		M00141	C1-unit interconversion, eukaryotes	0.5	0.5	0.5	0.5	0.5	0.5	0.5	0.5	0.5	0.5	0.5	0.5	0.5	0.5	0.5	0.5	0.5	0.5	0.5	0.5	0.5	0.5	0.5	0.5	0.5	0.5	0.5	0.5	0.5	0.5	0.5	0.5	0.5	0.5	0.5	0.5	0.5
		M00572	Pimeloyl-ACP biosynthesis, BioC-BioH pathway, malonyl-ACP => pimeloyl-ACP	0.666667	0.666667	0.666667	0.666667	0.666667	0.666667	0.666667	0.666667	0.666667	0.666667	0.666667	0.666667	0.666667	0.666667	0.666667	0.666667	0.666667	0.666667	0.666667	0.666667	0.666667	0.666667	0.666667	0.666667	0.666667	0.666667	0.666667	0.666667	0.666667	0.666667	0.666667	0.666667	0.666667	0.666667	0.666667	0.666667	0.666667
		M00573	Biotin biosynthesis, BioI pathway, long-chain-acyl-ACP => pimeloyl-ACP => biotin	0.6	0.6	0.6	0.4	0.6	0.6	0.6	0.6	0.6	0.6	0.6	0.6	0.6	0.6	0.6	0.6	0.6	0.6	0.6	0.6	0.6	0.6	0.6	0.6	0.6	0.6	0.6	0.6	0.6	0.6	0.6	0.6	0.6	0.6	0.6	0.6	0.6
		M00577	Biotin biosynthesis, BioW pathway, pimelate => pimeloyl-CoA => biotin	0.6	0.6	0.6	0.4	0.6	0.6	0.6	0.6	0.6	0.6	0.6	0.6	0.6	0.6	0.6	0.6	0.6	0.6	0.6	0.6	0.6	0.6	0.6	0.6	0.6	0.6	0.6	0.6	0.6	0.6	0.6	0.6	0.6	0.6	0.6	0.6	0.6
		M00840	Tetrahydrofolate biosynthesis, mediated by ribA and trpF, GTP => THF	0.428571	0.428571	0.428571	0.428571	0.428571	0.428571	0.428571	0.428571	0.428571	0.428571	0.428571	0.428571	0.428571	0.428571	0.428571	0.428571	0.428571	0.428571	0.428571	0.285714	0.428571	0.428571	0.428571	0.428571	0.285714	0.428571	0.428571	0.428571	0.428571	0.428571	0.428571	0.428571	0.428571	0.428571	0.428571	0.428571	0.428571
		M00841	Tetrahydrofolate biosynthesis, mediated by PTPS, GTP => THF	0.4	0.4	0.4	0.4	0.4	0.4	0.4	0.4	0.4	0.4	0.4	0.4	0.4	0.4	0.4	0.4	0.4	0.4	0.4	0.4	0.4	0.4	0.4	0.4	0.4	0.4	0.4	0.4	0.4	0.4	0.4	0.4	0.4	0.4	0.4	0.4	0.4
		M00842	Tetrahydrobiopterin biosynthesis, GTP => BH4	0.666667	0.666667	0.666667	0.666667	0.666667	0.666667	0.666667	0.666667	0.666667	0.666667	0.666667	0.666667	0.666667	0.666667	0.666667	0.666667	0.666667	0.666667	0.666667	0.666667	0.666667	0.666667	0.666667	0.666667	0.666667	0.666667	0.666667	0.666667	0.666667	0.666667	0.666667	0.666667	0.666667	0.666667	0.666667	0.666667	0.666667
		M00843	L-threo-Tetrahydrobiopterin biosynthesis, GTP => L-threo-BH4	0.666667	0.666667	0.666667	0.666667	0.666667	0.666667	0.666667	0.666667	0.666667	0.666667	0.666667	0.666667	0.666667	0.666667	0.666667	0.666667	0.666667	0.666667	0.666667	0.666667	0.666667	0.666667	0.666667	0.666667	0.666667	0.666667	0.666667	0.666667	0.666667	0.666667	0.666667	0.666667	0.666667	0.666667	0.666667	0.666667	0.666667
		M00846	Siroheme biosynthesis, glutamyl-tRNA => siroheme	0.916667	0.916667	0.916667	0.916667	0.916667	0.916667	0.916667	0.916667	0.916667	0.916667	0.916667	0.916667	0.916667	0.916667	0.75	0.916667	0.916667	0.916667	0.916667	0.916667	0.916667	0.916667	0.916667	0.916667	0.916667	0.916667	0.916667	0.916667	0.916667	0.916667	0.916667	0.916667	0.916667	0.916667	0.916667	0.916667	0.916667
		M00868	Heme biosynthesis, animals and fungi, glycine => heme	0.75	0.75	0.75	0.75	0.75	0.75	0.75	0.75	0.75	0.75	0.75	0.75	0.75	0.75	0.625	0.75	0.75	0.75	0.75	0.75	0.75	0.75	0.75	0.75	0.75	0.75	0.75	0.75	0.75	0.75	0.75	0.75	0.75	0.75	0.75	0.75	0.75
		M00880	Molybdenum cofactor biosynthesis, GTP => molybdenum cofactor	1	1	1	1	1	1	1	1	1	1	1	1	1	1	1	1	1	1	1	0.8	1	1	1	1	1	1	1	1	1	1	1	1	1	1	1	1	1
		M00881	Lipoic acid biosynthesis, plants and bacteria, octanoyl-ACP => dihydrolipoyl-E2/H	1	1	1	1	1	1	1	1	1	1	1	1	1	1	1	1	1	1	1	1	1	1	1	1	1	1	1	1	1	1	1	1	1	1	1	1	1
		M00882	Lipoic acid biosynthesis, eukaryotes, octanoyl-ACP => dihydrolipoyl-H	0.5	0.5	0.5	0.5	0.5	0.5	0.5	0.5	0.5	0.5	0.5	0.5	0.5	0.5	0.5	0.5	0.5	0.5	0.5	0.5	0.5	0.5	0.5	0.5	0.5	0.5	0.5	0.5	0.5	0.5	0.5	0.5	0.5	0.5	0.5	0.5	0.5
		M00883	Lipoic acid biosynthesis, animals and bacteria, octanoyl-ACP => dihydrolipoyl-H => dihydrolipoyl-E2	0.333333	0.333333	0.333333	0.333333	0.333333	0.333333	0.333333	0.333333	0.333333	0.333333	0.333333	0.333333	0.333333	0.333333	0.333333	0.333333	0.333333	0.333333	0.333333	0.333333	0.333333	0.333333	0.333333	0.333333	0.333333	0.333333	0.333333	0.333333	0.333333	0.333333	0.333333	0.333333	0.333333	0.333333	0.333333	0.333333	0.333333
		M00884	Lipoic acid biosynthesis, octanoyl-CoA => dihydrolipoyl-E2	0.5	0.5	0.5	0.5	0.5	0.5	0.5	0.5	0.5	0.5	0.5	0.5	0.5	0.5	0.5	0.5	0.5	0.5	0.5	0.5	0.5	0.5	0.5	0.5	0.5	0.5	0.5	0.5	0.5	0.5	0.5	0.5	0.5	0.5	0.5	0.5	0.5
		M00895	Thiamine biosynthesis, prokaryotes, AIR (+ DXP/glycine) => TMP/TPP	0.611111	0.611111	0.611111	0.611111	0.611111	0.611111	0.611111	0.611111	0.611111	0.611111	0.611111	0.611111	0.611111	0.611111	0.611111	0.611111	0.611111	0.611111	0.611111	0.611111	0.611111	0.611111	0.611111	0.611111	0.611111	0.611111	0.5	0.611111	0.611111	0.611111	0.611111	0.611111	0.611111	0.611111	0.611111	0.611111	0.611111
		M00896	Thiamine biosynthesis, archaea, AIR (+ NAD+) => TMP/TPP	0.6	0.6	0.6	0.6	0.6	0.6	0.6	0.6	0.6	0.6	0.6	0.6	0.6	0.6	0.6	0.6	0.6	0.6	0.6	0.6	0.6	0.6	0.6	0.6	0.6	0.6	0.4	0.6	0.6	0.6	0.6	0.6	0.6	0.6	0.6	0.6	0.6
		M00897	Thiamine biosynthesis, plants, AIR (+ NAD+) => TMP/thiamine/TPP	0.2	0.2	0.2	0.2	0.2	0.2	0.2	0.2	0.2	0.2	0.2	0.2	0.2	0.2	0.2	0.2	0.2	0.2	0.2	0.2	0.2	0.2	0.2	0.2	0.2	0.2	0.2	0.2	0.2	0.2	0.2	0.2	0.2	0.2	0.2	0.2	0.2
		M00899	Thiamine salvage pathway, HMP/HET => TMP	0.333333	0.333333	0.333333	0.333333	0.333333	0.333333	0.333333	0.333333	0.333333	0.333333	0.333333	0.333333	0.333333	0.333333	0.333333	0.333333	0.333333	0.333333	0.333333	0.333333	0.333333	0.333333	0.333333	0.333333	0.333333	0.333333	0.333333	0.333333	0.333333	0.333333	0.333333	0.333333	0.333333	0.333333	0.333333	0.333333	0.333333
		M00911	Riboflavin biosynthesis, fungi, GTP => riboflavin/FMN/FAD	0.222222	0.222222	0.222222	0.222222	0.222222	0.222222	0.222222	0.222222	0.222222	0.222222	0.222222	0.222222	0.222222	0.222222	0.222222	0.222222	0.222222	0.222222	0.222222	0.222222	0.222222	0.222222	0.222222	0.222222	0.222222	0.222222	0.222222	0.222222	0.222222	0.222222	0.222222	0.222222	0.222222	0.222222	0.222222	0.222222	0.222222
		M00912	NAD biosynthesis, tryptophan => quinolinate => NAD	0.5	0.375	0.5	0.25	0.375	0.375	0.375	0.375	0.375	0.375	0.25	0.375	0.375	0.375	0.5	0.375	0.375	0.375	0.375	0.375	0.5	0.5	0.375	0.5	0.5	0.5	0.375	0.375	0.375	0.375	0.375	0.375	0.375	0.375	0.375	0.375	0.375
		M00913	Pantothenate biosynthesis, 2-oxoisovalerate/spermine => pantothenate	0.4	0.4	0.4	0.4	0.4	0.4	0.4	0.4	0.4	0.4	0.4	0.4	0.4	0.4	0.4	0.4	0.4	0.4	0.4	0.4	0.4	0.4	0.4	0.4	0.4	0.4	0.4	0.4	0.4	0.4	0.4	0.4	0.4	0.4	0.4	0.4	0.4
		M00914	Coenzyme A biosynthesis, archaea, 2-oxoisovalerate => 4-phosphopantoate => CoA	0.285714	0.285714	0.285714	0.285714	0.285714	0.285714	0.285714	0.285714	0.285714	0.285714	0.285714	0.142857	0.285714	0.285714	0.285714	0.285714	0.285714	0.285714	0.285714	0.285714	0.285714	0.285714	0.285714	0.285714	0.285714	0.285714	0.285714	0.285714	0.285714	0.285714	0.285714	0.285714	0.285714	0.285714	0.285714	0.285714	0.285714
		M00924	Cobalamin biosynthesis, anaerobic, uroporphyrinogen III => sirohydrochlorin => cobyrinate a,c-diamide	0.916667	0.916667	0.916667	0.833333	0.916667	0.916667	0.916667	0.916667	0.916667	0.916667	0.916667	0.916667	0.916667	0.916667	0.916667	0.916667	0.916667	0.916667	0.916667	0.916667	0.916667	0.916667	0.916667	0.916667	0.916667	0.916667	0.916667	0.916667	0.916667	0.916667	0.916667	0.916667	0.916667	0.916667	0.916667	0.916667	0.916667
		M00925	Cobalamin biosynthesis, aerobic, uroporphyrinogen III => precorrin 2 => cobyrinate a,c-diamide	0.848485	0.848485	0.848485	0.757576	0.848485	0.848485	0.848485	0.848485	0.848485	0.848485	0.848485	0.848485	0.848485	0.848485	0.848485	0.848485	0.848485	0.848485	0.848485	0.848485	0.848485	0.848485	0.848485	0.848485	0.848485	0.848485	0.848485	0.848485	0.848485	0.848485	0.848485	0.848485	0.848485	0.848485	0.757576	0.848485	0.848485
		M00926	Heme biosynthesis, bacteria, glutamyl-tRNA => coproporphyrin III => heme	0.777778	0.777778	0.777778	0.777778	0.777778	0.777778	0.777778	0.777778	0.777778	0.777778	0.777778	0.777778	0.777778	0.777778	0.777778	0.666667	0.777778	0.777778	0.777778	0.777778	0.777778	0.777778	0.777778	0.777778	0.777778	0.777778	0.777778	0.777778	0.777778	0.777778	0.777778	0.777778	0.777778	0.777778	0.777778	0.777778	0.777778
		M00932	Phylloquinone biosynthesis, chorismate (+ phytyl-PP) => phylloquinol	0.9	1	1	1	1	1	0.8	1	1	1	1	1	1	1	1	1	1	1	1	1	1	1	1	1	1	1	1	1	1	1	1	1	0.9	1	1	1	1
		M00950	Biotin biosynthesis, BioU pathway, pimeloyl-ACP/CoA => biotin	1	1	1	0.5	1	1	1	1	1	1	1	1	1	1	1	1	1	1	1	1	1	1	1	1	1	1	1	1	1	1	1	1	1	1	1	1	1
Biosynthesis of terpenoids and polyketides	Terpenoid backbone biosynthesis	M00095	C5 isoprenoid biosynthesis, mevalonate pathway	0.142857	0.142857	0.142857	0.142857	0.142857	0.142857	0.142857	0.142857	0.142857	0.142857	0.142857	0.142857	0.142857	0.142857	0.142857	0.142857	0.142857	0.142857	0.142857	0.142857	0.142857	0.142857	0.142857	0.142857	0.142857	0.142857	0.142857	0.142857	0.142857	0.142857	0.142857	0.142857	0.142857	0.142857	0.142857	0.142857	0.142857
		M00096	C5 isoprenoid biosynthesis, non-mevalonate pathway	0.875	1	1	1	1	1	1	1	1	1	1	1	1	1	1	1	1	1	1	1	1	1	1	1	1	1	1	1	1	1	1	1	1	1	1	1	1
		M00364	C10-C20 isoprenoid biosynthesis, bacteria	1	1	1	1	1	1	1	1	1	1	1	1	1	1	1	1	1	1	1	1	1	1	1	1	1	1	1	1	1	1	1	1	1	1	1	1	1
		M00365	C10-C20 isoprenoid biosynthesis, archaea	0.5	0.5	0.5	0.5	0.5	0.5	0.5	0.5	0.5	0.5	0.5	0.5	0.5	0.5	0.5	0.5	0.5	0.5	0.5	0.5	0.5	0.5	0.5	0.5	0.5	0.5	0.5	0.5	0.5	0.5	0.5	0.5	0.5	0.5	0.5	0.5	0.5
		M00366	C10-C20 isoprenoid biosynthesis, plants	0.5	0.5	0.5	0.5	0.5	0.5	0.5	0.5	0.5	0.5	0.5	0.5	0.5	0.5	0.5	0.5	0.5	0.5	0.5	0.5	0.5	0.5	0.5	0.5	0.5	0.5	0.5	0.5	0.5	0.5	0.5	0.5	0.5	0.5	0.5	0.5	0.5
		M00367	C10-C20 isoprenoid biosynthesis, non-plant eukaryotes	0.333333	0.333333	0.333333	0.333333	0.333333	0.333333	0.333333	0.333333	0.333333	0.333333	0.333333	0.333333	0.333333	0.333333	0.333333	0.333333	0.333333	0.333333	0.333333	0.333333	0.333333	0.333333	0.333333	0.333333	0.333333	0.333333	0.333333	0.333333	0.333333	0.333333	0.333333	0.333333	0.333333	0.333333	0.333333	0.333333	0.333333
		M00849	C5 isoprenoid biosynthesis, mevalonate pathway, archaea	0.1875	0.1875	0.1875	0.1875	0.1875	0.1875	0.1875	0.1875	0.1875	0.1875	0.1875	0.1875	0.1875	0.1875	0.1875	0.1875	0.1875	0.1875	0.1875	0.1875	0.1875	0.1875	0.1875	0.1875	0.1875	0.1875	0.1875	0.1875	0.1875	0.1875	0.1875	0.1875	0.1875	0.1875	0.1875	0.1875	0.1875
	Plant terpenoid biosynthesis	M00097	beta-Carotene biosynthesis, GGAP => beta-carotene	0.666667	0.666667	0.666667	0.666667	0.666667	0.666667	0.666667	0.666667	0.666667	0.666667	0.666667	0.666667	0.666667	0.666667	0.666667	0.666667	0.666667	0.666667	0.666667	0.666667	0.666667	0.666667	0.666667	0.666667	0.666667	0.666667	0.666667	0.666667	0.666667	0.666667	0.666667	0.666667	0.666667	0.666667	0.666667	0.666667	0.666667
	Enediyne biosynthesis	M00824	9-membered enediyne core biosynthesis, malonyl-CoA => 3-hydroxyhexadeca-4,6,8,10,12,14-hexaenoyl-ACP => 9-membered enediyne core	0	0	0	0	0	0	0	0	0	0	0	0	0	0.5	0	0	0	0	0.5	0	0	0	0	0	0	0	0	0	0	0	0	0	0	0	0	0	0
		M00825	10-membered enediyne core biosynthesis, malonyl-CoA => 3-hydroxyhexadeca-4,6,8,10,12,14-hexaenoyl-ACP => 10-membered enediyne core	0	0	0	0	0	0	0	0	0	0	0	0	0	0.5	0	0	0	0	0.5	0	0	0	0	0	0	0	0	0	0	0	0	0	0	0	0	0	0
	Polyketide sugar unit biosynthesis	M00793	dTDP-L-rhamnose biosynthesis	1	1	1	1	1	1	1	1	1	1	1	1	1	1	1	1	1	1	1	1	1	1	1	1	1	1	1	1	1	1	1	1	1	1	1	1	1
		M00798	dTDP-L-mycarose biosynthesis	0	0	0	0	0	0	0	0	0	0.2	0.2	0	0	0	0	0	0	0	0	0.2	0	0	0	0	0	0	0	0	0	0	0	0	0	0	0	0	0
Biosynthesis of other secondary metabolites	Biosynthesis of phytochemical compounds	M00039	Monolignol biosynthesis, phenylalanine/tyrosine => monolignol	0.1	0.1	0.1	0.1	0.1	0.1	0.1	0.1	0.1	0.1	0.1	0.1	0.1	0.1	0.1	0.1	0.1	0.1	0.1	0.1	0.1	0.1	0.1	0.1	0.1	0.1	0.1	0.1	0.1	0.1	0.1	0.1	0.1	0.1	0.1	0.1	0.1
		M00953	Mugineic acid biosynthesis, methionine => 3-epihydroxymugineic acid	0.2	0.2	0.2	0.2	0.2	0.2	0.2	0.2	0.2	0.2	0.2	0.2	0.2	0.2	0.2	0.2	0.2	0.2	0.2	0.2	0.2	0.2	0	0.2	0.2	0.2	0.2	0.2	0.2	0.2	0.2	0.2	0.2	0.2	0.2	0.2	0.2
	Biosynthesis of beta-lactams	M00736	Nocardicin A biosynthesis, L-pHPG + arginine + serine => nocardicin A	0.083333	0.083333	0.083333	0	0.083333	0	0.083333	0.083333	0	0	0	0.083333	0.083333	0	0.083333	0.083333	0.083333	0.083333	0.083333	0.083333	0.083333	0.083333	0.083333	0.083333	0.083333	0.083333	0.083333	0.083333	0.083333	0.083333	0.083333	0.083333	0.083333	0.083333	0.083333	0.083333	0.083333
	Biosynthesis of other bacterial compounds	M00835	Pyocyanine biosynthesis, chorismate => pyocyanine	0.142857	0.142857	0.142857	0.142857	0.142857	0.142857	0.142857	0.142857	0.142857	0.142857	0.142857	0.142857	0.142857	0.142857	0.142857	0.142857	0.142857	0.142857	0.142857	0.142857	0.142857	0.142857	0.142857	0.142857	0.142857	0.142857	0.142857	0.142857	0.142857	0.142857	0.142857	0.142857	0.142857	0.142857	0.142857	0.142857	0.142857
		M00906	Ethynylserine biosynthesis, lysine => ethynylserine	0	0	0.2	0	0	0	0	0	0	0	0	0	0	0	0	0	0	0	0	0	0	0	0	0	0	0	0	0	0	0	0	0	0	0	0	0	0
	Biosynthesis of other antibiotics	M00848	Aurachin biosynthesis, anthranilate => aurachin A	0.03125	0.03125	0.03125	0.03125	0.03125	0.03125	0.03125	0.03125	0.03125	0.03125	0.03125	0.03125	0.03125	0.03125	0.03125	0.03125	0.03125	0.03125	0.03125	0.03125	0.03125	0.03125	0.03125	0.03125	0.03125	0.03125	0.03125	0.03125	0.03125	0.03125	0.03125	0.03125	0.03125	0.03125	0.03125	0.03125	0.03125
Xenobiotics biodegradation	Aromatics degradation	M00568	Catechol ortho-cleavage, catechol => 3-oxoadipate	0.25	0.25	0.25	0	0	0.25	0	0	0.25	0.25	0.25	0.25	0.25	0.25	0.25	0.25	0.25	0	0.25	0.25	0.25	0.25	0.25	0.25	0.25	0.25	0	0	0.25	0.25	0.25	0.25	0	0	0	0	0
		M00569	Catechol meta-cleavage, catechol => acetyl-CoA / 4-methylcatechol => propanoyl-CoA	0	0	0	0	0	0	0	0.142857	0	0	0	0	0	0	0	0	0	0	0	0	0	0	0	0	0	0	0	0	0	0	0	0	0	0	0	0	0

Kanehisa, Minoru, and Yoko Sato. "KEGG Mapper for Inferring Cellular Functions from Protein Sequences." Protein Science: A Publication of the Protein Society 29, no. 1 (January 2020): 28–35. https://doi.org/10.1002/pro.3711.

Table S4

Functional enrichment analysis carried out on the R1 and R2 clades using the pangenomic workflow by anvio (Eren *et al.*, 2021). The analysis reports the main differences in the COG20 functions. Only entries with adjusted_q values < 0.01 are reported, distinguishing the COG20 classified categories (black) from the unclassified or "general function" categories (gray). p_R1, p_R2, and N_R1, N_R2: fraction of assemblies with the COG20 function, and number of assemblies in the R1 and R2 clades, respectively.

COG20_FUNCTION	COG20_CATEGORY	Adjusted _q	associated_ groups	p_R1	p_R2	N_R1	N_R2
Nicotinic acid phosphoribosyltransferase	Coenzyme transport and metabolism	0.00966	R1	0.8846	0.2727	26	11
Antitoxin component HicB of the HicAB toxin-antitoxin system	Defense mechanisms	0.00003	R1	0.9615	0.0909	26	11
Virulence-associated protein VapD, endoribonuclease (VapD)	Defense mechanisms	0.00323	R1	0.7308	0	26	11
Cu/Ag efflux pump CusA (CusA)	Inorganic ion transport and metabolism	0.00002	R1	0.9231	0	26	11
Uncharacterized phage-associated protein, contains DUF4065 domain (GepA)	Mobilome: prophages, transposons	0.00008	R1	0.8846	0	26	11
Cytosine/adenosine deaminase or related metal-dependent hydrolase (SsnA)	Nucleotide transport and metabolism|General function prediction only	0.00001	R1	0.9615	0	26	11
Phospholipase/lecithinase/hemolysin (PDB:6JL1)|Uncharacterized conserved protein YhjY, contains autotransporter beta-barrel domain (YhjY)	Nucleotide transport and metabolism|General function prediction only	0.00001	R1	0.9615	0	26	11
Predicted ATP-dependent endonuclease of the OLD family, contains P-loop ATPase and TOPRIM domains (YbjD)	Replication, recombination and repair	0.00711	R1	0.6923	0	26	11
NADH/NAD ratio-sensing transcriptional regulator Rex (Rex)	Transcription	0.00169	R1	0.7692	0	26	11
Ribonuclease PH (Rph)	Translation, ribosomal structure and biogenesis	0.00000	R1	1	0	26	11
RNase P protein component (RnpA)	Translation, ribosomal structure and biogenesis	0.00069	R1	0.9231	0.1818	26	11
Pectate lyase (PelB)	Carbohydrate transport and metabolism	0.00349	R2	0.0385	0.6364	26	11
6-phosphogluconolactonase, cycloisomerase 2 family (Pgl) (PDB:1JOF)|Ca2+-binding protein, RTX toxin-related	Carbohydrate transport and metabolism|Secondary metabolites biosynthesis, transport and catabolism	0.00271	R2	0	0.5455	26	11
Chromosome segregation ATPase Smc (Smc) (PDB:5XG3)|5-methylcytosine-specific restriction endonuclease McrBC, GTP-binding regulatory subunit McrB (McrB)	Cell cycle control, cell division, chromosome partitioning|Defense mechanisms	0.00966	R2	0	0.4545	26	11
Surface polysaccharide O-acyltransferase WecH (WecH)	Cell wall/membrane/envelope biogenesis	0.00966	R2	0	0.4545	26	11
Glycosyltransferase involved in cell wall bisynthesis (WcaA) (PDB:5MLZ)|Predicted SAM-depedendent methyltransferase	Cell wall/membrane/envelope biogenesis|General function prediction only	0.00069	R2	0	0.6364	26	11
Pyrimidine reductase, riboflavin biosynthesis (RibD)	Coenzyme transport and metabolism	0.00271	R2	0	0.5455	26	11
Toxin component of the Txe-Axe toxin-antitoxin module, Txe/YoeB family	Defense mechanisms	0.00003	R2	0	0.8182	26	11
Antitoxin component HigA of the HigAB toxin-antitoxin module, contains an N-terminal HTH domain (HigA)	Defense mechanisms	0.00069	R2	0	0.6364	26	11
Acetate kinase (AckA)	Energy production and conversion	0.00069	R2	0	0.6364	26	11
NAD(P)-dependent dehydrogenase, short-chain alcohol dehydrogenase family (FabG)|Cyclopropane fatty-acyl-phospholipid synthase and related methyltransferases (Cfa)|Acyl transferase domain in polyketide synthase (PKS) enzymes (PksD)	Lipid transport and metabolism|Lipid transport and metabolism|Secondary metabolites biosynthesis, transport and catabolism	0.00271	R2	0	0.5455	26	11
Acyl carrier protein (AcpP)|EntF, seryl-AMP synthase component of non-ribosomal peptide synthetase (EntF)|Uncharacterized conserved protein, contains a NRPS condensation (elongation) domain	Lipid transport and metabolism|Secondary metabolites biosynthesis, transport and catabolism	0.00271	R2	0	0.5455	26	11

Cytidine deaminase (Cdd)	Nucleotide transport and metabolism	0.00271	R2	0	0.5455	26	11
V8-like Glu-specific endopeptidase (eMpr)	Posttranslational modification, protein turnover, chaperones	0.00014	R2	0	0.7273	26	11
Periplasmic serine protease, S1-C subfamily, contain C-terminal PDZ domain (DegQ)|Flp pilus assembly protein TadD, contains TPR repeats (TadD)	Posttranslational modification, protein turnover, chaperones|Intracellular trafficking, secretion, and vesicular transport|Extracellular structures	0.00966	R2	0	0.4545	26	11
Replication fork clamp-binding protein CrfC (dynamin-like GTPase family) (CrfC)	Replication, recombination and repair	0.00000	R2	0	1	26	11
DNA-binding transcriptional regulator, MerR family (SoxR)	Transcription	0.00966	R2	0	0.4545	26	11
tRNA isopentenyl-2-thiomethyl-A-37 hydroxylase MiaE (synthesis of 2-methylthio-cis-ribozeatin) (MiaE)	Translation, ribosomal structure and biogenesis	0.00002	R2	0.0769	1	26	11
Uncharacterized protein, contains SWIM-type Zn finger domain	Function unknown	0.00014	R1	1	0.2727	26	11
Predicted RNA binding protein YcfA, dsRBD-like fold, HicA-like mRNA interferase family (YcfA)	General function prediction only	0.00014	R1	1	0.2727	26	11
Predicted extracellular nuclease	General function prediction only	0.00069	R1	0.8077	0	26	11
Predicted nucleotidyltransferase	General function prediction only	0.00711	R1	0.6923	0	26	11
Uncharacterized conserved protein YcgG, contains conserved FPC and CPF motifs (YcgG)	Function unknown	0.00271	R2	0	0.5455	26	11
Uncharacterized CoxE-like protein, contains von Willebrand factor type A (vWA) domain (CoxE2)	Function unknown	0.00966	R2	0	0.4545	26	11
Uncharacterized membrane protein YhaH, DUF805 family (yhaH)	Function unknown	0.00966	R2	0	0.4545	26	11
Predicted carbamoyl transferase, NodU family	General function prediction only	0.00081	R2	0.0385	0.7273	26	11
Predicted sugar epimerase, cupin superfamily (CFF1)	General function prediction only	0.00271	R2	0	0.5455	26	11
SET domain-containing protein (function unknown) (SET)	General function prediction only	0.00966	R2	0	0.4545	26	11
Predicted antitoxin, contains HTH domain	General function prediction only	0.00966	R1	1	0.5455	26	11
Predicted extracellular nuclease|Ca2+-binding protein, RTX toxin-related (PDB:1AF0)	General function prediction only|Secondary metabolites biosynthesis, transport and catabolism	0.00966	R2	0	0.4545	26	11
GTPase SAR1 family domain (Gem1) (PDB:4C0J)|Leucine-rich repeat (LRR) protein (LRR)	General function prediction only|Transcription	0.00966	R2	0	0.4545	26	11

Adaptations to different geographic regions can be reflected by the presence of different defence mechanisms in the two groups, such as HicB and VapD (R1), and Txe and HigA (R2), which are widely present in bacterial genomes, often in association with mobile genetic elements, and involved in the control of stress response (Leplae *et al.*, 2011; Ren *et al.*, 2012; Thomet *et al.*, 2019; Fraikin *et al.*, 2020; Song *et al.*, 2021). Furthermore, the R1 group showed an exclusive enrichment part of the mobilome associated with prophages and transposons, indicative of specific interactions with phages in different geographical regions (Jaskulska *et al.*, 2022). Similarly, regarding metabolism, differences in functions in the carbohydrate transport and metabolism category can be linked to nutrient cycling and utilization of external organic compounds (Schwarz *et al.*, 2020) differentially developed in the R2 group. Regarding the inorganic ion transport, the exclusive presence of metal efflux pumps (CusA) in R1 is indicative of adaptations to specific resistance to excess of different toxic metal ions (Hahn *et al.*, 2013). Nevertheless, homeostasis maintenance for

essential and non-essential metals (Cavet *et al.*, 2003) in both groups is identified by the presence of other efflux pumps. Other differences between the two groups are linked to adaptations to specific environments like regulation of gene expression (category transcription and translation in R1 and R2), and maintenance of cell wall functionality (category cell wall/membrane/envelope biogenesis in R2). The remaining differences are indicative of a wide variety of metabolic and cellular processes that express different abilities to utilize different resources under different ecological environments or stress conditions, e.g. including, in the R2 group, sustained metabolite biosynthesis and survival under dark conditions favoured by Acetate kinase (AckA) (Ueda *et al.*, 2016).

Cavet, J.S., Borrelly, G.P.M., and Robinson, N.J. (2003) Zn, Cu and Co in cyanobacteria: selective control of metal availability. *FEMS Microbiol Rev* **27**: 165–181.

Eren, A.M., Kiefl, E., Shaiber, A., Veseli, I., Miller, S.E., Schechter, M.S., et al. (2021) Community-led, integrated, reproducible multi-omics with anvi’o. *Nat Microbiol* **6**: 3–6.

Fraikin, N., Goormaghtigh, F., and Van Melderen, L. (2020) Type II Toxin-Antitoxin Systems: Evolution and Revolutions. *J Bacteriol* **202**: 10.1128/jb.00763-19.

Hahn, A., Stevanovic, M., Mirus, O., Lytvynenko, I., Pos, K.M., and Schleiff, E. (2013) The Outer Membrane TolC-like Channel HgdD Is Part of Tripartite Resistance-Nodulation-Cell Division (RND) Efflux Systems Conferring Multiple-drug Resistance in the Cyanobacterium Anabaena sp. PCC7120. *J Biol Chem* **288**: 31192–31205.

Jaskulska, A., Šulčius, S., Kokociński, M., Koreivienė, J., Nájera, A.F., and Mankiewicz-Boczek, J. (2022) Cyanophage Distribution Across European Lakes of the Temperate-Humid Continental Climate Zone Assessed Using PCR-Based Genetic Markers. *Microb Ecol* **83**: 284–295.

Leplae, R., Geeraerts, D., Hallez, R., Guglielmini, J., Drèze, P., and Van Melderen, L. (2011) Diversity of bacterial type II toxin–antitoxin systems: a comprehensive search and functional analysis of novel families. *Nucleic Acids Res* **39**: 5513–5525.

Ren, D., Walker, A.N., and Daines, D.A. (2012) Toxin-antitoxin loci vapBC-1 and vapXD contribute to survival and virulence in nontypeable *Haemophilus influenzae*. *BMC Microbiol* **12**: 263.

Schwarz, A., Walther, J., Geib, D., Witthohn, M., Strieth, D., Ulber, R., and Muffler, K. (2020) Influence of heterotrophic and mixotrophic cultivation on growth behaviour of terrestrial cyanobacteria. *Algal Res* **52**: 102125.

Song, Y., Zhang, S., Luo, G., Shen, Y., Li, C., Zhu, Y., et al. (2021) Type II Antitoxin HigA Is a Key Virulence Regulator in *Pseudomonas aeruginosa*. *ACS Infect Dis* **7**: 2930–2940.

Thomet, M., Trautwetter, A., Ermel, G., and Blanco, C. (2019) Characterization of HicAB toxin-antitoxin module of *Sinorhizobium meliloti*. *BMC Microbiol* **19**: 10.

Ueda, S., Kawamura, Y., Iijima, H., Nakajima, M., Shirai, T., Okamoto, M., et al. (2016) Anionic metabolite biosynthesis enhanced by potassium under dark, anaerobic conditions in cyanobacteria. *Sci Rep* **6**: 32354.
