# Supplementary material for: Phylogenomic and Pangenomic Assessment of a Mediterranean Strain of Raphidiopsis raciborskii Extends Knowledge of the Global Distribution and Characteristics of a Potentially Toxigenic Cyanobacterium
Source: Environ Microbiol Rep. 2025 May 19;17(3):e70098. doi: 10.1111/1758-2229.70098 (PMC12089652; doi:10.1111/1758-2229.70098)
Supplement: Supplementary file 2 — Appendix S2. Figure S1 (A) Location of Lake Trasimeno in central Italy and sampling sites at Castiglione del Lago (red circle) and Passignano sul Trasimeno (TRS30) and Magione (TRS35) (yellow circles) (Table 1). The Raphidiopsis Lake Trasimeno strain was collected at the Castiglione del Lago sampling site shown in the photo (September 12, 2023); the Polvese Island can be seen in the background. Complete physical and chemical analyses were performed on samples collected at Passignano sul Trasimeno and Magione by the Environmental Agency of the Umbria Region (September 11, 2023) (https://apps.arpa.umbria.it/acqua/qualita‐acque‐superficiali). (B) Two single filaments of Raphidiopsis raciborskii developed in culture conditions from the isolation of an individual from the sample collected at Castiglione del Lago; heterocytes are visible at the end of the filaments; yellow scale bars, 5 μm. Figure S2. Presence of each gene family in the 37 genomes analysed in this work based on the anvio and panaroo analyses. The classification scheme used is that proposed by panaroo. Core (99% ≤ strains ≤ 100%), Soft core (95% ≤ strains < 99%), Shell (15% ≤ strains < 95%) and Cloud (0% ≤ strains < 15%). Figure S3. (A) Phylogenomic tree of Raphidiopsis based on the 1526 single‐copy core gene clusters (SCGs) identified in the pangenomic analysis; the tree was rooted using midpoint rooting; UFBoot, Ultrafast bootstrap values. Other details as in Figure 1. (B) Tanglegram comparing the topology of the two phylogenomic trees obtained by the gtdbtk analysis of Figure 1 (left) and the analysis in the panel (A) based on the 1526 SCGs (right); where relevant, branches were rotated around the nodes to maximise the correspondence between the tips. [file EMI4-17-e70098-s002.pdf]

Phylogenomic and pangenomic assessment of a Mediterranean strain of *Raphidiopsis raciborskii* extends knowledge of the global distribution and characteristics of a potentially toxigenic cyanobacterium

Nico Salmaso, Leonardo Cerasino, Margherita Di Brizio, Massimo Pindo, Adriano Boscaini

Supplementary Figures

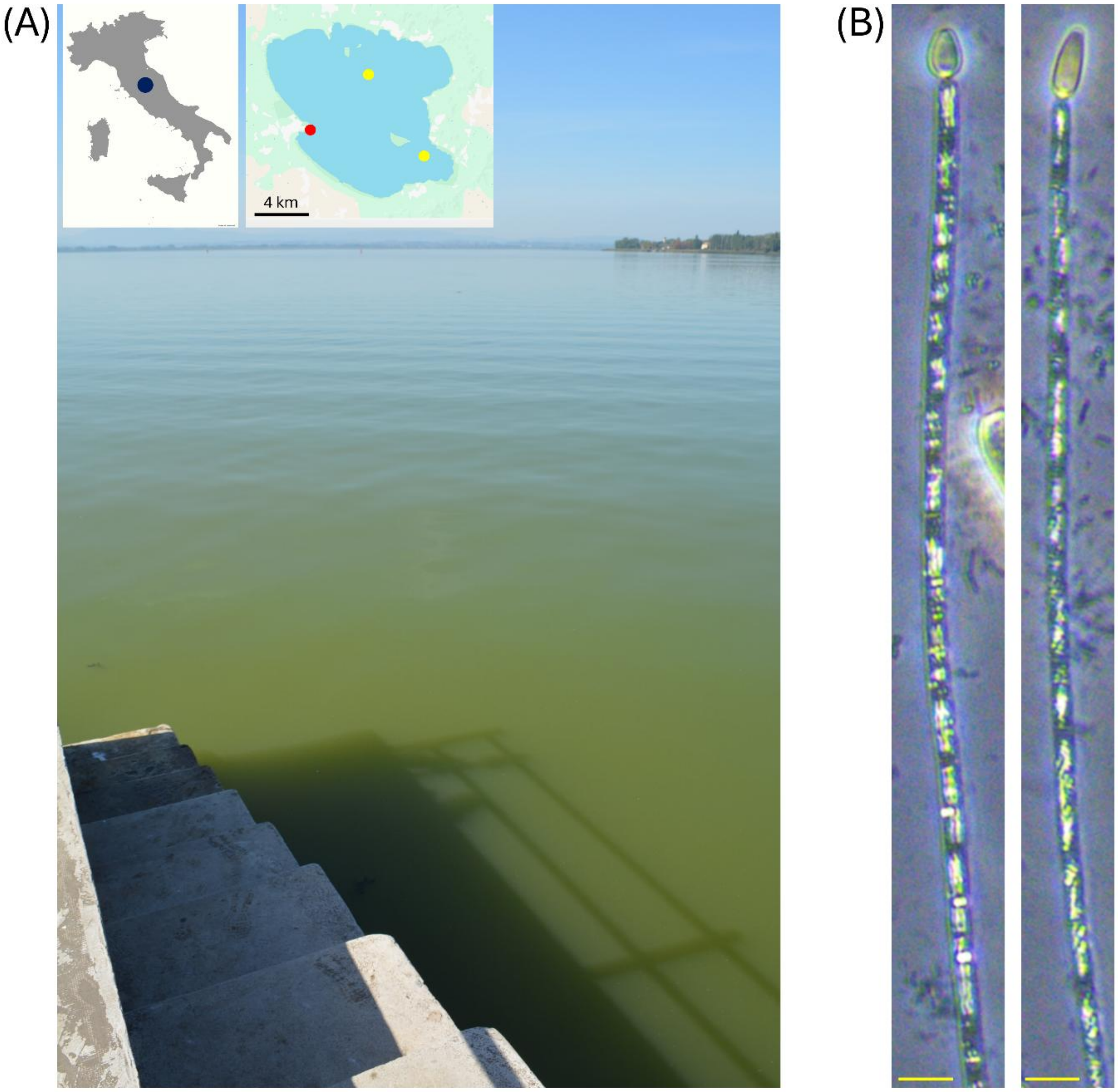

Figure S1

(A) Location of Lake Trasimeno in central Italy and sampling sites at Castiglione del Lago (red circle) and Passignano sul Trasimeno (TRS30) and Magione (TRS35) (yellow circles) (Table 1). The *Raphidiopsis* Lake Trasimeno strain was collected at the Castiglione del Lago sampling site shown in the photo (September 12, 2023); the Polvese Island can be seen in the background. Complete physical and chemical analyses were performed on samples collected at Passignano sul Trasimeno and Magione by the Environmental Agency of the Umbria Region (September 11, 2023) (https://apps.arpa.umbria.it/acqua/qualita-acque-superficiali). (B) Two single filaments of *Raphidiopsis raciborskii* developed in culture conditions from the isolation of an individual from the sample collected at Castiglione del Lago; heterocytes are visible at the end of the filaments; yellow scale bars, 5 µm.

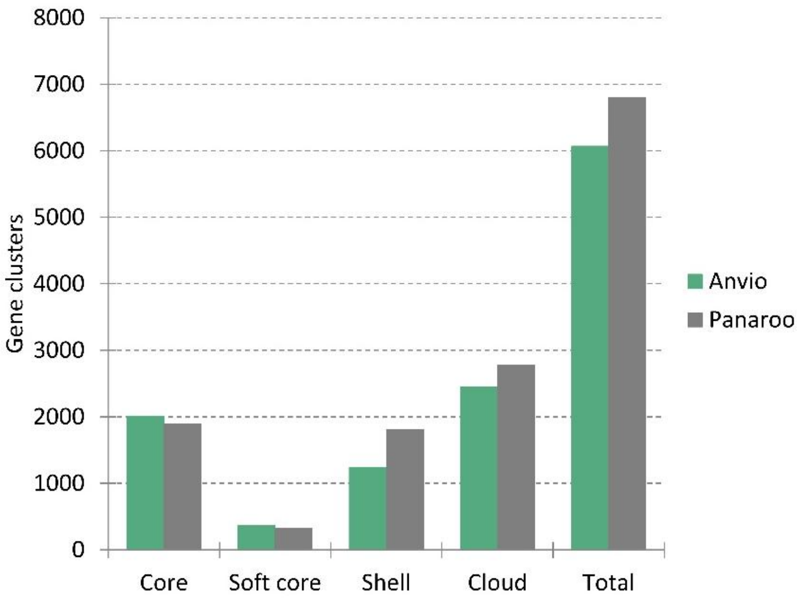

Figure S2

Presence of each gene family in the 37 genomes analysed in this work, based on the anvio and panaroo analyses. The classification scheme used is that proposed by panaroo. Core (99% <= strains <= 100%), Soft core (95% <= strains < 99%), Shell (15% <= strains < 95%) and Cloud (0% <= strains < 15%).

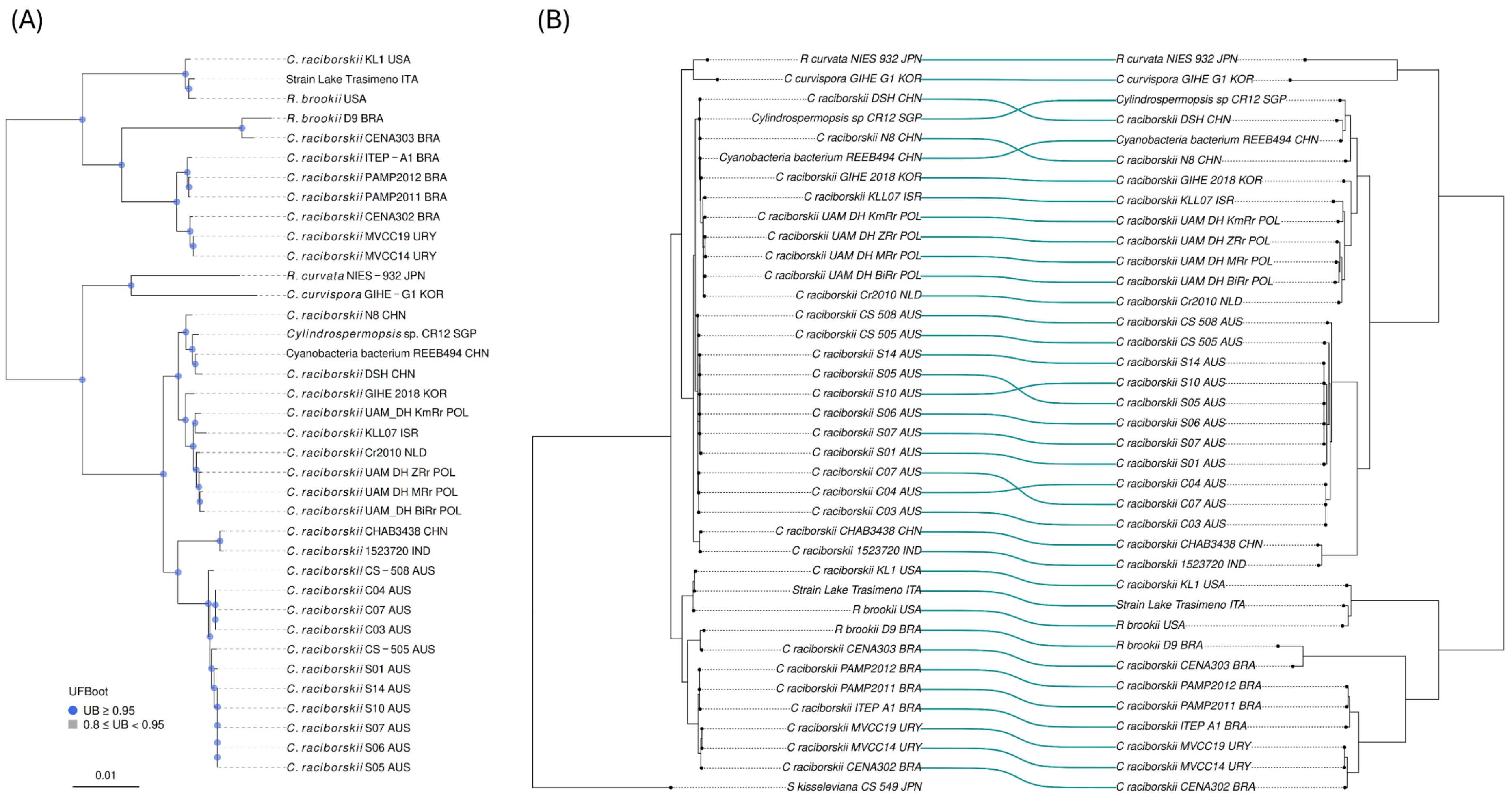

Figure S3

(A) Phylogenomic tree of *Raphidiopsis* based on the 1526 single-copy core gene clusters (SCGs) identified in the pangenomic analysis; the tree was rooted using midpoint rooting; UFBoot, Ultrafast bootstrap values. Other details as in Figure 1. (B) Tanglegram comparing the topology of the two phylogenomic trees obtained by the gtdbtk analysis of Figure 1 (left) and the analysis in the panel (A), based on the 1526 SCGs (right); where relevant, branches were rotated around the nodes to maximise the correspondence between the tips.
